# Dysregulated immune and metabolic pathways are associated with poor survival in adult acute myeloid leukemia with *CEBPA* bZIP in-frame mutations

**DOI:** 10.1038/s41408-023-00975-8

**Published:** 2024-01-23

**Authors:** Feng-Ming Tien, Chi-Yuan Yao, Xavier Cheng-Hong Tsai, Min-Yen Lo, Chien-Yuan Chen, Wan-Hsuan Lee, Chien-Chin Lin, Yuan-Yeh Kuo, Yen-Ling Peng, Mei-Hsuan Tseng, Yu-Sin Wu, Ming-Chih Liu, Liang-In Lin, Ming-Kai Chuang, Bor-Sheng Ko, Ming Yao, Jih-Luh Tang, Wen-Chien Chou, Hsin-An Hou, Hwei-Fang Tien

**Affiliations:** 1 Division of Hematology, Department of Internal Medicine, National Taiwan University Hospital, Taipei, Taiwan; 2 Graduate Institute of Clinical Medicine, College of Medicine, National Taiwan University, Taipei, Taiwan; 3 Department of Laboratory Medicine, National Taiwan University Hospital, Taipei, Taiwan; 4 Division of Hematology, Department of Internal Medicine, National Taiwan University Hospital Yunlin Branch, Yunlin, Taiwan; 5 Department of Internal Medicine, National Taiwan University Hospital, Hsin-Chu Branch, Hsin-Chu Taiwan; 6 Tai-Chen Cell Therapy Center, National Taiwan University, Taipei, Taiwan; 7 Department of Nursing, National Taiwan University Cancer Center, Taipei, Taiwan; 8 Department of Pathology, National Taiwan University Hospital, Taipei, Taiwan; 9 Department of Clinical Laboratory Sciences and Medical Biotechnology, College of Medicine, National Taiwan University, Taipei, Taiwan; 10 Department of Hematological Oncology, National Taiwan University Cancer Center, Taipei, Taiwan; 11 Department of Internal Medicine, Far-Eastern Memorial Hospital, New Taipei City, Taiwan

**Keywords:** Acute myeloid leukaemia, Cancer genomics

## Abstract

Acute myeloid leukemia (AML) with *CEBPA* bZIP in-frame mutations (*CEBPA*^bZIP-inf^) is classified within the favorable-risk group by the 2022 European LeukemiaNet (ELN-2022). However, heterogeneous clinical outcomes are still observed in these patients. In this study, we aimed to investigate the mutation profiles and transcriptomic patterns associated with poor outcomes in patients with *CEBPA*^bZIP-inf^. One hundred and thirteen *CEBPA*^bZIP-inf^ patients were identified in a cohort of 887 AML patients homogeneously treated with intensive chemotherapy. Concurrent *WT1* or *DNMT3A* mutations significantly predicted worse survival in AML patients with *CEBPA*^bZIP-inf^. RNA-sequencing analysis revealed an enrichment of interferon (IFN) signaling and metabolic pathways in those with a shorter event-free survival (EFS). *CEBPA*^bZIP-inf^ patients with a shorter EFS had higher expression of IFN-stimulated genes (*IRF2, IRF5, OAS2*, and *IFI35*). Genes in mitochondrial complexes I (*NDUFA12* and *NDUFB6*) and V (*ATP5PB* and *ATP5IF1*) were overexpressed and were associated with poorer survival, and the results were independently validated in the TARGET AML cohort. In conclusion, concurrent *WT1* or *DNMT3A* mutations and a dysregulated immune and metabolic state were correlated with poor survival in patients with *CEBPA*^bZIP-inf^, and upfront allogeneic transplantation may be indicated for better long-term disease control.

## Introduction

Acute myeloid leukemia (AML) with mutant CCAAT/enhancer binding protein α (*CEBPA*) accounts for 5.1–18.9% of patients with AML; a higher incidence rate is observed in patients with AML from Asia than in those from Western countries [1–7]. We and others have showed that *CEBPA* double mutation (*CEBPA*^dm^) predicted better overall survival (OS) whereas *CEBPA* single mutation (*CEBPA*^sm^) did not have this survival advantage [8–11]. Accordingly, *CEBPA*^dm^ AML has been recognized as a separate entity in the 2016 World Health Organization (WHO) Classification (WHO-2016) [12] and a favorable-risk category in 2017 European LeukemiaNet (ELN) recommendations. Concomitant *WT1* mutations or *CSF3R* mutations helped predict a poor prognosis in patients with *CEBPA*^dm^ [7, 13].

Recently, it was discovered that the favorable outcomes of patients with *CEBPA* mutations are confined to those with in-frame mutations in bZIP (*CEBPA*^bZIP-inf^), no matter whether they have *CEBPA*^sm^ or *CEBPA*^dm^ [4, 14]. These findings impact the current 5th WHO Classification (WHO-2022) [15], 2022 ELN recommendations (ELN-2022) [16] and International Consensus Classification (ICC) [17], resulting in the changing of the category, “AML with biallelic mutations of *CEBPA*” into “AML with in-frame bZIP mutations of *CEBPA*.” However, the 5-year event-free survival (EFS) was less than 50% [4], and the cumulative incidence of relapse was approaching 40% in this category [18]. It remains largely unknown how to explain the heterogeneous outcomes in this so-called “favorable category” at the mutational and transcriptomic level. The identification of dysregulated pathways that are independent of the mutational background, may provide novel options for therapeutic targeting.

In this study, we aimed to investigate the prognostic relevance of mutations and transcriptomics in AML patients with *CEBPA*^bZIP-inf^ from a large cohort of 887 unselected de novo non-M3 AML patients. Our study showed that patients with poor survival were characterized by enrichment of interferon (IFN) pathways and oxidative phosphorylation. These data provide insights into non-genetic heterogeneity that may guide precise risk stratification and hold therapeutic potential in AML with *CEBPA*^bZIP-inf^. The findings were also well-validated in the TARGET AML cohort.

## Methods and materials

### Subjects

We consecutively enrolled 887 newly diagnosed de novo non-M3 AML patients who received standard chemotherapy at the National Taiwan University Hospital (NTUH). Diagnosis and classification of AML were made according to the 2022 ICC [12] and WHO-2022 [15]. Detailed patient characteristics and treatment regimens were summarized in the Supplementary Data [19]. This study was approved by the Institutional Review Board of the NTUH, and written informed consent was obtained from all participants in accordance with the Declaration of Helsinki (approval number: 201709072RINC and 202109078RINB).

### Cytogenetics

Chromosomal analyses were performed as described previously [20]. Karyotypes were classified using Medical Research Council (MRC)-defined risk groups [21].

### Mutation analysis

Gene mutations were examined via targeted next-generation sequencing (NGS), using the TruSight myeloid sequencing panel (Illumina, San Diego, CA, USA), which included 15 full exon genes and 39 oncogenic hotspot genes. HiSeq platform (Illumina, San Diego, CA, USA) was used for sequencing with a median reading depth of 12000×. Owing to suboptimal sequencing sensitivity, we verified *CEBPA* mutations via Sanger sequencing. Analysis of *FLT3*-ITD was performed via polymerase chain reaction (PCR), followed by fluorescence capillary electrophoresis and that of *KMT2A*-PTD, by PCR followed by Sanger sequencing [22].

### RNA-sequencing (RNA-seq) analysis

Bone marrow (BM) mononuclear cells from 136 AML patients (36 with *CEBPA*^bZIP-inf^, 11 with *CEBPA*^nonbZIP-inf^, 89 with wild type of *CEBPA* and normal karyotype*, CEBPA*^wt^) were performed with RNA-seq. The purified RNA was used to prepare the sequencing libraries using the TruSeq Stranded mRNA Library Prep Kit (Illumina, San Diego, CA, USA) following the manufacturer’s recommendations. The qualified libraries were then sequenced on Illumina NovaSeq 6000 with 150 bp paired-end mode. Trimming of adaptor sequences and removal of reads of low quality were performed using cutadapt (v2.3). Quantified reads were aligned to the human genome (GRCh38.p12) by STAR (v2.6.1 a) [23], and then gene-level read counts were generated based on the annotations of Gencode (v28) [24]. The read counts cross all samples were normalized using the trimmed mean of the *M* values method as implemented in the calcNormFactors function of edgeR package [25], and gene expression in terms of log_2_(CPM + 1) (counts per million reads) was computed for further analysis. We used limma to assess the differentially expressed genes (DEGs) between conditions [26]. The linear model was fit to the normalized expression data using a design matrix contrasting patients with short and long EFS. Log2 fold changes (logFC) were computed, along with adjusted *P* values (false discovery rate, FDR) corrected for multiple hypothesis testing using the Benjamini–Hochberg (BH) method.

We used Gene Set Enrichment Analysis (GSEA) software to investigate systematic enrichments of *CEBPA* mutation-governed expressional profiles with gene sets curated in the Molecular Signatures Database (MSigDB) [27]. The statistical significance of the degree of enrichment was assessed by a 1000-time random permutation test [28].

### External datasets

The gene expression data of GSE15210, derived from Affymetrix HG-U133 oligonucleotide microarrays (*n* = 61, 7 with *CEBPA*^dm^, 8 with *CEBPA*^sm^, and 46 with *CEBPA*^wt^), and the TCGA AML RNA-seq data generated by the Illumina HiSeq 2000 platform (*n* = 173) were downloaded for validation purposes [10, 29]. To evaluate the prognostic significance of mitochondria complexes in an independent AML patient cohort, RNA-seq, gene mutation and clinical data of the TARGET AML cohort (*n* = 493) were downloaded from the Genomic Data Commons Data Portal (https://portal.gdc.cancer.gov/) [30]. Data from patients in the AMLSG cohort, the UK-NCRI trials, the TCGA cohort and NTUH Yunlin Branch cohort with available molecular annotations were further used to validate the prognostic relevance of concurrent mutations in *CEBPA*^bZIP-inf^ [29, 31, 32].

### Statistical analysis

The discrete variables were compared using the chi-square tests, but if the expected values of contingency tables were smaller than 5, Fisher’s exact test was used. Mann–Whitney U tests were used to compare continuous variables and medians of distributions. OS was measured from the date of first diagnosis to the date of last follow-up or death from any cause. EFS was measured from the date of diagnosis until treatment failure, relapse from first complete remission (CR1) or death from any cause, whichever occurred first [33]. Multivariate Cox proportional hazard regression analysis was used to investigate independent prognostic factors for OS and EFS. A *P* value < 0.05 was considered statistically significant. Statistical analyses were performed in the SPSS 23 (SPSS Inc., Chicago, IL, USA) and R software 4.3.1 (R Foundation for Statistical Computing, Vienna, Austria).

## Results

### Patient characteristics and clinical outcomes

A total of 142 patients (16%) were identified as having *CEBPA* mutations, including 113 (12.7%) with *CEBPA*^bZIP-inf^ and 29 (3.3%) patients with *CEBPA*^nonbZIP-inf^. The majority of *CEBPA*^bZIP-inf^ were *CEBPA*^dm^ (96 of 113, 85.0%) (Supplementary Table 1). The *CEBPA*^nonbZIP-inf^ consisted of 23 *CEBPA*^sm^, 4 *CEBPA*^dm^ outside the bZIP core region, and 2 *CEBPA*^dm^ with nonsense/out-of-frame bZIP mutations. Patients with *CEBPA*^bZIP-inf^ had significantly younger age, higher white blood cell (WBC) count and peripheral blood blast percentage at diagnosis compared with those with *CEBPA*^wt^. *CEBPA*^bZIP-inf^ also differed from *CEBPA*^nonbZIP-inf^ in terms of age, hemoglobin level, and peripheral blood blast percentage (Table 1).

**Table 1:** Comparison of clinical and laboratory features between patients with AML with different *CEBPA* mutational statuses.

Variables	*CEBPA*^wt^ (*n* = 745)	*CEBPA*^nonbZIP-inf^ (*n* = 29)	*CEBPA*^bZIP-inf^ (*n* = 113)	*P* value^a^	*P* value^b^	*P* value^c^
Sex				0.143	0.131	0.541
Male	385	19	67			
Female	360	10	46			
Age (year)	62.4 (18–78)	37.7 (19–75)	48.8 (18–73)	0.048	<0.0001	<0.0001
Lab data						
WBC (k/μL)	15.6 (0.16–627.8)	15.8 (1.68–301.1)	31.8 (2.15–405.7)	0.765	<0.0001	0.079
Hb (g/dL)	8.1 (3–15)	8.2 (5–11)	9.1 (4–15)	0.984	<0.0001	0.026
Platelet (k/μL)	50 (2–1017)	40 (6–712)	37 (5–250)	0.245	<0.0001	0.476
PB blast (%)	36 (0–99)	52.5 (6–99)	73.8 (0–99)	0.071	<0.0001	0.021
LDH (U/L)	686 (96–15000)	496 (162–2025)	758 (98–8280)	0.150	0.205	0.074
Cytogenetics						
Favorable	128 (17.2)	0	0	0.009	<0.0001	–
Interemediate	499 (67.0)	25 (86.2)	110 (97.3)	0.030	<0.0001	0.032
Unfavorable	95 (12.8)	2 (6.9)	2 (1.8)	0.565	<0.0001	0.185
NA	23	2	1			
2022 ICC						
t(8;21)(q22;q22.1)/*RUNX1::RUNX1T1*	89 (11.9)	0	0	0.066	<0.0001	–
inv(16)(p13.1q22) or t(16;16)(p13.1;q22)/*CBFB::MYH11*	39 (5.2)	0	0	0.392	0.013	–
t(9;11)(p21.3;q23.3)/*MLLT3*::*KMT2A*	12 (1.6)	0	0	>0.999	0.384	–
other *KMT2A* rearrangements	27 (3.6)	0	0	0.619	0.039	–
t(6;9)(p22.3;q34.1)/*DEK*::*NUP214*	5 (0.7)	0	0	>0.999	>0.999	–
inv(3)(q21.3q26.2) or t(3;3)(q21.3;q26.2)/*GATA2*; *MECOM*(*EVI1*)	11 (1.5)	0	0	>0.999	0.376	–
t(9;22)(q34.1;q11.2)/*BCR*::*ABL1*	2 (0.3)	0	0	>0.999	>0.999	–
*CEBPA*^bZIP-inf^	0	0	113 (100)	–	<0.0001	<0.0001
Mutated *NPM1*	164 (22.0)	7 (24.1)	0	0.787	<0.0001	<0.0001
Mutated *TP53*	35 (4.7)	2 (6.9)	0	>0.999	0.010	0.204
AML with myelodysplasia-related gene mutations	163 (21.9)	8 (27.6)	0	0.773	<0.0001	<0.0001
AML with myelodysplasia-related cytogenetic abnormalities	47 (6.3)	1 (3.4)	0	0.773	<0.0001	<0.0001
AML, NOS	151 (20.3)	11 (37.9)	0	0.006	<0.0001	<0.0001
WHO-2022						
*RUNX1-RUNX1T1* fusion	89 (11.9)	0	0	0.066	<0.0001	–
*CBFB-MYH11* fusion	39 (5.2)	0	0	0.392	0.013	–
*DEK-NUP214* fusion	5 (0.7)	0	0	>0.999	>0.999	–
*BCR*-*ABL1* fusion	4 (0.5)	0	0	>0.999	>0.999	–
*KMT2A* rearrangement	39 (5.2)	0	0	0.392	0.013	–
*MECOM* rearrangement	11 (1.5)	0	0	>0.999	0.376	–
*NUP98* rearrangement	13 (1.7)	0	0	>0.999	0.236	–
*CEBPA* mutation	0	6 (20.7)	113 (100)	<0.0001	<0.0001	<0.0001
*NPM1 mutation*	164 (22.0)	7 (24.1)	0	0.787	<0.0001	<0.0001
Myelodysplasia-related	182 (24.4)	6 (20.7)	0	0.645	<0.0001	<0.0001
AML, defined by differentiation	199 (26.7)	10 (34.5)	0	0.355	<0.0001	<0.0001
ELN-2022						
Favorable	213 (28.7%)	4 (13.8)	113 (100)	0.354	<0.0001	<0.0001
Intermediate	233 (31.2%)	13 (44.8)	0	0.251	<0.0001	<0.0001
Unfavorable	299 (40.1%)	12 (41.4)	0	0.812	<0.0001	<0.0001
CR1	545 (73.2)	21 (72.4)	105 (92.9)	0.916	<0.0001	0.005
Relapse	297 (54.5)	12 (57.1)	39 (37.1)	0.964	0.002	0.088
Allo-HSCT	320 (43.0)	14 (48.3)	45 (39.8)			
CR1	151 (47.2)	5 (35.7)	26 (57.8)	-	-	-
CR2	58 (18.1)	5 (35.7)	11 (24.4)	-	-	-
Others	111 (34.7)	4 (28.6)	8 (17.8)	-	-	-

*Allo-HSCT* allogeneic hematopoietic stem cell transplantation, *CR* complete remission, *ELN* European LeukemiaNet, *Hb* hemoglobin, *ICC* International Consensus Classification, *LDH* lactate dehydrogenase, *NA* not available, *WBC* white blood cell, *WHO* World Health Organization.

^a^*CEBPA*^nonbZIP-inf^ patients vs. *CEBPA*^wt^ patients.

^b^*CEBPA*^bZIP-inf^ patients vs. *CEBPA*^wt^ patients.

^c^*CEBPA*^bZIP-inf^ patients vs. CEBPA^nonbZIP-inf^ patients.

**Fig. 1:**
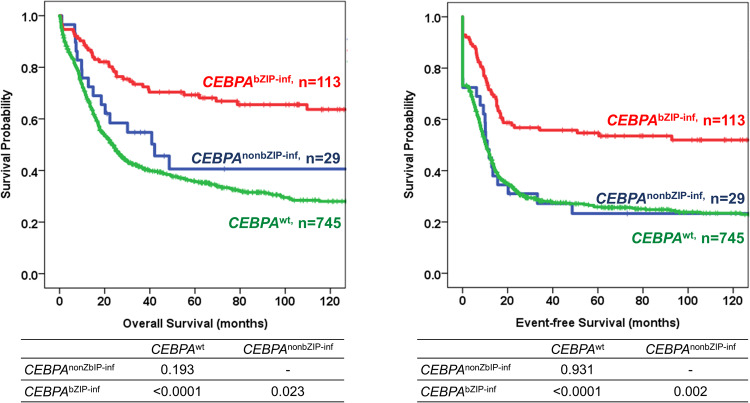
Survival outcomes stratified by *CEBPA* mutation statuses. *CEBPA*^bZIP-inf^ was associated with significantly better OS and EFS than *CEBPA*^nonbZIP-inf^ and *CEBPA*^wt^.

The CR1 rate was significantly higher for patients with *CEBPA*^bZIP-inf^ than those with *CEBPA*^nonbZIP-inf^ (92.9% vs. 72.4%, *P* = 0.005) and *CEBPA*^wt^ (92.9% vs. 73.2%, *P* < 0.0001). In contrast, the relapse rate was lower for patients with *CEBPA*^bZIP-inf^ than those with *CEBPA*^nonbZIP-inf^ (37.1% vs. 57.1%, *P* = 0.088) and *CEBPA*^wt^ (37.1% vs. 54.5%, *P* = 0.002). After a median follow-up time of 7.1 years, patients with *CEBPA*^bZIP-inf^ had significantly better OS and EFS than those with *CEBPA*^nonbZIP-inf^ (OS, median, not reached (NR) vs. 42.1 months, *P* = 0.023; EFS, median, 138.2 vs. 10.9 months, *P* = 0.002, respectively) and *CEBPA*^wt^ (OS, median, NR vs. 23.4 months, *P* < 0.0001; EFS, median, 138.2 vs. 10.4 months, *P* < 0.0001, respectively) (Fig. 1). Intriguingly, according to the WHO-2022 [15], patients with *CEBPA* mutations had significantly better EFS and a trend towards better OS than those with *CEBPA*^others^ (EFS, median, 138.2 vs. 10.4 months, *P* = 0.014; OS, median, NR vs. 23.4 months, *P* = 0.149, respectively) (Supplementary Fig. 1).

### Frequency of concurrent mutations and its prognostic relevance in *CEBPA*^bZIP-inf^

**Fig. 2:**
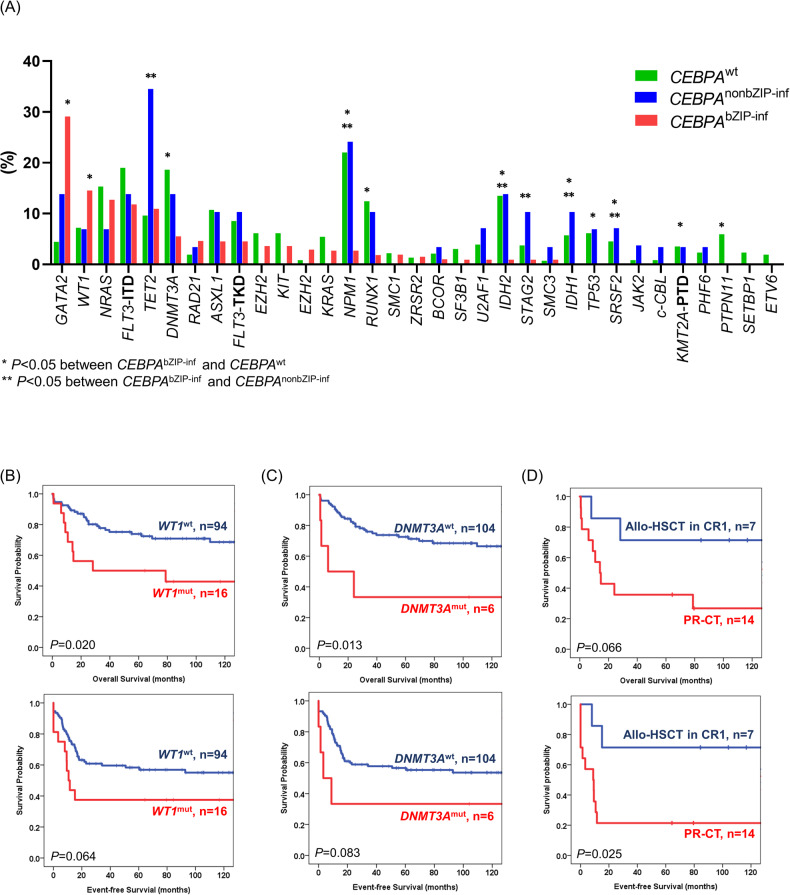
Concurrent mutations and their prognostic significance in *CEBPA*^bZIP-inf^. **A** Frequencies of concurrent mutations in patients with different *CEBPA* mutation statuses. Mutations with a frequency of at least 1% in one subgroup were shown. *P < 0.05 between *CEBPA*^bZIP-inf^ and *CEBPA*^wt^, **P < 0.05 between *CEBPA*^bZIP-inf^ and *CEBPA*^nonbZIP-inf^. **B**, **C** Concurrent *WT1* or *DNMT3A* mutations were associated with significantly shorter OS and a trend towards shorter EFS in patients with *CEBPA*^bZIP-inf^. **D** Allogeneic hematopoietic stem cell transplantation (Allo-HSCT) in first complete remission was associated with significantly longer EFS and a trend towards longer OS than post-remission chemotherapy (PR-CT) in *CEBPA*^bZIP-inf^ patients with *WT1* or *DNMT3A* mutations.

Approximately two-thirds (76 of 113, 67.3%) of patients with *CEBPA*^bZIP-inf^ had at least one concurrent mutation, and the most frequently mutated genes were *GATA2* (29.1%), *WT1* (14.5%), *NRAS* (12.7%), *FLT3*-ITD (11.8%), and *TET2* (10.9%). Patients with *CEBPA*^bZIP-inf^ had significantly higher frequencies of mutations in *GATA2* and *WT1*, but lower frequencies of mutations in *PTPN11, RUNX1, DNMT3A, IDH1, IDH2, SRSF2, NPM1, TP53*, and *KMT2A*-PTD than patients with *CEBPA*^wt^. On the other hand, *CEBPA*^bZIP-inf^ was associated with lower frequencies of mutations in *TET2, IDH1, IDH2, SRSF2, STAG2*, and *NPM1* compared with *CEBPA*^nonbZIP-inf^ (Fig. 2A and Supplementary Table 2). The results supported the different concerted mutations in biological cooperation among groups with different *CEBPA* mutational statuses.

Next, we studied the prognostic impact of concurrent mutations with the aim of risk-stratifying the patients with *CEBPA*^bZIP-inf^ more precisely. Concurrent *WT1* or *DNMT3A* mutations predicted significantly worse OS (*WT1* mutation, median, 28.2 months vs. NR, *P* = 0.020 and *DNMT3A* mutation, median, 6.1 months vs. NR, *P* = 0.013) and a trend towards worse EFS (*WT1* mutation, median, 10.6 vs. 138.2 months, *P* = 0.064 and *DNMT3A* mutation, 3.3 vs. 138.2 months, *P* = 0.083) in patients with *CEBPA*^bZIP-inf^ (Fig. 2B, C). Importantly, the outcome of *CEBPA*^bZIP-inf^ with concurrent *WT1* or *DNMT3A* mutations was similar to the ELN-2022 intermediate-risk group in terms of OS (*P* = 0.251) and EFS (*P* = 0.220). However, *GATA2*, *TET2*, ELN-2022 defined adverse gene mutations, and *FLT3*-ITD did not influence CR1, relapse rate, EFS and OS (Supplementary Fig. 2). Allogeneic hematopoietic stem cell transplantation (allo-HSCT) in CR1 was associated with significantly longer EFS and a trend towards longer OS compared with post-remission chemotherapy (PR-CT) only in *CEBPA*^bZIP-inf^ patients with *DNMT3A* or *WT1* mutations (EFS, median, NR vs. 8.9 months, *P* = 0.025; OS, median, NR vs. 14.0 months, *P* = 0.066, respectively) (Fig. 2D). For *CEBPA*^bZIP-inf^ patients without *DNMT3A* or *WT1* co-mutations, allo-HSCT in CR1 was associated with similar OS (*P* = 0.413) and EFS (*P* = 0.168) compared with PR-CT (Supplementary Fig. 3 and Supplementary Table 3). Given the limited number of cohorts with available *CEBPA*^bZIP-inf^ status, we validated our findings with datasets comprising patients with *CEBPA*^dm^. This decision was supported by evidences indicating that the majority (94.1% in the current study and 90% in the study by Taube F et al.) [4] of *CEBPA*^dm^ can be classified as *CEBPA*^bZIP-inf^. Similarly, concurrent *DNMT3A* mutation was associated with significantly poorer OS (median, 16.3 months vs. not reached, *P* = 0.029) compared with *DNMT3A* wild-type cases in the validation cohort (Supplementary Fig. 4 and Supplementary Table 4).

### Characteristics and clinical outcomes of patients with *CEBPA*^bZIP-inf^ with relapse

There were 39 patients with *CEBPA*^bZIP-inf^ who experienced a relapse. Twenty-nine (82.9%) of 35 patients who underwent re-induction chemotherapy achieved second complete remission (CR2). Among the 6 patients refractory to re-induction chemotherapy, two patients were found to have *GATA2* mutations, two patients had *WT1* mutations, two patients had cytogenetic evolution, and one patient had central nervous system involvement. Notably, the presence of *WT1* mutation was associated with a lower probability of attaining CR2 (50% vs. 92.6%, *P* = 0.031), and a trend towards inferior OS from the time of relapse (median, 2.8 vs. 26.5 months, *P* = 0.144).

The median OS from the time of relapse was 23.4 months. Twenty-one patients received subsequent allo-HSCT, including 14 patients (66.7%) in CR2. Notably, patients with allo-HSCT after relapse had significantly better OS compared with those without allo-HSCT (median, 124 vs. 13.1 months, *P* = 0.001). Furthermore, allo-HSCT in CR2 was associated with significantly better OS compared with allo-HSCT in other disease statuses (median, not reached vs. 19.9 months, *P* = 0.044) (Supplementary Fig. 5 and Supplementary Table 5).

### Comparison of transcriptional signatures between patients with *CEBPA*^bZIP-inf^ and those with *CEBPA*^nonbZIP-inf^ or *CEBPA*^wt^

**Fig. 3:**
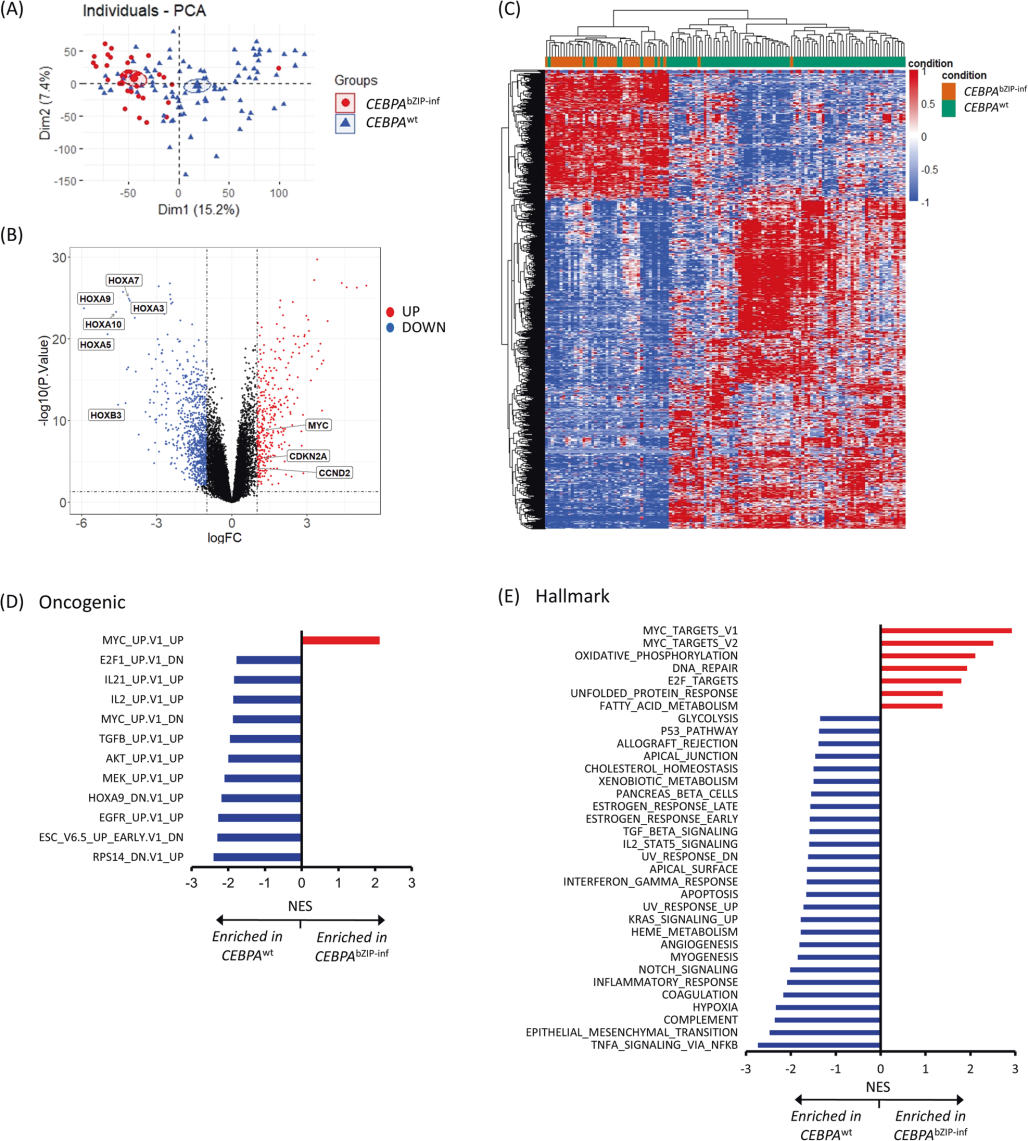
Transcriptional signatures in patients with *CEBPA*^bZIP-inf^ compared with *CEBPA*^wt^. **A** PCA showing a separation of clustering of transcriptomes between AML with *CEBPA*^bZIP-inf^ and *CEBPA*^wt^. **B** Volcano plot showing DEGs with genes downregulated (logFC<0) and upregulated (logFC>0), respectively, in patients with *CEBPA*^bZIP-inf^ compared with *CEBPA*^wt^. Multiple genes in the HOX family were downregulated in *CEBPA*^bZIP-inf^. **C** Heat map of normalized gene expression values of the top upregulated and downregulated DEGs. The DEGs were listed in Supplementary Table 6. **D**, **E** Enriched and depleted MSigDB oncogenic and Hallmark gene sets between *CEBPA*^bZIP-inf^ and *CEBPA*^wt^.

Gene expression profiles of the 36 patients with *CEBPA*^bZIP-inf^ were compared with those of the 89 patients with *CEBPA*^wt^ and normal karyotype. The principle component analysis (PCA) showed obvious separation of clustering for transcriptomes of *CEBPA*^bZIP-inf^ and *CEBPA*^wt^ AML (Fig. 3A). *CEBPA*^bZIP-inf^ was associated with 327 upregulated and 849 downregulated genes based on a |logFC|>1 cutoff and a *P* value < 0.01, with *HOXA9 (*|logFC| = 5.91), and *HOXA5 (*|logFC| = 4.97) among the highest ranked downregulated genes (Fig. 3B, C, Supplementary Table 6). By using the MsigDB Hallmark gene sets, we found that *CEBPA*^bZIP-inf^ transcriptome was enriched for MYC targets (normalized enrichment score (NES) 2.92, FDR 0), oxidative phosphorylation (NES 2.10, FDR 0), and E2F targets (NES 1.80, FDR 3.42E-04). The oncogenic gene sets revealed positive enrichment of the MYC target pathway (NES 2.12, FDR 0) and negative enrichment of genes upregulated upon knockdown of HOXA9 (NES -2.18, FDR 0) (Fig. 3D, E). As previously reported, both MYC targets and E2F targets are characterized by a proliferation signature, and dysregulation of these pathways has been witnessed in a wide variety of cancers, including myeloid neoplasms [34–36].

For validation purposes, targets of MYC and E2F were shown to be significantly enriched in the TCGA cohort when we compared the patients with *CEBPA*^bZIP-inf^ (*n* = 7) to *CEBPA*^wt^ with intermediate-risk cytogenetics (*n* = 14) (Supplementary Fig. 6A). Since there is no other public RNA-seq cohort for patients with *CEBPA*^bZIP-inf^, we performed validation in a public dataset of patients with *CEBPA*^dm^. In GSE15210, a total of 83 genes were differentially expressed between *CEBPA*^dm^ and *CEBPA*^wt^ patients [10]. Fifty-one (83.6%) out of 61 downregulated genes and 22 (100%) of 22 upregulated genes were also significantly differentially expressed in our RNA-seq data when comparing the transcriptomes between patients with *CEBPA*^dm^ and *CEBPA*^wt^. The major downregulated genes belonged to the homeobox gene family (*HOXA9, HOXA10*, *HOXA5, HOXA3, HOXA6*, etc). The results confirmed the concordance of our RNA-seq data with others (Supplementary Fig. 6B).

### Association of dysregulated immune and metabolic pathways with short EFS in patients with *CEBPA*^bZIP-inf^

**Fig. 4:**
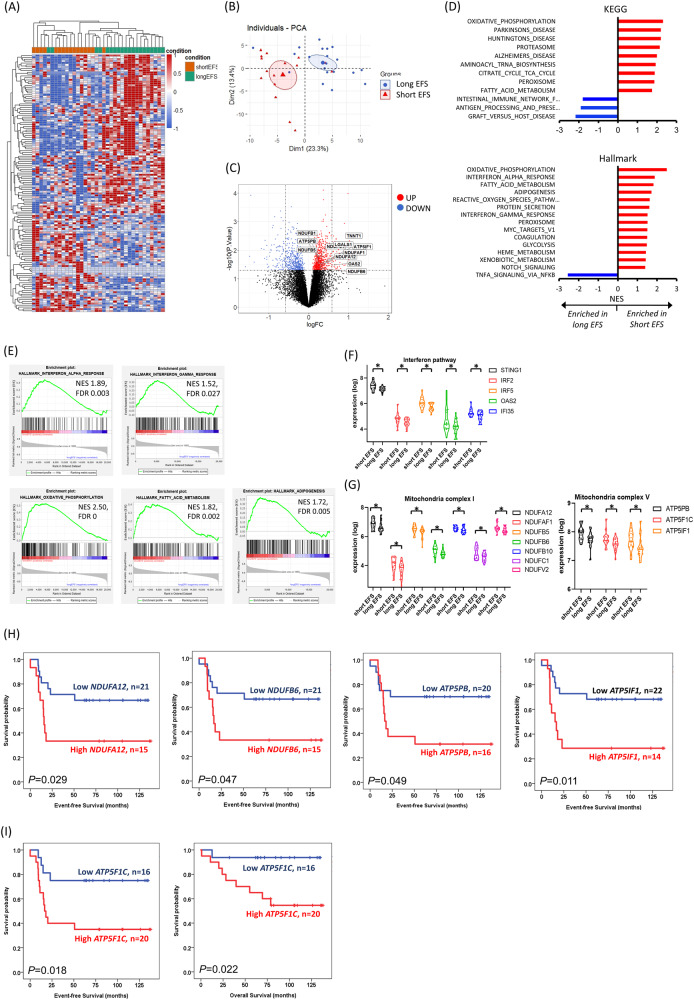
Differential transcriptional signatures between *CEBPA*^bZIP-inf^ with short and long EFS. **A** Heat map of normalized gene expression values of the top upregulated and downregulated DEGs in patients with short EFS. The DEGs were listed in Supplementary Table 7. **B** PCA showing separate clustering of transcriptomes between *CEBPA*^bZIP-inf^ AML with short and long EFS based on the top DEGs. **C** Volcano plot showing DEGs with genes downregulated (logFC<0) and upregulated (logFC>0), respectively, in *CEBPA*^bZIP-inf^ patients with short EFS compared with long EFS. Important genes in the metabolic and immune pathways are highlighted. **D** Enriched and depleted MSigDB Hallmark and KEGG gene sets between patients with short and long EFS. **E** GSEA enrichment plots showing overrepresentation of IFN response and metabolic gene set signatures within the *CEBPA*^bZIP-inf^ transcriptome. **F**, **G** Scatter plots illustrating the log2-transformed, CPM-normalized expression values in samples associated with short *vs*. long EFS for genes involved in IFN response (**F**) and oxidative phosphorylation (**G**). *P value < 0.05. **H** Kaplan–Meier plots for EFS for patients with low expression (blue lines) and high expression (red lines) of *NDUFA12, NDUFB6, ATP5PB, ATP5IF1* at diagnosis. The mean expression value for the respective gene in all *CEBPA*^bZIP-inf^ samples was used to discretize between low and high expression. **I** High expression of *ATP5F1C* was associated with significantly shorter OS and EFS in patients with *CEBPA*^bZIP-inf^.

Among the 39 patients with *CEBPA*^bZIP-inf^ who had relapsed, only 8 (20.5%) had *WT1* or *DNMT3A* mutations. Since concurrent mutations could only partly explain the poor clinical outcomes in *CEBPA*^bZIP-inf^, we hypothesized that, in addition to mutations, transcriptomic variation underpins a large proportion of functional variation. To elucidate why some patients with *CEBPA*^bZIP-inf^ still had poor clinical outcomes at the transcriptomic level, we compared the RNA-seq data between *CEBPA*^bZIP-inf^ patients with short EFS (*n* = 16, EFS < 2 years) and long EFS (*n* = 20). There were 138 DEGs with |logFC|>0.585 (absolute fold change >1.5 or <0.67) and *P* value < 0.05, which could separate the patients well on unsupervised clustering and PCA (Fig. 4A–C and Supplementary Table 7). This finding highlighted the possibility that some genes with lower fold changes were also important determinants of clinical outcomes. The MsigDB Hallmark gene sets showed significant enrichment of immune and metabolic pathways in *CEBPA*^bZIP-inf^ with short EFS. The enriched immune pathways include IFN alpha and gamma response (NES 1.89, FDR 0.003; NES 1.52, FDR 0.027, respectively). The Reactome gene sets and Gene Ontology on biological process revealed an enrichment of IFN alpha signaling and type I IFN response (Fig. 4D, E and Supplementary Fig. 7A). *STING1, IRF2, IRF5, OAS2, IFI35*, and *FADD* were significantly overexpressed in patients with short EFS (Fig. 4F). Higher expression of *STING1, IRF5, OAS2, IFI35*, and *FADD* were associated with a trend towards shorter EFS in *CEBPA*^bZIP-inf^ (Supplementary Fig. 8). To validate the results in an independent cohort, RNA-seq data from 20 *CEBPA*-mutated patients in the TARGET AML cohort were analyzed. Similarly, gene sets related to response to IFN were significantly enriched in those with shorter EFS (Supplementary Fig. 7B).

The enriched metabolic pathways included oxidative phosphorylation (NES 2.50, FDR 0), fatty acid metabolism (NES 1.82, FDR 0.002), and adipogenesis (NES 1.72, FDR 0.005). The KEGG gene sets also revealed perturbed pathways involving oxidative phosphorylation (NES 2.32, FDR 0) and the proteasome (NES 2.15, FDR 0) (Fig. 4D, E). Molecules in mitochondrial complex I (*NDUFB10, NDUFC1, NDUFV2, NDUFAF1, NDUFB5, NDUFA12* and *NDUFB6*) and complex V (*ATP5PB, ATP5F1C* and *ATP5IF1*) were significantly overexpressed in patients with short EFS (Fig. 4G). Higher expression of *NDUFA12* (*P* = 0.029), *NDUFB6* (*P* = 0.047), *ATP5PB* (*P* = 0.049), and *ATP5IF1* (*P* = 0.011) was associated with significantly shorter EFS in *CEBPA*^bZIP-inf^, and higher expression of *ATP5F1C* was associated with both significantly shorter OS and EFS (*P* = 0.022, and *P* = 0.018, respectively) (Fig. 4H, I). Next, we investigated whether there are different clinic-biological features between those with high or low expression of *ATP5F1C*, but found no discernible difference with regard to karyotypic changes or mutations (Supplementary Table 8). The prognostic impact of mitochondrial complexes was validated in the TARGET AML cohort, which demonstrated that higher expression of *NDUFA12* (*P* = 0.076) and *NDUFB6* (*P* = 0.013) also correlated with shorter EFS in *CEBPA*-mutated patients (Supplementary Fig. 9).

We additionally studied the genetic and transcriptomic heterogeneity within the *CEBPA*^nonbZIP-inf^ group (*n* = 11). Concurrent *GATA2* mutation was associated with a trend towards longer EFS, while *TET2* mutation was associated with a trend towards shorter OS, albeit the small number of patients. There were 822 DEGs between *CEBPA*^nonbZIP-inf^ patients with short and long EFS, of which 24 coincided with the DEGs that differentiated the outcomes in patients with *CEBPA*^bZIP-inf^. The detailed enriched pathways on GSEA were shown in Supplementary Fig. 10.

To address the question whether enrichment of interferon signaling and metabolic pathways were associated with shorter EFS in other AML subtypes, we analyzed RNA-seq data from 60 AML patients with *NPM1* mutation and compared the transcriptomes between patients with short EFS (EFS < 2 years, *n* = 34) and those with long EFS (*n* = 26). Those with short EFS did not have a significant enrichment of metabolic pathways, such as oxidative phosphorylation (NES −1.06, FDR 0.35) and fatty acid metabolism (NES 0.67, FDR 0.97). However, we observed a negative enrichment of interferon signaling (NES -3.34, FDR 0) in those with short EFS (Supplementary Fig. 11). Although enriched interferon signaling pathways have previously been associated with unfavorable prognostic implications in the overall AML cohort [37], our findings suggested that the enriched pathways are context-dependent and differ across various AML subtypes.

## Discussion

The current study leverages a large cohort of AML to study the genomic and transcriptomic pattern that contribute to poor survival in AML with *CEBPA*^bZIP-inf^, a new disease category in the ELN-2022 and 2022 ICC. We identified *WT1* and *DNMT3A* mutations, dysregulated immune and metabolic signatures that highly correlated with poor survival. We have generated a publicly available database for patients with *CEBPA*^bZIP-inf^ that contains patient demographics, clinical survival data, RNA-seq, and NGS data, which serves as a resource for the AML research community.

The reports on the prognostic impact of concurrent mutations *in CEBPA*^bZIP-inf^ have been re limited to *GATA2*, *WT1*, and *TET2* [4, 18]. Mutant *GATA2* is associated with improved survival, while *WT1* and *TET2* mutations are associated with a lower probability of survival [4]. In our study, concurrent *WT1* or *DNMT3A* mutations were associated with significantly poorer survival. We are the first to report the prognostic relevance of *DNMT3A* mutations in *CEBPA*^bZIP-inf^, and the result has been validated in external databases. However, due to the small number of *DNMT3A* co-mutant patients in both our cohort and the external databases, further investigation in a larger independent cohort is still warranted to validate these findings. Recently, it was discovered that CEBPA and DNMT3A cooperate to induce leukemogenesis. Mutant CEBPA interacts with the long splice isoform DNMT3A and increase the aberrant methylation especially on PRC2 target genes [38]. We additionally found that *CEBPA*^bZIP-inf^ patients with *WT1* or *DNMT3A* mutations had improved survival if receiving allo-HSCT in CR1. In addition, *WT1-*mutated patients had a lower probability of attaining CR2 and displayed a trend towards inferior OS following relapse. These results highlight the importance of utilizing a personalized treatment approach in determining the appropriate timing of allo-HSCT within the ELN-2022 favorable category.

Since *WT1* or *DNMT3A* mutations are presented in only 20.5% patients with *CEBPA*^bZIP-inf^ who had relapsed, it’s apparent that non-genetic drivers underpin a large proportion of functional variation in leukemia. Indeed, the RNA-seq revealed enrichment of IFN and metabolic pathways in those with short EFS. Cumulative evidences have supported the role of immune microenvironment in AML, and the activation of IFN-related pathways has been shown to have negative prognostic implications in AML [37, 39]. Chronic activation of the IFN signaling pathway actually creates an immune-suppressed environment and mediates resistance to various cancer treatments [40]. In our study, enriched IFN-related pathways also correlated with a poorer survival. Patients with short EFS and long EFS expressed comparable amounts of leukemia-associated antigens CD34, CD123, and CD117 (Supplementary Fig. 12). This observation suggests that bulk BM RNA-seq could capture elements of the tumor immune microenvironment in addition to features of the leukemic cell compartment.

Another notable discovery is the dysregulated metabolism in those with short EFS. The results corroborate the findings that aberrant metabolic states are hallmarks of various types of cancer [41]. Oxidative phosphorylation, involving mitochondria complexes I-V, has been reported to play a role in leukemia stem cell maintenance in myeloid leukemia [42, 43]. Genes in the mitochondrial complexes I and V were most prominently dysregulated and also correlated with survival in the TARGET AML cohort. It’s noteworthy that many genes in the mitochondrial complexes had fold change less than 2, suggesting biologically relevant functions occur even at very low fold changes in RNA levels [44].

The use of bulk BM aspirates is a potential limitation of our analysis. Single-cell RNA-seq or mass cytometry will further elucidate which cell component makes the major contribution. The number of patients with *CEBPA* mutations was not adequate in the TARGET AML cohort, which potentially decreased the reliability of the external validation. Besides, we did not perform mechanistic studies to elucidate the detailed molecular pathways in *CEBPA*^bZIP-inf^. Further studies are needed to investigate whether targeting of these pathways could sensitize the leukemic cells and ultimately reduce relapse in these patients.

In conclusion, our results highlight the importance of the combination of genetic and transcriptomic approaches to fully elucidate the heterogeneities within a WHO-2022/ICC-defined entity. We identified *WT1* mutations, *DNMT3A* mutations, dysregulated interferon and oxidative phosphorylation as negative prognosticators in patients with *CEBPA*^bZIP-inf^. We provide a cohort with complete patient demographics, clinical survival, NGS, and RNA-seq data that will nourish further research in the AML community.

### Supplementary information

Supplementary Data

Supplementary table 6

## Data Availability

RNA-seq data can be accessed at NCBI GEO under GSE253086. Clinical and mutation data and computer codes with nonidentifiable patient information are accessible on reasonable request. Evaluation of such requests will be conducted by the corresponding author (H.-A.H.) to determine their appropriateness.
