# Supplementary material for: Dysregulated immune and metabolic pathways are associated with poor survival in adult acute myeloid leukemia with CEBPA bZIP in-frame mutations
Source: Blood Cancer J. 2024 Jan 23;14(1):15. doi: 10.1038/s41408-023-00975-8 (PMC10803338; doi:10.1038/s41408-023-00975-8)
Supplement: Supplementary file 1 — Supplementary Data [file 41408_2023_975_MOESM1_ESM.docx]

**Supplementary Material**

**Dysregulated Immune and Metabolic Pathways were associated with Poor Survival in Adult Acute Myeloid Leukemia with *CEBPA* bZIP in-frame Mutations**

Feng-Ming Tien,^1,2^ Chi-Yuan Yao,^1,2,3^ Cheng-Hong Tsai,^1^ Min-Yen Lo,^2,4^ Chien-Yuan Chen,^1^ Wan-Hsuan Lee,^2,5^ Chien-Chin Lin,^1,3^ Yuan-Yeh Kuo,^6^ Yen-Ling Peng,^1^ Mei-Hsuan Tseng,^6^ Yu-Sin Wu,^7^ Ming-Chih Liu,^8^ Liang-In Lin,^9^ [Ming-Kai Chuang](https://pubmed.ncbi.nlm.nih.gov/?sort=date&term=Chuang+MK&cauthor_id=32275807),^1,3^Bor-Sheng Ko,^1,10^ Ming Yao,^1^ Jih-Luh Tang,^1,6,10^ Wen-Chien Chou,^1,3^ Hsin-An Hou,^1^ Hwei-Fang Tien^1,11^

**Affiliations:**

^1^Division of Hematology, Department of Internal Medicine, National Taiwan University Hospital, Taipei, Taiwan

^2^Graduate Institute of Clinical Medicine, College of Medicine, National Taiwan University, Taipei, Taiwan

Departments of ^3^Laboratory Medicine, and ^8^Pathology, National Taiwan University Hospital, Taipei, Taiwan

^4^Division of Hematology, Department of Internal Medicine, National Taiwan University Hospital Yunlin Branch, Yunlin, Taiwan.

^5^Department of Internal Medicine, National Taiwan University Hospital, Hsin-Chu Branch, Hsin-Chu, Taiwan

^6^Tai-Chen Cell Therapy Center, National Taiwan University, Taipei, Taiwan

Departments of ^7^Nursing, and ^10^Hematological Oncology, National Taiwan University Cancer Center, Taipei, Taiwan

^9^Department of Clinical Laboratory Sciences and Medical Biotechnology, College of Medicine, National Taiwan University, Taipei, Taiwan

^11^Department of Internal Medicine, Far-Eastern Memorial Hospital, New Taipei City, Taiwan.

**Supplementary Methods ………………………………………………………..…………P.1**

**Supplementary Figures ……………………………………………………………………P.2**

**Supplementary Tables ……………………………………………………………………..P.16**

**Supplementary Methods**

**Subjects**

We enrolled 887 newly diagnosed patients with *de novo* AML at the National Taiwan University Hospital (NTUH). To focus on a more homogeneous group of patients with AML, those with antecedent hematological diseases, history of cytopenia, family history of myeloid neoplasms or therapy-related AML were excluded. All patients received standard induction chemotherapy and consolidation. The consolidation chemotherapy was 2-4 courses of high-dose cytarabine (2000 mg/m^2^ every 12 hours for days 1-4, total 8 doses), with or without one anthracycline idarubicin (12 mg/m^2^) or mitoxantrone (12 mg/m^2^). Among the 379 patients who underwent allogeneic hematopoietic stem cell transplantation (allo-HSCT), 182 (48.0%) received allo-HSCT in CR1. The choice of allo-HSCT for patients afflicted with AML at our institute is largely based on chromosomal results, mutation data, age, availability of HLA-matched donors as well as the response to induction treatment.

In this study, 26 patients with *CEBPA*^bZIP-inf^ underwent allo-HSCT in CR1. Among them, 12 had hyperleukocytosis at diagnosis, 9 with delayed CR1, 3 with *FLT3-*ITD, 1 with adverse cytogenetics and 4 with persistent measurable residual disease as indicated by flow cytometry following consolidation chemotherapy. The detailed characteristics of these patients were illustrated in Supplementary Table 3.

**Supplementary Figures**

**Supplementary Figure 1**

**OS and EFS stratified by *CEBPA* statuses according to the 2022 WHO classification**

AML with *CEBPA* mutation was defined as biallelic *CEBPA* and single mutation in bZIP in the 2022 WHO classification.

**(A)**


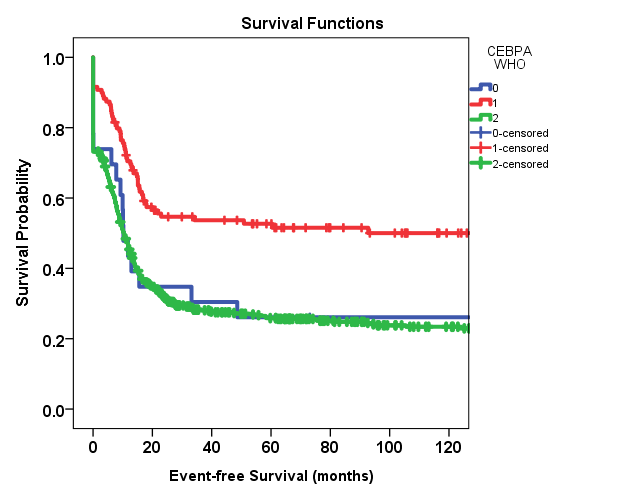

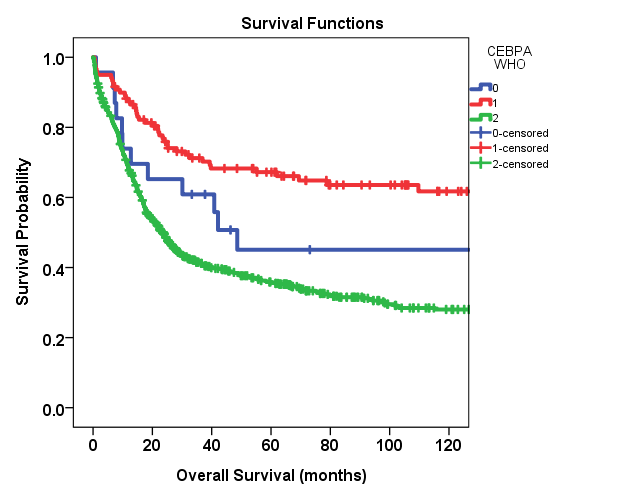


***CEBPA* mutation, n=119**

***CEBPA*^others^, n=23**

***CEBPA*^wt^, n=745**

***CEBPA* mutation, n=119**

***CEBPA*^others^, n=23**

***CEBPA*^wt^, n=745**

|  | *CEBPA*^wt^ | *CEBPA*^others^ |  |  | *CEBPA*^wt^ | *CEBPA*^others^ |
| --- | --- | --- | --- | --- | --- | --- |
| *CEBPA*^others^ | 0.124 | - |  | *CEBPA*^others^ | 0.749 | - |
| *CEBPA* mutations | <0.0001 | 0.149 |  | *CEBPA* mutations | <0.0001 | 0.014 |

**Supplementary Figure 2**

**OS and EFS stratified by concurrent mutations in patients with *CEBPA*^bZIP-inf^**

Presence of *FLT3*-ITD (A), *GATA2* (B), *TET2* (C), ELN-2022 defined adverse mutations (*ASXL1, BCOR, EZH2, RUNX1, SF3B1, SRSF2, STAG2, U2AF1* and *ZRSR2*) did not significantly affect the survival outcomes in patients with *CEBPA*^bZIP-inf^.

**(A)**

**
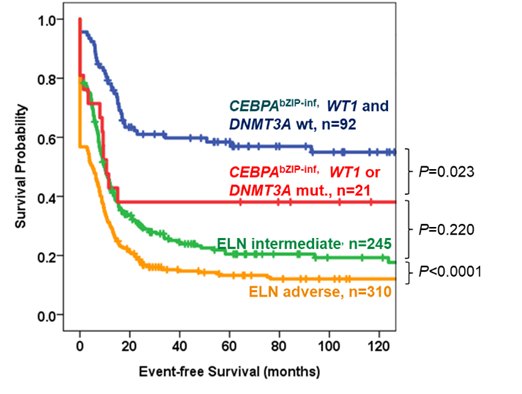
**
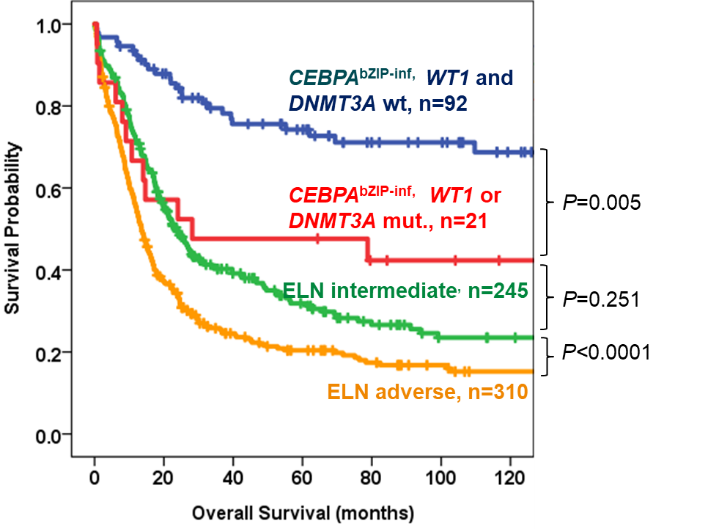


**(B)**


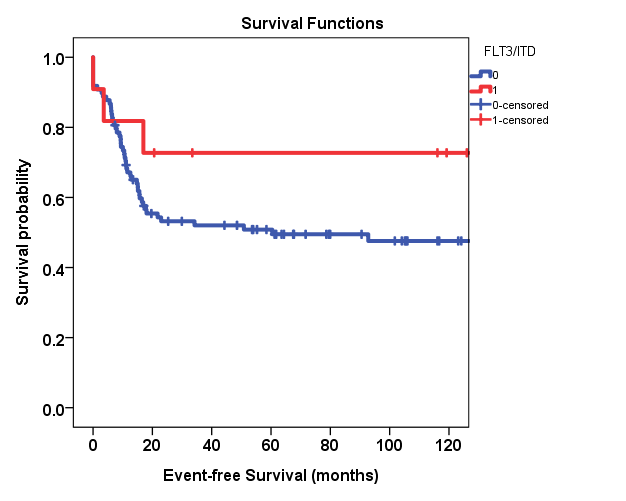

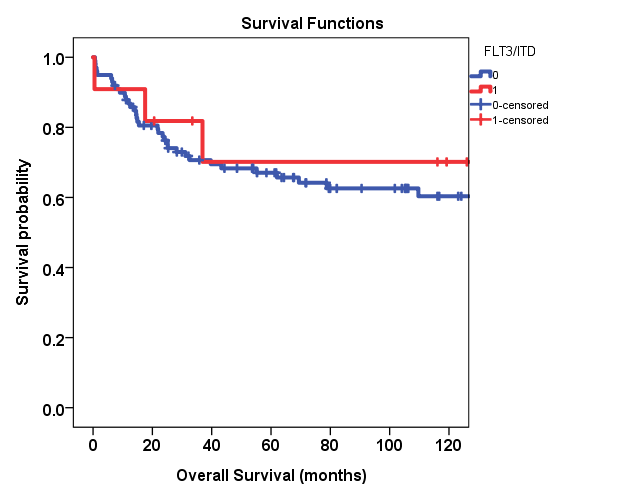


***FLT3-*ITD^mut^, n=11**

***FLT3*-ITD^wt^, n=99**

*P*=0.519

***FLT3-*ITD^mut^, n=11**

***FLT3-*ITD^wt^, n=99**

*P*=0.168

**(C)**


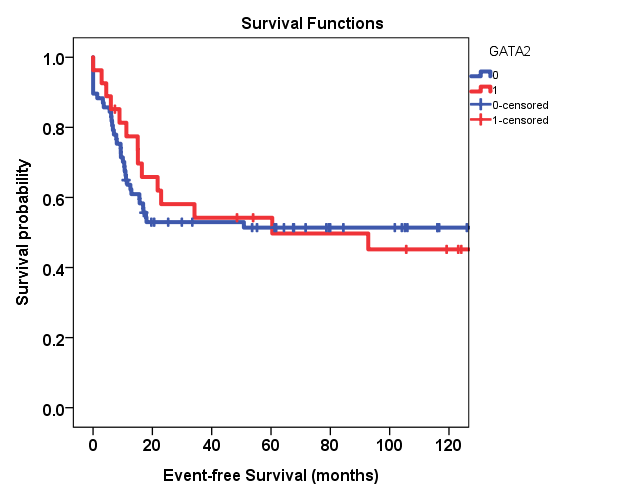

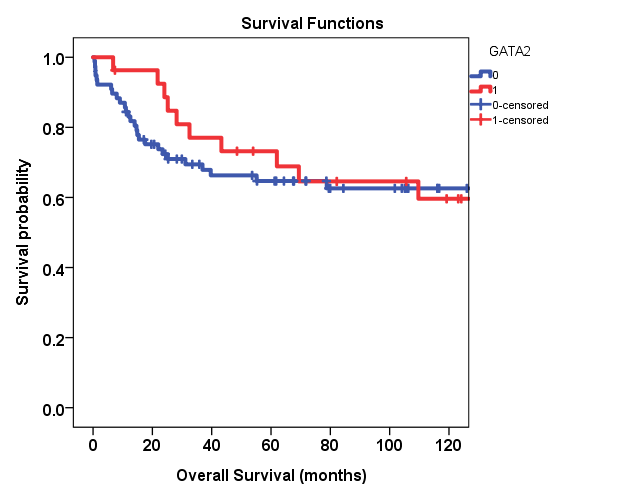


***GATA2*^mut^, n=27**

***GATA2*^wt^, n=77**

*P*=0.912

***GATA2*^mut^, n=27**

***GATA2*^wt^, n=77**

*P*=0.587

**(D)**


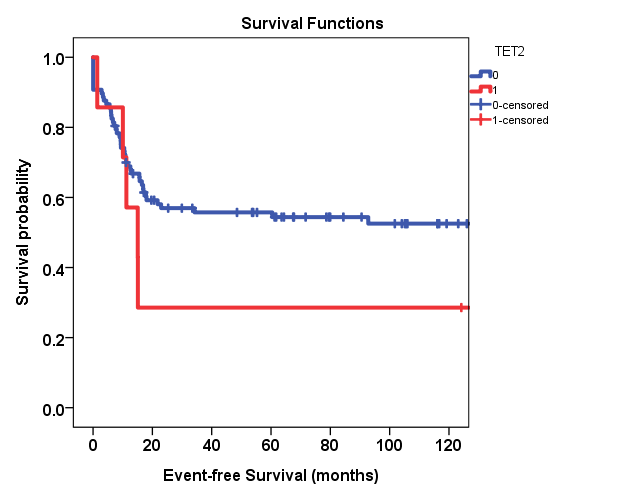

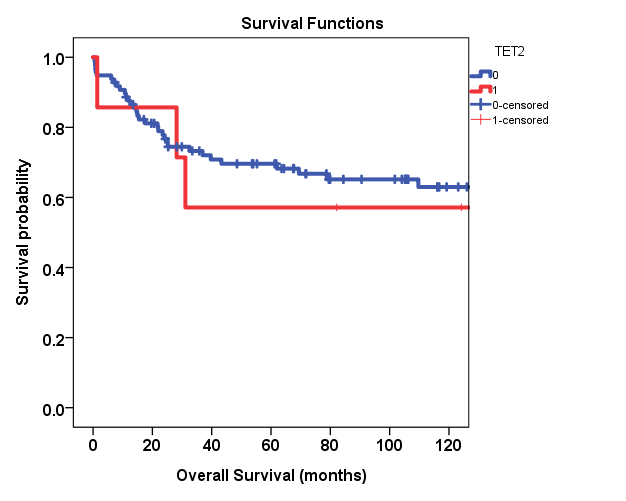


***TET2*^mut^, n=7**

***TET2*^wt^, n=97**

*P*=0.830

***TET2*^mut^, n=7**

***TET2*^wt^, n=97**

*P*=0.214

**(E)**


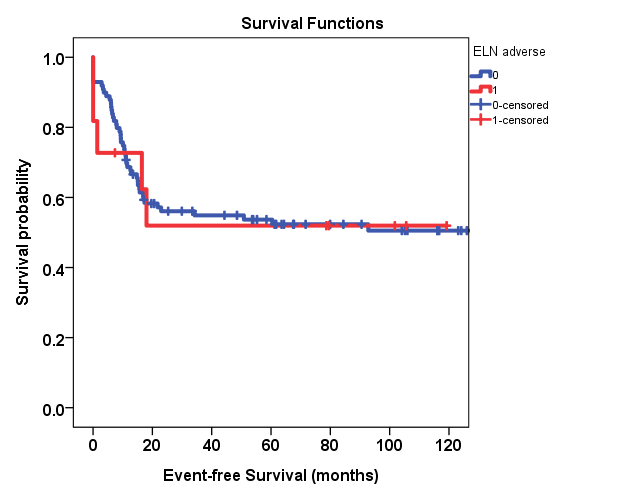

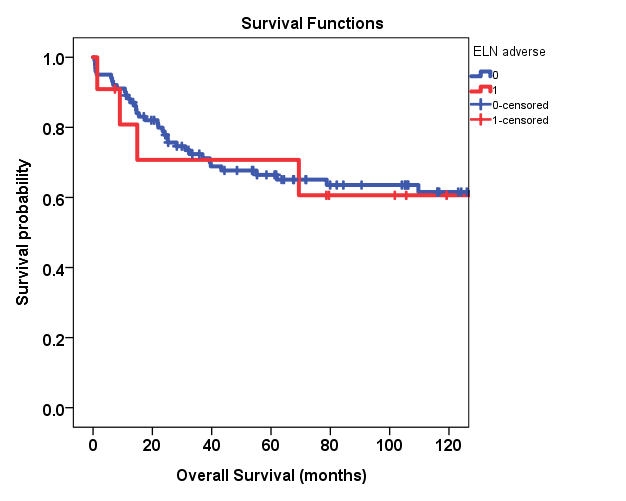


**Others, n=101**

**ELN-adverse mutations, n=11**

*P*=0.919

**Others, n=101**

**ELN-adverse mutations, n=11**

*P*=0.865

**Supplementary Figure 3**

**Transplantation outcomes in *CEBPA*^bZIP-inf^ patients without *WT1* or *DNMT3A* mutations**

Allogeneic hematopoietic stem cell transplantation (Allo-HSCT) in first complete remission was associated with similar OS (*P*=0.413) and EFS (*P*=0.168) compared with post-remission chemotherapy (PR-CT) in *CEBPA*^bZIP-inf^ patients without *WT1* or *DNMT3A* mutations.

**
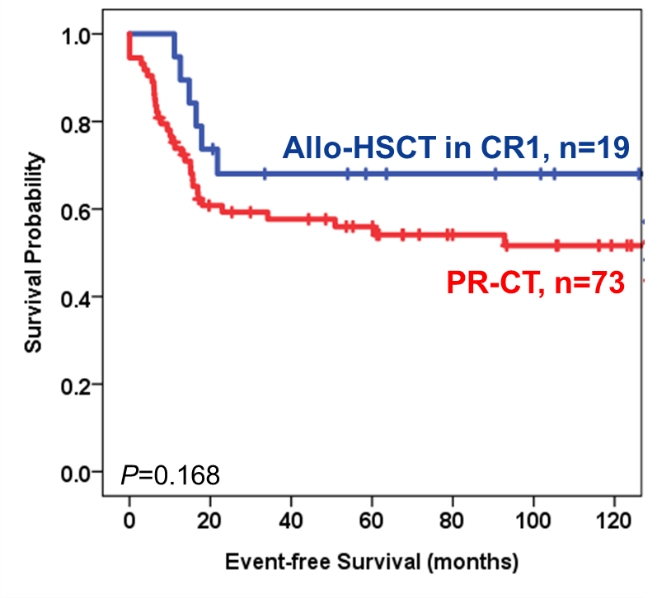
**

**
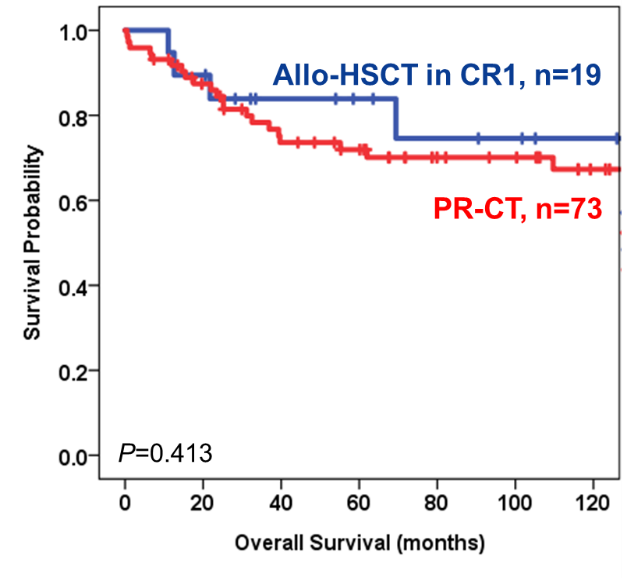
**

**Supplementary Figure 4**

**Prognostic impact of *DNMT3A* mutation in AML patients with *CEBPA*^dm^ in the validation cohorts**

A total of 150 patients with *CEBPA*^dm^ were identified from four external cohorts, including 73 patients from the UK-NCRI trials, 51 patients from the AMLSG cohort, 4 patients from the TCGA cohort, and 22 patients from NTUH Yunlin Branch cohort. The characteristics of these patients were depicted in Supplementary Table 4. Concurrent *DNMT3A* mutation was associated with significantly poorer OS compared with *DNMT3A* wild-type cases (median, 16.3 months *vs.* not reached, *P*=0.029).


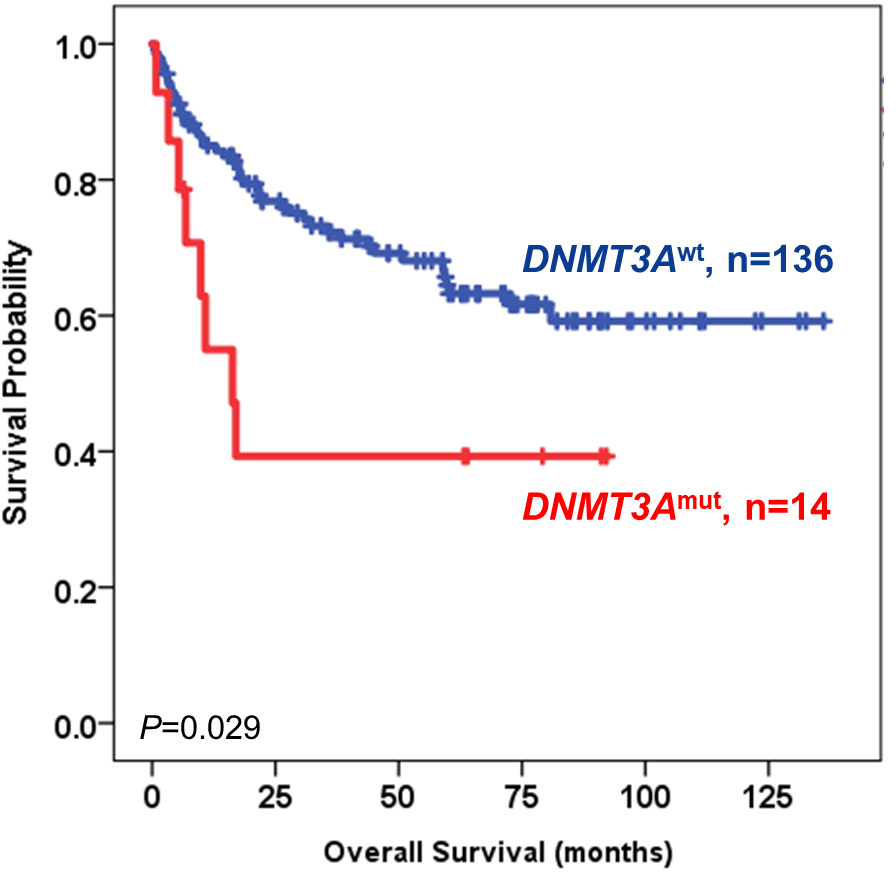


**Supplementary Figure 5**

**Clinical outcomes of patients with *CEBPA*^bZIP-inf^ who have experienced relapse**

(A) Presence of *WT1* mutation was associated with a trend towards inferior OS from the time of relapse (median, 2.8 *vs.* 26.5 months, *P*=0.144). (B) Patients with allo-HSCT after relapse had significantly better OS compared with those without allo-HSCT (median, 124 *vs.* 13.1 months, *P=*0.001). (C) Allo-HSCT in CR2 was associated with significantly better OS compared with allo-HSCT in other disease statuses (median, not reached *vs.* 19.9 months, *P*=0.044).

**(A)
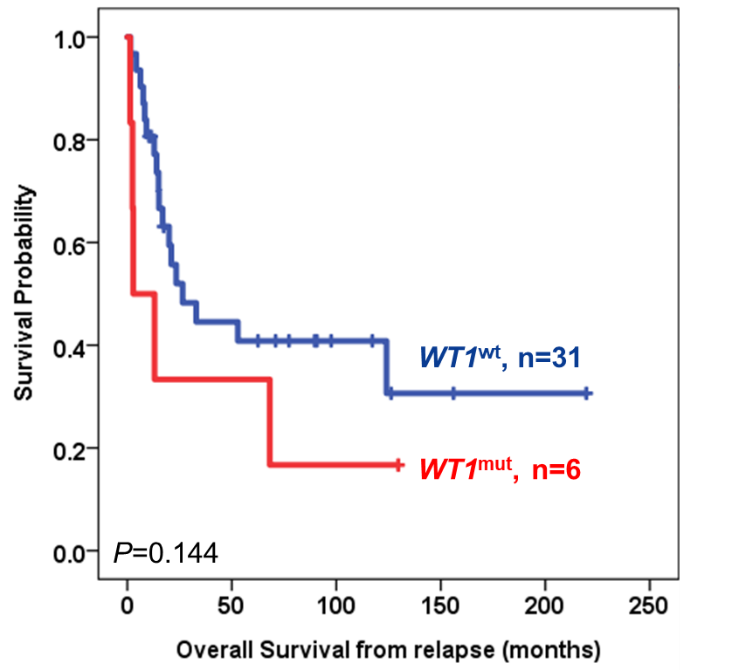
**

**(B) (C)**

**
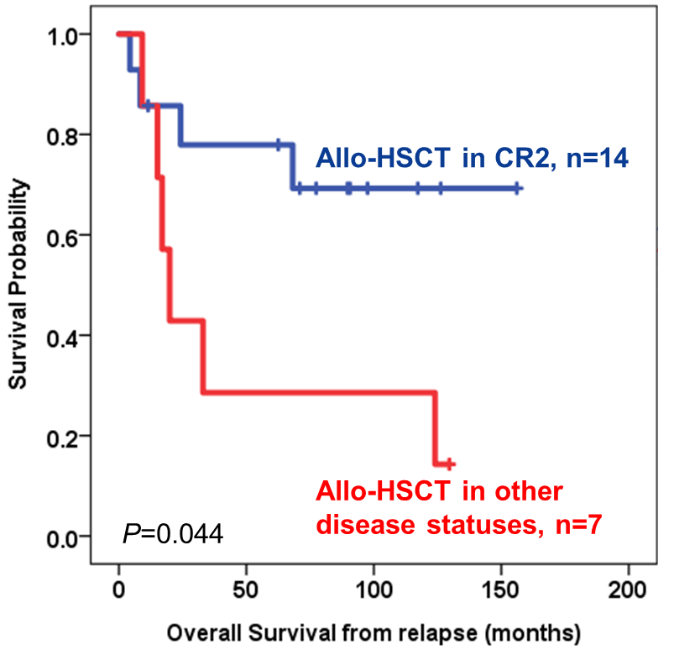

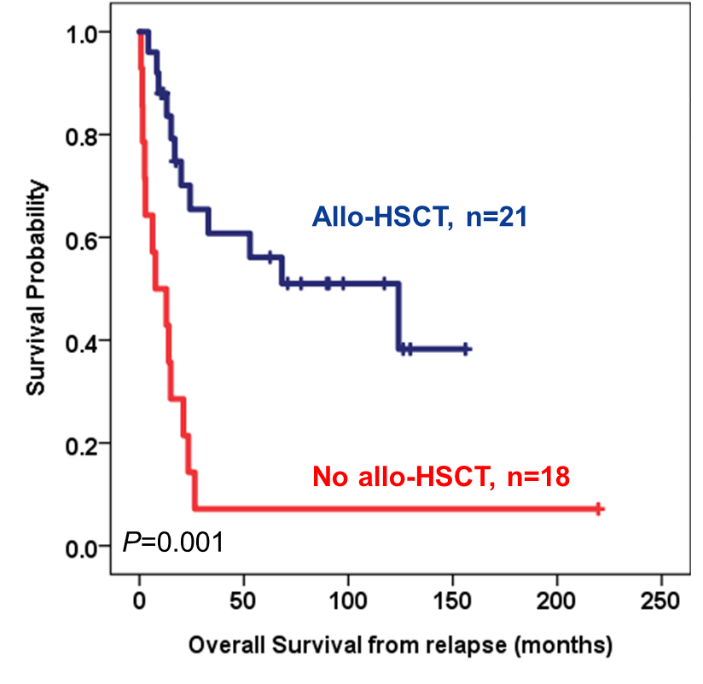
**

**Supplementary Figure 6**

**Validation of RNA-seq data with external data sets**

(A) MYC targets and E2F targets were significantly enriched in the TCGA cohort (B) Venn diagram of differentially expressed genes (DEGs) between *CEBPA*^dm^ and *CEBPA*^wt^ in GSE15210 (green) and our study (orange). In GSE15210 and our study, the DEGs are selected based on a fold change of more than two-fold and a *P* value of less than 0.01.

**(A)**

**
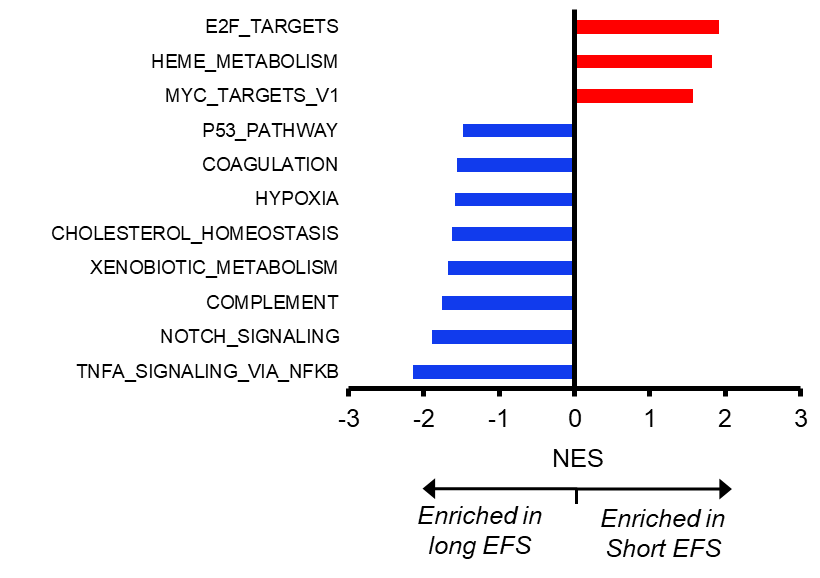
**

**(B)**

**
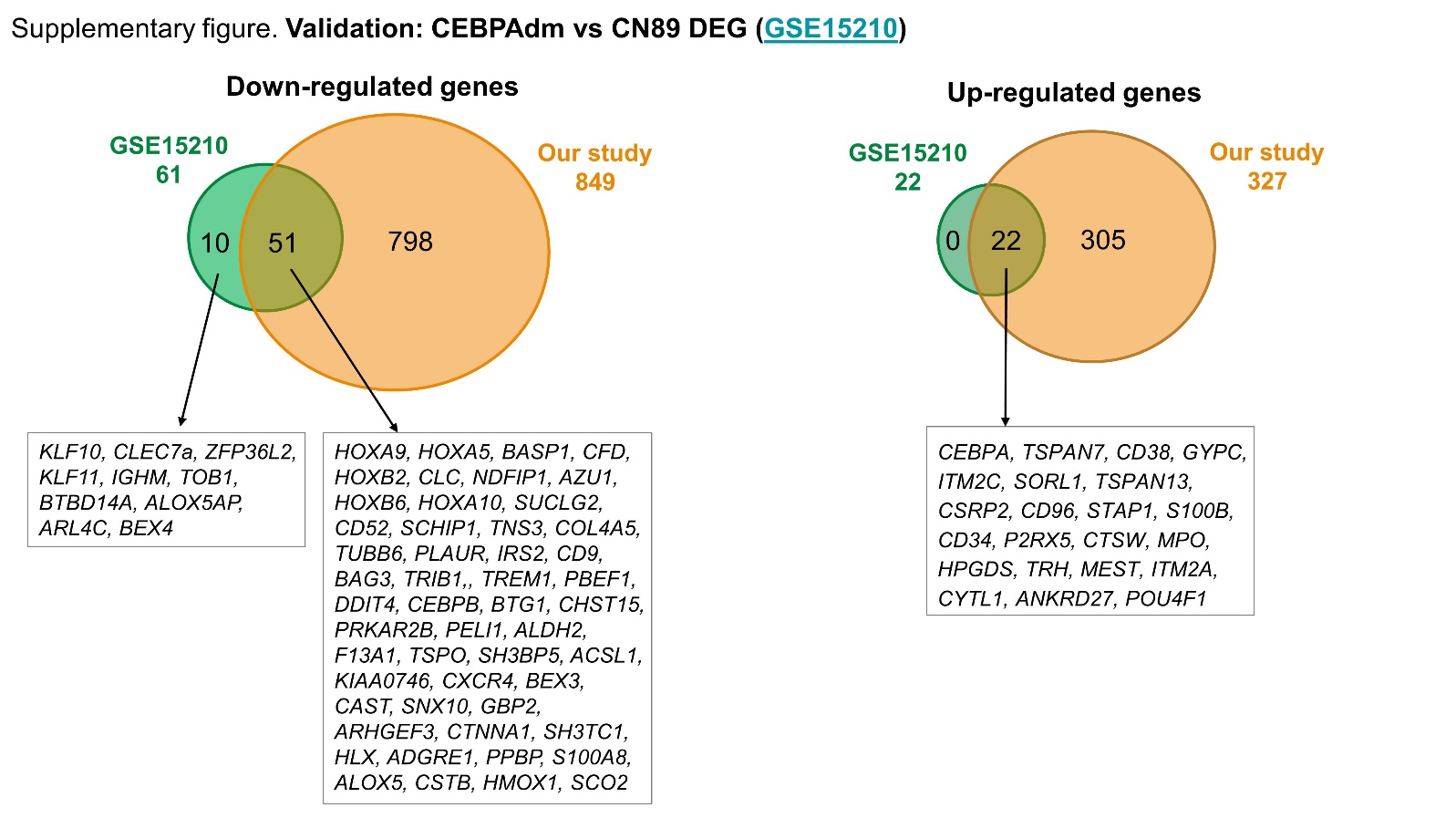
**

**Supplementary Figure 7**

**Enrichment of immune related gene sets in patients with short EFS**

(A) In our study, the Reactome gene sets and Gene Ontology on biological process revealed an enrichment of IFN alpha signaling and type I IFN response in patients with *CEBPA*^bZIP-inf^ with short EFS. (B) In the TARGET AML cohort, gene sets related to response to IFN were significantly enriched in patients with mutated *CEBPA* with short EFS.

**(A)**

**
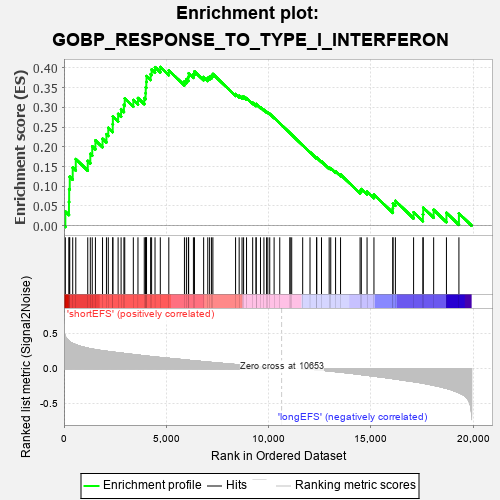

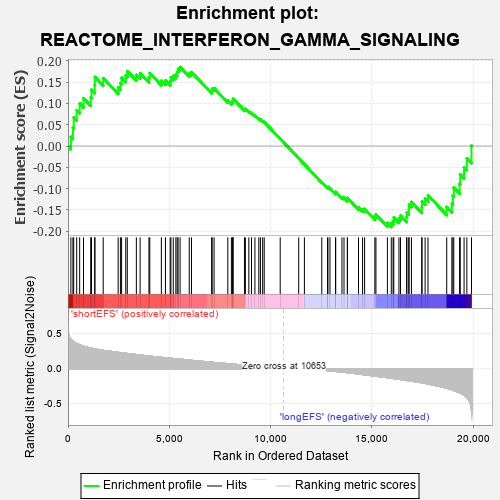

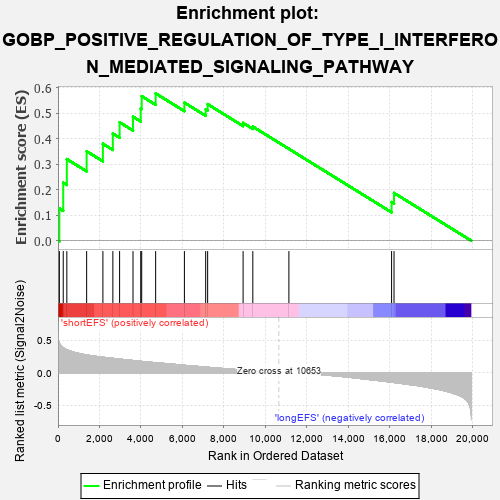
**

**NES 1.75, FDR 0.016**

**NES 1.65, FDR 0.0003**

**NES 1.65, FDR 0.019**

**(B)**

**
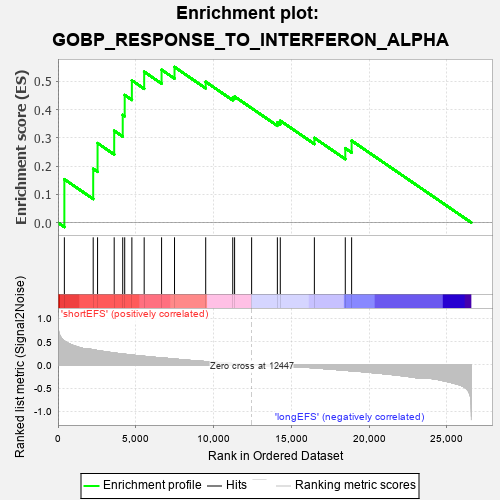

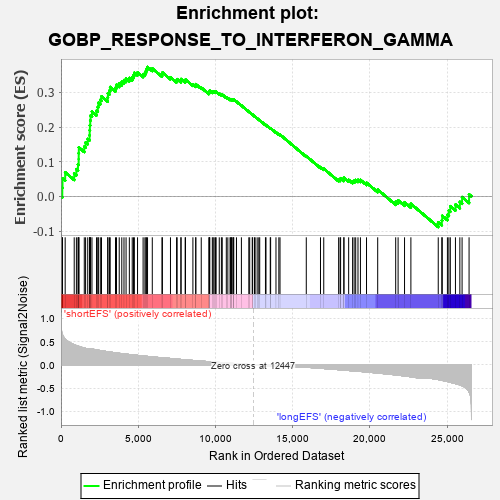
**

**NES 1.71, FDR 0.014**

**NES 1.73, FDR 0.019**

**Supplementary Figure 8**

**Prognostic significance of immune related genes in patients with *CEBPA*^bZIP-inf^ with short EFS**

Kaplan-Meier plots for EFS for patients with low expression (blue lines) and high expression (red lines) of *STING1, IRF5, OAS2, IFI35*, and *FADD* at diagnosis. The mean expression value for the respective gene in all *CEBPA*^bZIP-inf^ samples was used to discretize between low and high expression.

**
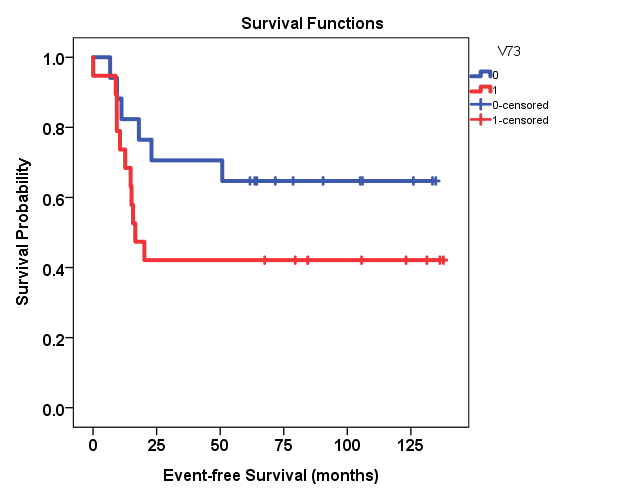
**
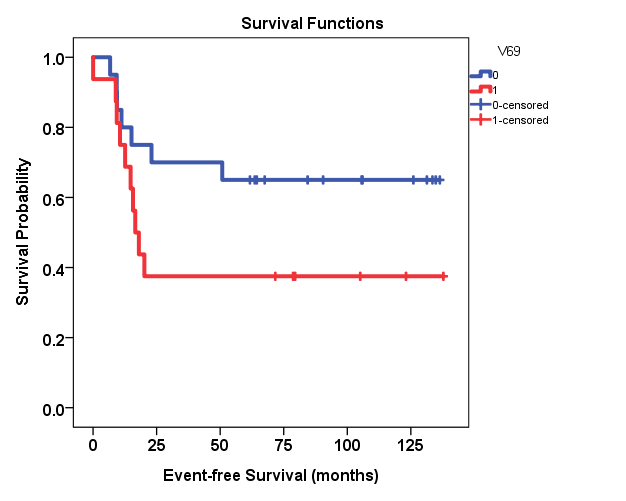


**High *IRF5*, n=19**

*P*=0.135

**Low *IRF5*, n=17**

**High *STING1*, n=16**

*P*=0.096

**Low *STING1*, n=20**

**
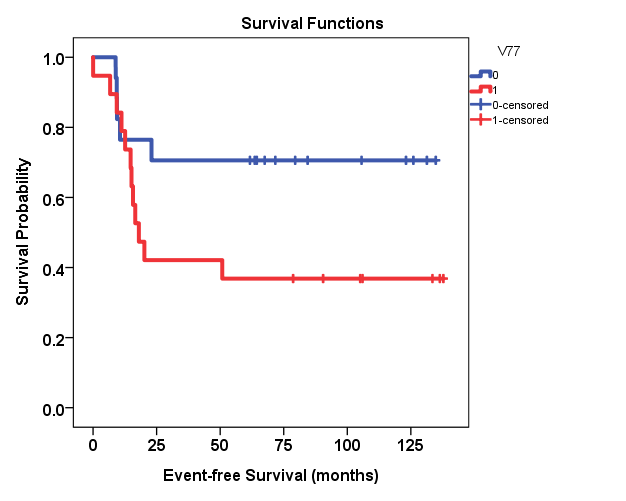

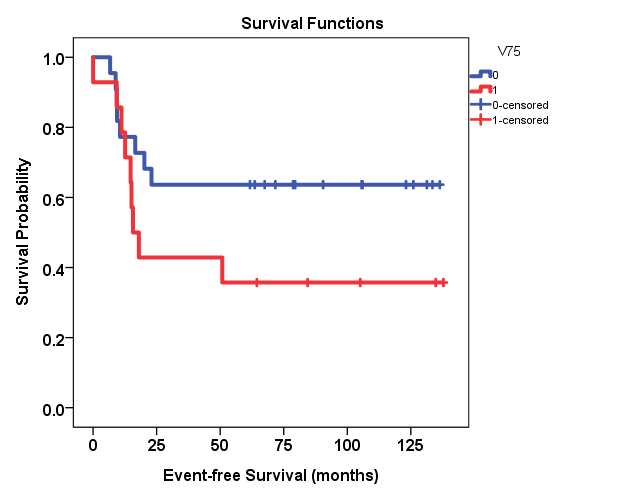
**

**High *IFI35*, n=19**

*P*=0.070

**Low *IFI35*, n=17**

**High *OAS2*, n=14**

*P*=0.128

**Low *OAS2*, n=22**

**
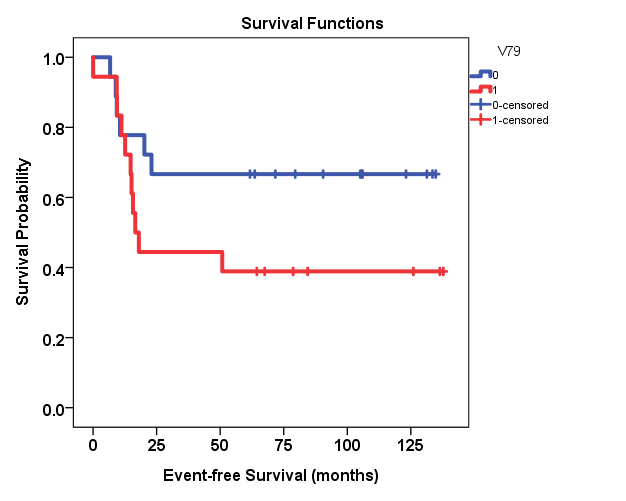
**

**High *FADD*, n=18**

*P*=0.127

**Low *FADD*, n=18**

**Supplementary Figure 9**

**Validation of the prognostic significance of mitochondrial complexes in the TARGET AML cohort**

Kaplan-Meier plots for EFS for patients with low expression (blue lines) and high expression (red lines) of *NDUFA12, NDUFB6* at diagnosis. The mean expression value for the respective gene in all mutated *CEBPA* samples was used to discretize between low and high expression. High expression of *NDUFA12, NDUFB6* were associated with shorter OS and EFS in patients with mutated *CEBPA*.


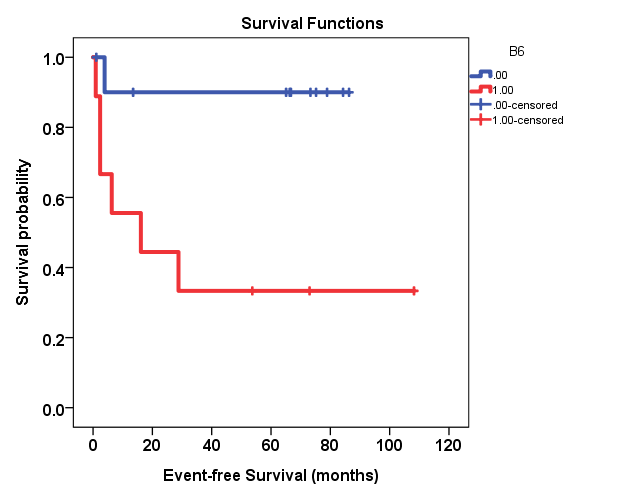

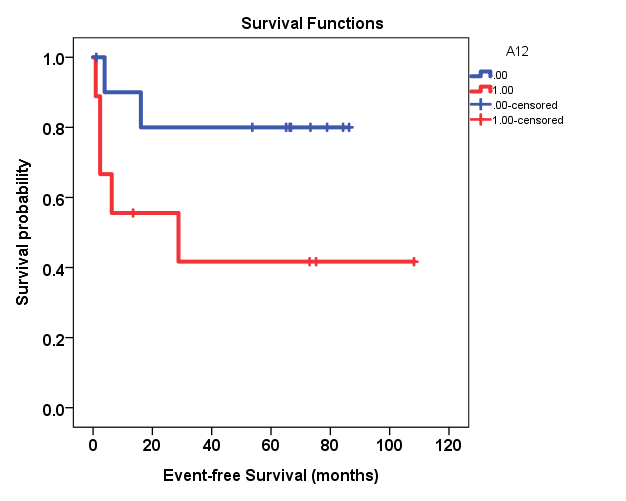


**High *NDUFA12*, n=9**

*P*=0.076

**Low *NDUFA12*, n=11**

**High *NDUFB6*, n=9**

*P*=0.013

**Low *NDUFB6,* n=11**


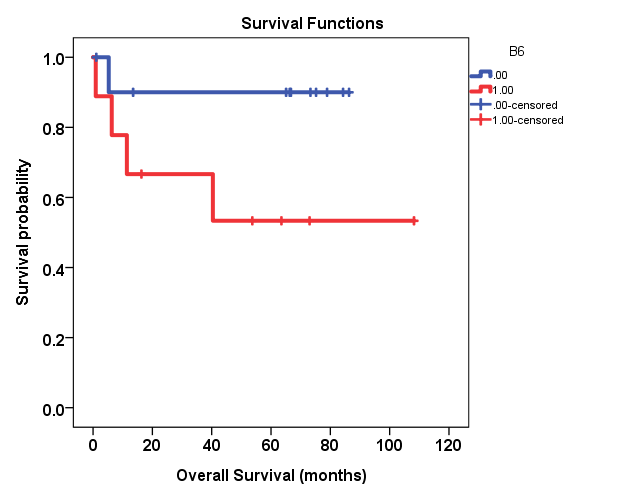

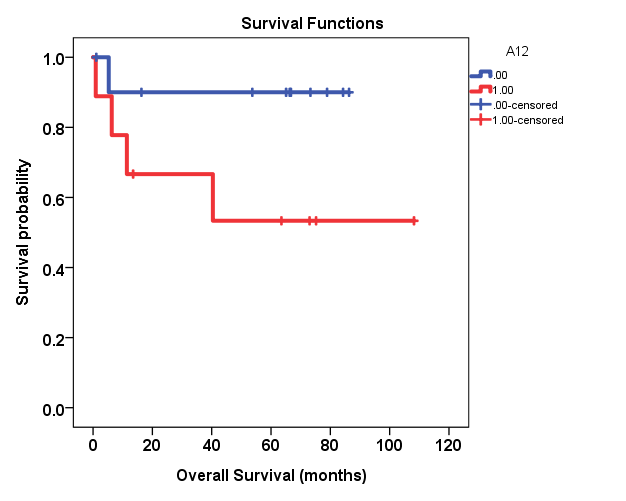


**Low *NDUFB6,* n=11**

**High *NDUFB6*, n=9**

**High *NDUFA12*, n=9**

*P*=0.102

**Low *NDUFA12*, n=11**

*P*=0.102

**Supplementary Figure 10**

**Genomic and transcriptomic determinants on outcomes in patients with *CEBPA*^nonbZIP-inf^**

(A-B) Concurrent *GATA2* mutation was associated with a trend towards longer EFS, while *TET2* mutation was associated with a trend towards shorter OS. (C) Venn diagram of DEGs of *CEBPA*^nonbZIP-inf^ (orange) with short EFS and long EFS compared with those of *CEBPA*^bZIP-inf^ (green). (D) Enriched and depleted MSigDB Hallmark and oncogenic gene sets between samples with short and long EFS.

**(A)**


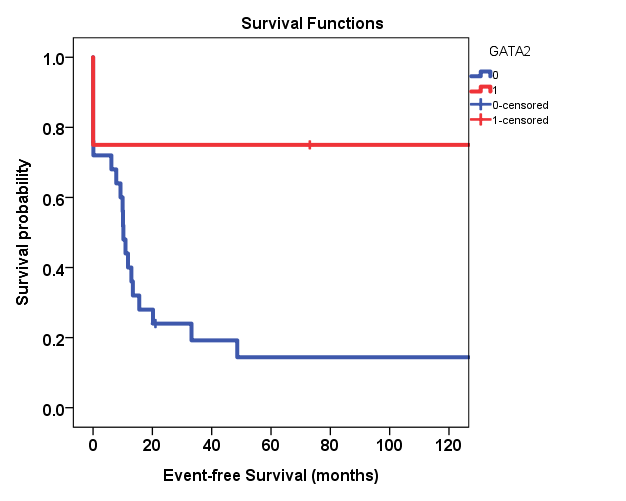

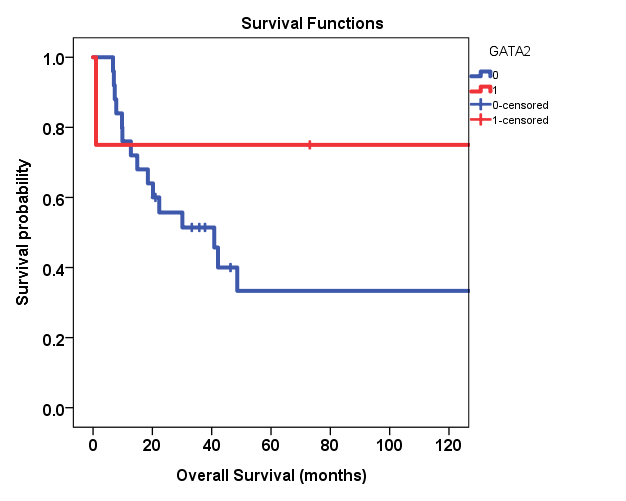


***GATA2*^mut^, n=4**

***GATA2*^wt^, n=25**

*P*=0.060

***GATA2*^mut^, n=4**

***GATA2*^wt^, n=25**

*P*=0.259

**(B)**


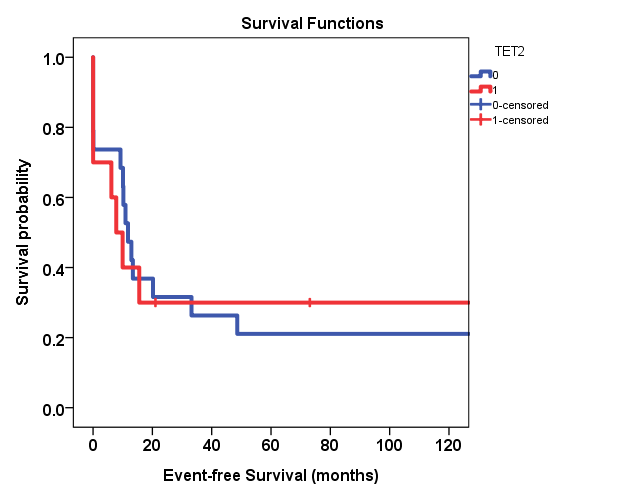

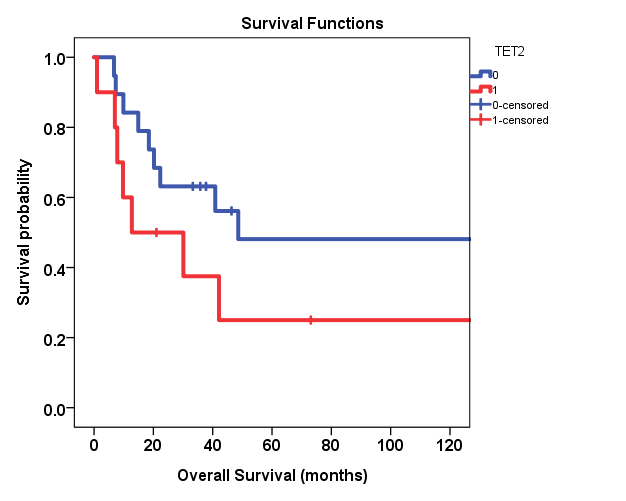


***TET2*^mut^, n=10**

***TET2*^wt^, n=19**

*P*=0.917

***TET2*^mut^, n=10**

***TET2*^wt^, n=19**

*P*=0.150

**(C)**


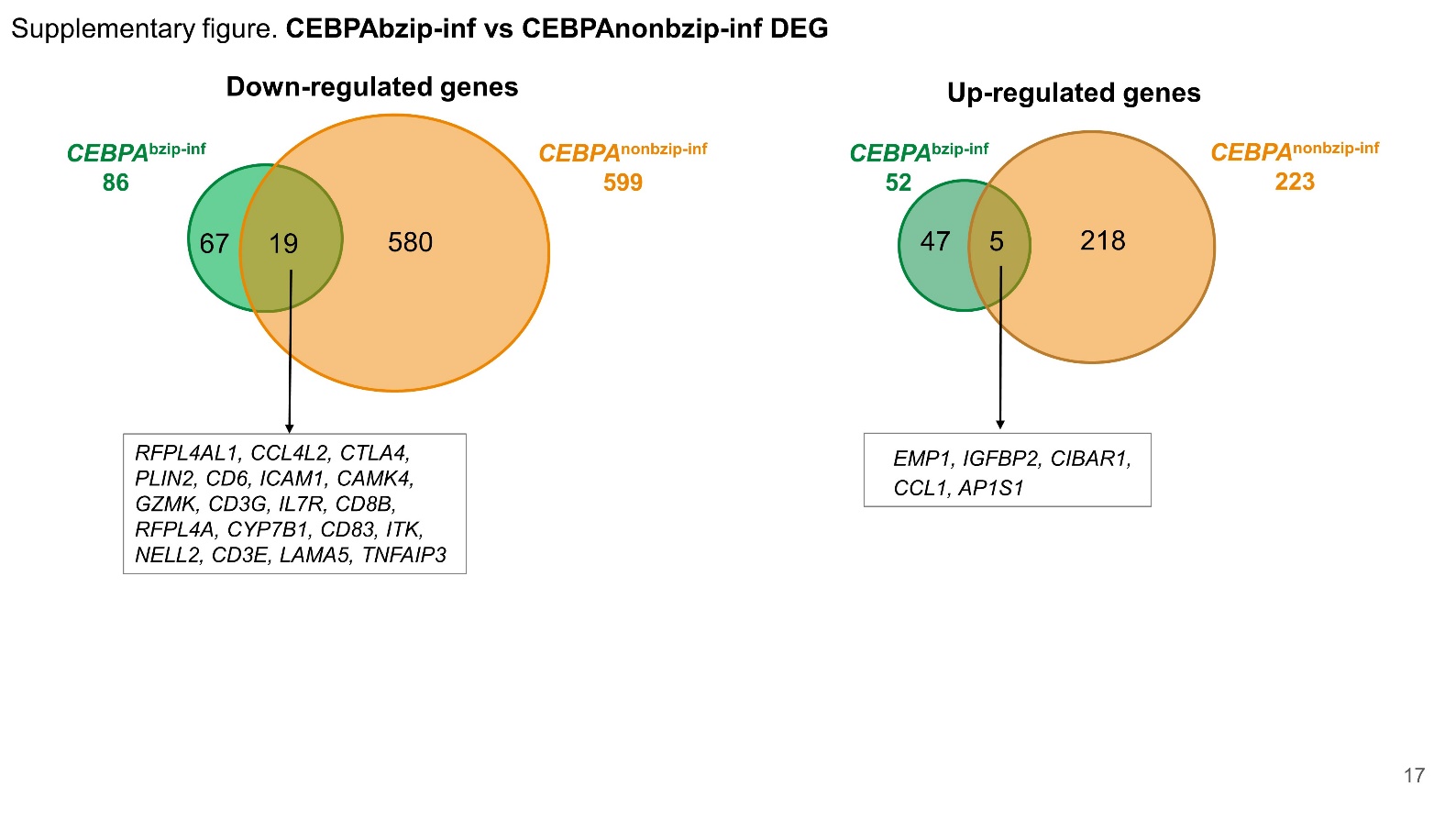


**(D)**


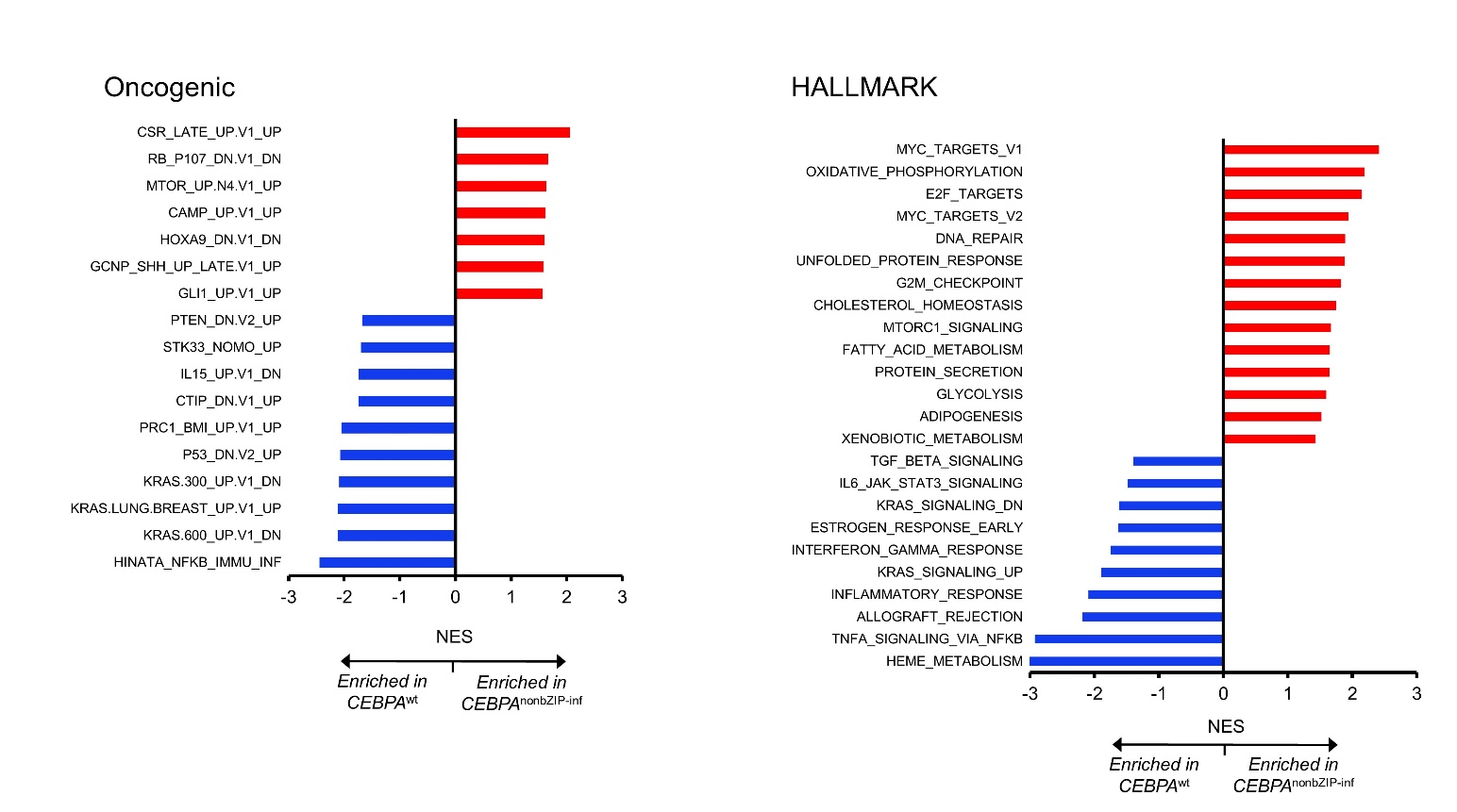


**Supplementary Figure 11**

**Enrichment of interferon signaling and metabolic pathways in *NPM1*-mutated patients with short EFS**

RNA-seq profiles were compared between *NPM1*-mutated patients with short EFS (EFS<2 years, n=34) and those with long EFS (n=26). Those with short EFS did not have a significant enrichment of metabolic pathways, such as oxidative phosphorylation (NES -1.06, FDR 0.35) and fatty acid metabolism (NES 0.67, FDR 0.97). However, there was a negative enrichment of interferon signaling (NES -3.34, FDR 0) in those with short EFS.

**
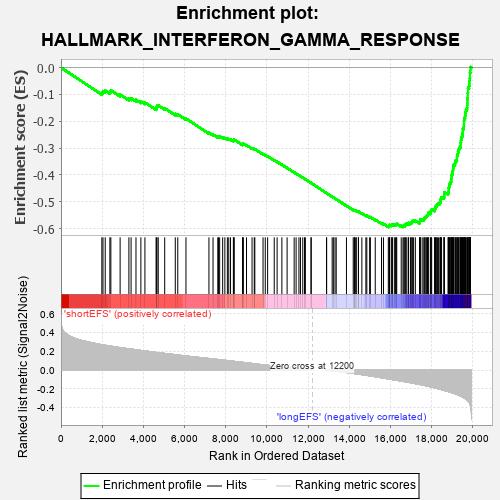

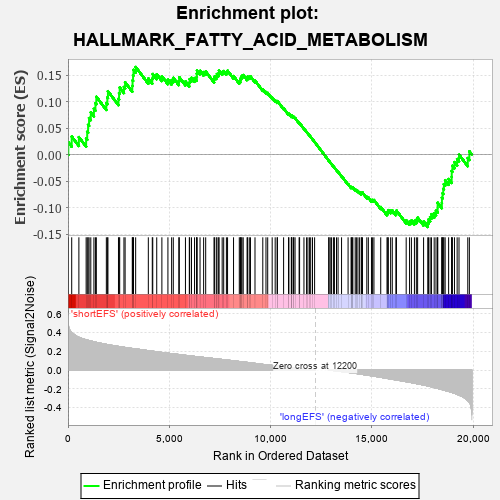

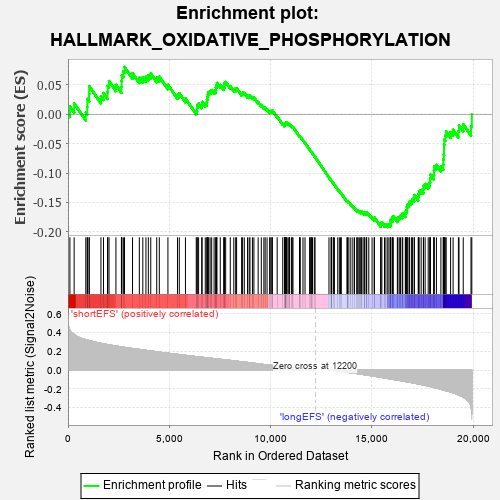
**

**NES -3.34, FDR 0**

**NES 0.67, FDR 0.97**

**NES -1.06, FDR 0.35**

**Supplementary Figure 12**

**Scatter plots illustrating the log2-transformed, CPM-normalized expression values in samples associated with short vs long EFS for leukemia-associated antigens**

**
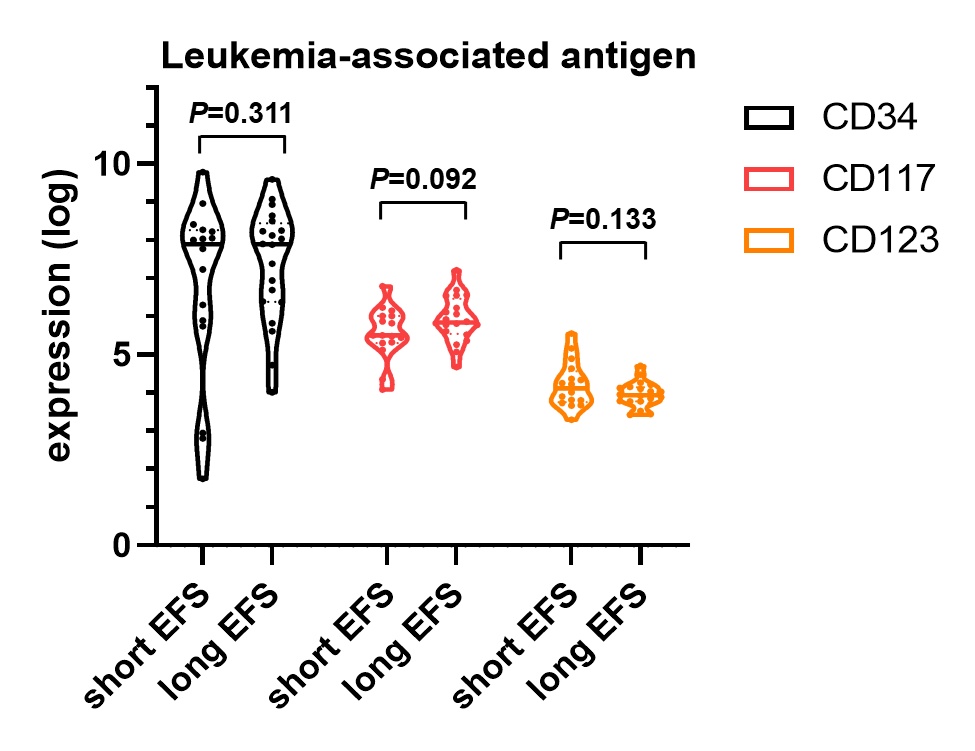
**

**Supplementary Tables**

**Supplementary Table 1. Characterization of the mutations of *CEBPA*^bZIP-inf^**

| **UPN** | **type** | **N terminal mutation** | **C terminal mutation** |
| --- | --- | --- | --- |
| 4 | *CEBPA*^sm^ |  | homozygote p.K304_T310dup |
| 5 | *CEBPA*^dm^ | p.I62Sfs*98 | p.K304_Q305insAK |
| 19 | *CEBPA*^sm^ |  | p.R297P |
| 52 | *CEBPA*^dm^ | p.H18Rfs*140 | p.L315_E316insDQRNVETQQKVL |
| 75 | *CEBPA*^dm^ | p.Q83Sfs*77 | p.V314_L315insV |
| 100 | *CEBPA*^dm^ | p.A71Gfs*37 | p.D320_N321ins11 |
| 102 | *CEBPA*^dm^ | p.Q88Hfs*75 | p.R306_N307insKQR |
| 104 | *CEBPA*^dm^ | p.Q83Sfs*77 | p.K313_L315insV |
| 106 | *CEBPA*^sm^ |  | p.R306_K313dup, p.L335Q |
| 111 | *CEBPA*^dm^ | p.Q83Sfs*77 | p.E309_T310insE |
| 119 | *CEBPA*^dm^ | p.P23Afs*84 | p.K313_L315insV |
| 122 | *CEBPA*^sm^ |  | p.Q312dup |
| 131 | *CEBPA*^dm^ | p.Q83Sfs*77 | p.K313_L315insV |
| 133 | *CEBPA*^dm^ | p.G96Cfs*15 | p.Q305dup |
| 149 | *CEBPA*^dm^ | p.D69Vfs*91 | p.K313_L315insV |
| 160 | *CEBPA*^dm^ | p.H24Pfs*85, | p.T310_Q311ins15 |
| 176 | *CEBPA*^dm^ | p.G54Vfs*47 | p.N321_D322ins12 |
| 184 | *CEBPA*^dm^ | p.A66Gfs*95 | p.V308dup |
| 192 | *CEBPA*^dm^ | p.I62Afs*95 | p.K304_Q305insAK |
| 199 | *CEBPA*^dm^ | p.F77Pfs*81 | p.Q305dup |
| 204 | *CEBPA*^dm^ | p.Y108* | p.K313_V314ins11 |
| 213 | *CEBPA*^dm^ | p.A72Hfs*90 | p.Q305P |
| 220 | *CEBPA*^dm^ | p.A111Rfs*59 | p.L315_E316ins12 |
| 221 | *CEBPA*^dm^ | p.I68Lfs*41 | p.A303_Q305del |
| 234 | *CEBPA*^dm^ | p.H24Afs*84 | p.L317dup |
| 252 | *CEBPA*^dm^ | p.Q83Sfs*77 | p.K313_L315insV |
| 261 | *CEBPA*^dm^ | p.I68Tfs*40 | p.K313dup |
| 274 | *CEBPA*^dm^ | p.I68Rfs*39 | p.K313dup |
| 277 | *CEBPA*^dm^ | p.H24Afs*84 | p.K304_E309del |
| 291 | *CEBPA*^dm^ | p.Q87Pfs*21 | p.R306_N307insKQR |
| 302 | *CEBPA*^dm^ | p.E57Sfs*103 | p.L317dup |
| 352 | *CEBPA*^dm^ | p.G36Afs*120 | p.K313dup |
| 369 | *CEBPA*^dm^ | p.A111Gfs*56 | p.D320_N321insKVLELTSD |
| 404 | *CEBPA*^dm^ | p.A66Lfs*41 | p.L315dup |
| 425 | *CEBPA*^dm^ | p.Q83Pfs*25 | p.N307_V308insD |
| 435 | *CEBPA*^dm^ | p.T60Hfs*46 | p.Q311_Q312ins16 |
| 437 | *CEBPA*^dm^ | p.L43Sfs*116 | p.R306_N307insDKAKQR |
| 438 | *CEBPA*^dm^ | p.A113Rfs*47 | p.K313dup |
| 456 | *CEBPA*^dm^ | p.Q83Sfs*77 | p.K302dup |
| 458 | *CEBPA*^dm^ | p.H24Afs*84 | p.Q312dup |
| 460 | *CEBPA*^dm^ | p.R35Gfs*125 | p.K304dup |
| 467 | *CEBPA*^dm^ | p.L78Wfs*82 | p.D320_N321ins22 |
| 478 | *CEBPA*^dm^ | p.Q88* | p.Q312dup |
| 490 | *CEBPA*^dm^ | p.I62Sfs*98 | p.V314dup |
| 498 | *CEBPA*^dm^ | p.Q83Sfs*77 | p.K313_V314delinsQ |
| 503 | *CEBPA*^dm^ | p.I62Afs*95 | p.N307_Q312del |
| 519 | *CEBPA*^dm^ | p.G96Afs*56 | p.Q312_K313del |
| 522 | *CEBPA*^sm^ |  | p.V314_L315insQKV |
| 527 | *CEBPA*^dm^ | p.A66Vfs*42 | p.N307_V308insG |
| 535 | *CEBPA*^sm^ |  | p.R316dup |
| 536 | *CEBPA*^dm^ | p.Y108Lfs*62 | p. K313_V314insQQK |
| 570 | *CEBPA*^dm^ | p.I62Afs*95 | p.E309_V314del |
| 599 | *CEBPA*^dm^ | p.D69Rfs*39 | p.L315_E316ins T |
| 607 | *CEBPA*^dm^ | p.Y108Gfs*60 | p.K313_V314insK |
| 627 | *CEBPA*^sm^ |  | p.L317_T318insQRNVETQQKVLEL |
| 652 | *CEBPA*^dm^ | p.F82Lfs*27 | p.T310dup |
| 683 | *CEBPA*^dm^ | p.S61Rfs*100 | p.K304_Q305insL |
| 703 | *CEBPA*^dm^ | p.D80Gfs*29 | p.T310_Q311insHKQRNVET |
| 709 | *CEBPA*^dm^ | p.G116Afs*44 | p.K313dup |
| 714 | *CEBPA*^dm^ | p.D107* | p.K304_Q305insKAK |
| 727 | *CEBPA*^dm^ | p.S65Rfs*10 | p.L304dup |
| 728 | *CEBPA*^dm^ | p.V95Gfs*13 | p.Q312_K313insK |
| 742 | *CEBPA*^sm^ |  | p.K313dup |
| 746 | *CEBPA*^dm^ | p.P23Rfs*137 | p.K313dup |
| 753 | *CEBPA*^dm^ | p.A44Pfs*116 | p.T310_Q311insL |
| 756 | *CEBPA*^dm^ | p.P45Hfs*115 | p.Q305dup |
| 812 | *CEBPA*^dm^ | p.P45Hfs*115 | p.L315_E316insV |
| 817 | *CEBPA*^dm^ | p.H24Rfs*120 | p.E316_L317ins11 |
| 820 | *CEBPA*^sm^ |  | p.Q311_Q312insKQRNVETQ |
| 840 | *CEBPA*^dm^ | p.P23Rfs*137 | p.K313dup |
| 854 | *CEBPA*^dm^ | p.A37Sfs*119 | p.I294_V296delinsRES |
| 858 | *CEBPA*^dm^ | p.E59** | p.D301_K302insGlySer |
| 869 | *CEBPA*^dm^ | p.A44Pfs*116 | p.K313dup |
| 872 | *CEBPA*^sm^ |  | p.K304_Q305insL |
| 879 | *CEBPA*^dm^ | p.A37Lfs*118, p.A44Pfs*116 | p.K313dup |
| 907 | *CEBPA*^dm^ | p.D107Ffs*53 | p.Q312dup |
| 932 | *CEBPA*^dm^ | p.G96Afs*56 | p.K313dup |
| 938 | *CEBPA*^sm^ |  | p.E316G |
| 941 | *CEBPA*^dm^ | p.P23Rfs*137 | p.R297P |
| 957 | *CEBPA*^sm^ |  | p.T310dup |
| 974 | *CEBPA*^dm^ | p.A66Vfs**94 | p.Q312del |
| 976 | *CEBPA*^sm^ |  | p.Q311P |
| 988 | *CEBPA*^dm^ | p.G100fs**7 | p.T310_Q311del |
| 991 | *CEBPA*^dm^ | p.A37Lfs*118 | p.E316_L317insQ |
| 999 | *CEBPA*^dm^ | p.F82Lfs*26 | p.E309dup |
| 1001 | *CEBPA*^sm^ |  | p.L335Q |
| 1025 | *CEBPA*^dm^ | p.I62indels | p.I318indels |
| 1038 | *CEBPA*^dm^ | p.Y108Lfs*61 | p.K304_Q305insL |
| 1045 | *CEBPA*^dm^ | p.Y108* | p.K304_R306dup |
| 1068 | *CEBPA*^dm^ | p.H24Afs*24 | p.V314_L315insHVETQQKV |
| 1086 | *CEBPA*^sm^ |  | p.K313dup |
| 1099 | *CEBPA*^dm^ | p.A93Gfs*15 | p.A303_K304del |
| 1113 | *CEBPA*^dm^ | p.A25_A29dup | p.K304_R306dup |
| 1119 | *CEBPA*^dm^ | p.G96Afs*56 | p.K312dup |
| 1121 | *CEBPA*^dm^ | p.I62Sfs*98 | c.930indels |
| 1135 | *CEBPA*^dm^ | c.183indels | p.Q311_Q312insL |
| 1155 | *CEBPA*^dm^ | p.S27Kfs*80 | c.895_964dup |
| 1160 | *CEBPA*^sm^ |  | P.L317_T318insR, homozygous |
| 1163 | *CEBPA*^dm^ | p.G96Afs*189 | p.K312dup |
| 1288 | *CEBPA*^dm^ | p.R23Pfs*136 | p.T310_Q311dup |
| 1368 | *CEBPA*^dm^ | p.E59* | p.G309_T310insQ |
| 1393 | *CEBPA*^dm^ | p.Q87* | p.T310dup |
| 1428 | *CEBPA*^dm^ | p.G53Afs*103 | P.Q312del |
| 1478 | *CEBPA*^dm^ | p.H24Afs*84 | p.K313_V314insM |
| 1483 | *CEBPA*^dm^ | p.I68Pfs*93 | p.K313dup |
| 1539 | *CEBPA*^dm^ | p.E59* | p.E316-L317insR |
| 1547 | *CEBPA*^dm^ | p.E57* | p.R306_N307insKVETQR |
| 1578 | *CEBPA*^dm^ | p.V95Rfs*68 | p.Q312dup |
| 1621 | *CEBPA*^sm^ |  | p.Q312dup |
| 1632 | *CEBPA*^dm^ | p.L78Pfs*31 | p.K313dup |
| 1652 | *CEBPA*^dm^ | p.Y67* | p.V308dup |
| 1677 | *CEBPA*^dm^ | p.S21Rfs*139 | p.K313dup |
| 1693 | *CEBPA*^dm^ | p.H24Afs*84 | p.K313dup |

Abbreviations: *CEBPA*^dm^, *CEBPA* double mutation; *CEBPA*^sm^, *CEBPA* single mutation; UPN, unique patient number

**Supplementary Table 2. Comparison of other genetic alterations between patients with AML according to their *CEBPA* statuses**

| **Mutation** | **Total pts examined** | **Pts with the other gene mutations (%)** | | | | ***P* value^a^** | ***P* value**^b^ | ***P* value**^c^ |
| --- | --- | --- | --- | --- | --- | --- | --- | --- |
|  |  | **Whole cohort** | ***CEBPA*^wt^** | ***CEBPA*^non-bZIP-inf^** | ***CEBPA*^bZIP-inf^** |  |  |  |
| **Activated signaling** |  |  |  |  |  |  |  |  |
| ***FLT3-*ITD** | 882 | 15.4 | 19.0 | 13.8 | 11.8 | 0.587 | 0.058 | 0.755 |
| ***FLT3-*TKD** | 880 | 8.1 | 8.5 | 10.3 | 4.5 | 0.499 | 0.141 | 0.363 |
| ***NRAS*** | 883 | 14.7 | 15.3 | 6.9 | 12.7 | 0.409 | 0.435 | 0.523 |
| ***KRAS*** | 882 | 4.9 | 5.4 | 0 | 2.7 | 0.391 | 0.222 | >0.999 |
| ***PTPN11*** | 880 | 2.7 | 5.9 | 0 | 0 | 0.395 | 0.008 | - |
| ***KIT*** | 800 | 5.6 | 6.1 | 0 | 3.6 | 0.397 | 0.289 | 0.580 |
| ***JAK2*** | 819 | 0.7 | 0.9 | 0 | 0 | >0.999 | >0.999 | - |
| ***CBL-b*** | 866 | 0 | 0 | 0 | 0 | - | - | - |
| ***c-CBL*** | 866 | 0.8 | 0.8 | 3.4 | 0 | 0.225 | >0.999 | 0.216 |
| **Transcription factors** |  |  |  |  |  |  |  |  |
| ***RUNX1*** | 879 | 11.0 | 12.4 | 10.3 | 1.8 | 0.763 | 0.002 | 0.061 |
| ***GATA2*** | 873 | 7.8 | 4.4 | 13.8 | 29.1 | 0.034 | <0.0001 | 0.094 |
| ***ETV6*** | 863 | 1.6 | 1.9 | 0 | 0 | >0.999 | 0.237 | - |
| **DNA methylation** |  |  |  |  |  |  |  |  |
| ***TET2*** | 878 | 10.6 | 9.6 | 34.5 | 10.9 | <0.0001 | 0.713 | 0.004 |
| ***DNMT3A*** | 880 | 16.8 | 18.6 | 13.8 | 5.5 | 0.802 | <0.0001 | 0.217 |
| ***IDH1*** | 880 | 5.1 | 5.7 | 10.3 | 0 | 0.664 | 0.031 | 0.008 |
| ***IDH2*** | 880 | 7.5 | 13.5 | 13.8 | 0.9 | 0.776 | <0.0001 | 0.007 |
| ***WT1*** | 880 | 8.1 | 7.2 | 6.9 | 14.5 | >0.999 | 0.004 | 0.364 |
| **Chromatin modifiers** |  |  |  |  |  |  |  |  |
| ***ASXL1*** | 880 | 9.9 | 10.7 | 10.3 | 4.5 | >0.999 | 0.081 | 0.363 |
| ***EZH2*** | 864 | 1.0 | 0.8 | 0 | 2.9 | >0.999 | 0.096 | >0.999 |
| ***KMT2A-*PTD** | 882 | 3.1 | 3.5 | 3.4 | 0 | >0.999 | 0.044 | 0.209 |
| ***SETBP1*** | 570 | 1.9 | 2.3 | 0 | 0 | >0.999 | 0.374 | - |
| ***BCOR*** | 864 | 2.0 | 2.1 | 3.4 | 1.0 | 0.444 | 0.708 | 0.387 |
| ***PHF6*** | 863 | 2.1 | 2.3 | 3.4 | 0 | 0.484 | 0.150 | 0.216 |
| **Splicing** |  |  |  |  |  |  |  |  |
| ***SF3B1*** | 874 | 2.6 | 3.0 | 0 | 0.9 | >0.999 | 0.345 | >0.999 |
| ***U2AF1*** | 873 | 3.7 | 3.9 | 7.1 | 0.9 | 0.286 | 0.164 | 0.105 |
| ***SRSF2*** | 874 | 4 | 4.5 | 7.1 | 0 | 0.338 | 0.016 | 0.040 |
| ***ZRSR2*** | 558 | 1.3 | 1.3 | 0 | 1.5 | >0.999 | >0.999 | >0.999 |
| **Cohesin genes** | 863 | 4.6 | 4.5 | 6.9 | 4.6 | 0.361 | >0.999 | 0.639 |
| ***STAG1*** | 869 | 0.1 | 0.1 | 0 | 0 | >0.999 | >0.999 | - |
| ***STAG2*** | 868 | 3.6 | 3.7 | 10.3 | 0.9 | 0.086 | 0.160 | 0.030 |
| ***RAD21*** | 869 | 2.3 | 1.9 | 3.4 | 4.6 | 0.422 | 0.090 | >0.999 |
| ***SMC1*** | 870 | 2.1 | 2.2 | 0 | 1.9 | >0.999 | >0.9999 | >0.999 |
| ***SMC3*** | 869 | 0.8 | 0.7 | 3.4 | 0.9 | 0.196 | 0.569 | 0.380 |
| ***NPM1*** | 883 | 19.7 | 22.0 | 24.1 | 2.7 | 0.633 | <0.0001 | 0.001 |
| ***TP53*** | 880 | 5.3 | 6.1 | 6.9 | 0 | 0.678 | 0.007 | 0.042 |

Abbreviations: *CEBPA*^dm^, *CEBPA* double mutation; *FLT3^wt^*, *FLT3*-ITD wild-type; pts, patients

^a^*CEBPA*^nonbZIP-inf^ patients *vs.* *CEBPA*^wt^ patients

^b^*CEBPA*^bZIP-inf^ patients *vs.* *CEBPA*^wt^ patients

^c^*CEBPA*^bZIP-inf^ patients *vs.* *CEBPA*^nonbZIP-inf^ patients

**Supplementary Table 3. Characteristics of 26 *CEBPA*^bZIP-inf^ patients who received allogeneic hematopoietic stem cell transplantation in CR1**

| **UPN** | **Age** | **Cytogenetics** | **Co-Mutations** | **HSCT Indication** | **Transplant type** | **Relapsed after HSCT** |
| --- | --- | --- | --- | --- | --- | --- |
| 4 | 25 | +21 | Nil | Delayed CR1,  Hyperleukocytosis | Matched sibling | No |
| 52 | 32 | NK | *FLT3*-ITD | Hyperleukocytosis,  *FLT3*-ITD | Matched sibling | No |
| 204 | 32 | +10 | *GATA2, NRAS* | Delayed CR1 | Matched sibling | No |
| 234 | 25 | NK | Nil | Hyperleukocytosis | Matched sibling | No |
| 261 | 28 | NK | *GATA2* | Delayed CR1 | Matched sibling | No |
| 277 | 22 | del(9q) | *GATA2* | Hyperleukocytosis | Matched sibling | No |
| 478 | 45 | +4, del(9)(q13q22), +10 | *WT1* | Delayed CR1 | Matched sibling | No |
| 536 | 31 | NK | *GATA2, BCOR* | Delayed CR1 | Matched sibling | No |
| 599 | 32 | del(9)(q22q34) | Nil | MRD positive | Matched sibling | No |
| 627 | 20 | NK | *GATA2, ASXL1* | Hyperleukocytosis | MUD | Yes |
| 728 | 49 | NK | *FLT3*-ITD*, DNMT3A* | Hyperleukocytosis | MMUD | No |
| 812 | 19 | NK | *FLT3*-ITD | Hyperleukocytosis | MMUD | No |
| 854 | 40 | t(6;9)(p23;q34) | *STAG2* | Delayed CR1 | Mismatched sibling | No |
| 869 | 30 | NK | *WT1* | Hyperleukocytosis | Matched sibling | No |
| 938 | 45 | NK | *NPM1, FLT3*-TKD*, DNMT3A* | Hyperleukocytosis | Matched sibling | No |
| 974 | 52 | NK | *GATA2, TET2, WT1, NRAS* | Hyperleukocytosis | Matched sibling | Yes |
| 999 | 44 | NK | *GATA2* | MRD positive | MUD | No |
| 1025 | 38 | NK | *FLT3*-ITD*, WT1* | *FLT3*-ITD | Matched sibling | No |
| 1113 | 35 | NK | *NRAS* | Hyperleukocytosis | Haploidentical | No |
| 1121 | 23 | NK | *GATA2, TET2* | Hyperleukocytosis | Matched sibling | Yes |
| 1155 | 34 | del(7)(q11q22) | *GATA2, EZH2, RAD21* | Adverse cytogenetics | Matched sibling | No |
| 1288 | 31 | NA | NA | Delayed CR1 | MUD | No |
| 1478 | 33 | del(9)(q13q22),+21 | *WT1, CSF3R* | Delayed CR1 | Haploidentical | No |
| 1483 | 50 | del(7)(p11p15) | *FLT3*-ITD | *FLT3*-ITD | MMUD | No |
| 1539 | 45 | del(9)(q22q34) | Nil | MRD positive | MUD | Yes |
| 1621 | 55 | NK | *FLT3*-ITD | Delayed CR1, MRD positive | Haploidentical | No |

Abbreviations: CR, complete remission; HSCT, hematopoietic stem cell transplantation; MRD, measurable residual disease, MUD, matched unrelated donor; MMUD, mismatched unrelated donor; NK, normal karyotype; OS, overall survival; UPN, unique patient number

**Supplementary Table 4. Characteristics of 150 *CEBPA*^dm^ patients in the validation cohort**

| **Cohort** | **Patient** | **Age** | ***DNMT3A m*utation** | **OS (months)** | **OS status** |
| --- | --- | --- | --- | --- | --- |
| AMLSG | 10796 | 53 | wt | 34.22 | alive |
| AMLSG | 10809 | 44 | wt | 63.29 | alive |
| AMLSG | 10811 | 51 | wt | 65.72 | alive |
| AMLSG | 10815 | 50 | wt | 55.17 | alive |
| AMLSG | 10818 | 34 | wt | 60.16 | alive |
| AMLSG | 10830 | 51 | wt | 32.25 | death |
| AMLSG | 10861 | 49 | wt | 66.25 | alive |
| AMLSG | 10866 | 23 | wt | 60.92 | alive |
| AMLSG | 10876 | 56 | wt | 26.10 | death |
| AMLSG | 10890 | 44 | wt | 53.65 | alive |
| AMLSG | 10919 | 41 | mutated | 6.41 | alive |
| AMLSG | 10970 | 52 | wt | 44.42 | alive |
| AMLSG | 11001 | 51 | wt | 29.46 | alive |
| AMLSG | 11050 | 37 | wt | 41.23 | alive |
| AMLSG | 11053 | 42 | wt | 38.37 | alive |
| AMLSG | 11063 | 37 | wt | 12.92 | death |
| AMLSG | 11090 | 53 | wt | 43.99 | alive |
| AMLSG | 11106 | 36 | wt | 5.75 | death |
| AMLSG | 11233 | 77 | wt | 0.49 | death |
| AMLSG | 7607 | 38 | wt | 41.85 | alive |
| AMLSG | 7614 | 34 | wt | 63.85 | alive |
| AMLSG | 7619 | 35 | wt | 136.14 | alive |
| AMLSG | 7622 | 38 | wt | 86.04 | alive |
| AMLSG | 7723 | 32 | wt | 73.22 | alive |
| AMLSG | 7728 | 50 | wt | 21.83 | death |
| AMLSG | 7748 | 38 | wt | 77.56 | alive |
| AMLSG | 7749 | 38 | wt | 76.31 | alive |
| AMLSG | 7765 | 59 | wt | 60.13 | death |
| AMLSG | 7766 | 37 | wt | 3.22 | death |
| AMLSG | 7789 | 51 | mutated | 3.32 | death |
| AMLSG | 7794 | 48 | wt | 132.56 | alive |
| AMLSG | 7894 | 23 | wt | 111.09 | alive |
| AMLSG | 7896 | 53 | wt | 17.82 | death |
| AMLSG | 7898 | 28 | wt | 45.07 | death |
| AMLSG | 7916 | 41 | wt | 59.57 | death |
| AMLSG | 7973 | 50 | wt | 131.18 | alive |
| AMLSG | 7978 | 59 | wt | 107.08 | alive |
| AMLSG | 7996 | 37 | wt | 47.80 | alive |
| AMLSG | 8000 | 42 | wt | 43.86 | death |
| AMLSG | 8014 | 39 | wt | 62.73 | alive |
| AMLSG | 8018 | 34 | wt | 79.79 | alive |
| AMLSG | 8043 | 55 | wt | 122.30 | alive |
| AMLSG | 8074 | 52 | wt | 50.30 | alive |
| AMLSG | 8079 | 60 | wt | 6.84 | death |
| AMLSG | 8111 | 51 | wt | 59.18 | death |
| AMLSG | 8124 | 37 | wt | 0.59 | death |
| AMLSG | 8126 | 35 | wt | 3.09 | death |
| AMLSG | 8178 | 38 | wt | 88.57 | alive |
| AMLSG | 8180 | 44 | mutated | 92.09 | alive |
| AMLSG | 8200 | 42 | wt | 16.44 | death |
| AMLSG | 8219 | 49 | wt | 90.71 | alive |
| AMLSG | 8229 | 38 | wt | 84.23 | alive |
| AMLSG | 8249 | 51 | wt | 77.03 | alive |
| AMLSG | 8268 | 54 | mutated | 79.17 | alive |
| AMLSG | 8277 | 51 | wt | 27.72 | death |
| AMLSG | 8284 | 20 | wt | 82.09 | alive |
| AMLSG | 8287 | 59 | wt | 88.60 | alive |
| AMLSG | 8292 | 45 | wt | 92.32 | alive |
| AMLSG | 8318 | 47 | wt | 76.67 | alive |
| AMLSG | 8340 | 51 | wt | 77.03 | alive |
| AMLSG | 8347 | 41 | wt | 72.62 | alive |
| AMLSG | 8351 | 43 | wt | 18.25 | alive |
| AMLSG | 8389 | 50 | wt | 76.08 | alive |
| AMLSG | 8405 | 56 | wt | 16.21 | alive |
| AMLSG | 8437 | 59 | wt | 56.68 | alive |
| AMLSG | 8475 | 37 | wt | 22.13 | death |
| AMLSG | 8506 | 56 | mutated | 17.00 | death |
| AMLSG | 8585 | 44 | wt | 5.65 | death |
| AMLSG | 9222 | 41 | wt | 91.33 | alive |
| AMLSG | 9317 | 54 | wt | 97.25 | alive |
| AMLSG | 9330 | 31 | wt | 59.51 | alive |
| AMLSG | 9342 | 25 | wt | 32.32 | alive |
| AMLSG | 9356 | 38 | wt | 59.84 | alive |
| AMLSG | 10796 | 53 | wt | 34.22 | alive |
| AMLSG | 10809 | 44 | wt | 63.29 | alive |
| AMLSG | 10811 | 51 | wt | 65.72 | alive |
| AMLSG | 10815 | 50 | wt | 55.17 | alive |
| AMLSG | 10818 | 34 | wt | 60.16 | alive |
| AMLSG | 10830 | 51 | wt | 32.25 | death |
| AMLSG | 10861 | 49 | wt | 66.25 | alive |
| AMLSG | 10866 | 23 | wt | 60.92 | alive |
| AMLSG | 10876 | 56 | wt | 26.10 | death |
| AMLSG | 10890 | 44 | wt | 53.65 | alive |
| AMLSG | 10919 | 41 | mutated | 6.41 | alive |
| AMLSG | 10970 | 52 | wt | 44.42 | alive |
| AMLSG | 11001 | 51 | wt | 29.46 | alive |
| UK_NCRI | PD14877c | 84 | mutated | 0.79 | death |
| UK_NCRI | PD14902a | 80 | mutated | 5.39 | death |
| UK_NCRI | PD14972c | 59 | mutated | 91.07 | alive |
| UK_NCRI | PD15045c | 34 | mutated | 63.18 | alive |
| UK_NCRI | PD15070a | 63 | wt | 2.53 | death |
| UK_NCRI | PD15213a | 79 | wt | 17.64 | death |
| UK_NCRI | PD15357c | 75 | wt | 16.92 | death |
| UK_NCRI | PD15384a | 66 | wt | 2.23 | death |
| UK_NCRI | PD15449c | 56 | wt | 58.91 | death |
| UK_NCRI | PD15459c | 61 | wt | 10.38 | death |
| UK_NCRI | PD19694a | 51 | wt | 62.72 | alive |
| UK_NCRI | PD19748c | 40 | wt | 73.26 | alive |
| UK_NCRI | PD19762c | 32 | wt | 77.34 | alive |
| UK_NCRI | PD19769c | 38 | wt | 78.13 | alive |
| UK_NCRI | PD19855a | 37 | wt | 100.27 | alive |
| UK_NCRI | PD19856a | 52 | wt | 105.07 | alive |
| UK_NCRI | PD19913c | 59 | wt | 111.97 | alive |
| UK_NCRI | PD19917a | 40 | wt | 30.82 | death |
| UK_NCRI | PD19920a | 46 | wt | 111.08 | alive |
| UK_NCRI | PD19962c | 42 | wt | 66.20 | alive |
| UK_NCRI | PD19983a | 19 | wt | 123.60 | alive |
| UK_NCRI | PD20034a | 66 | mutated | 6.80 | death |
| UK_NCRI | PD20111a | 63 | mutated | 0.10 | death |
| UK_NCRI | PD20142c | 76 | wt | 8.77 | death |
| UK_NCRI | PD20192c | 60 | wt | 85.32 | alive |
| UK_NCRI | PD20329a | 80 | wt | 6.74 | death |
| UK_NCRI | PD20333c | 51 | wt | 21.52 | death |
| UK_NCRI | PD22624a | 38 | wt | 90.58 | alive |
| UK_NCRI | PD22652a | 48 | wt | 2.04 | death |
| UK_NCRI | PD22694a | 43 | wt | 4.04 | death |
| UK_NCRI | PD22727c | 40 | wt | 80.79 | death |
| UK_NCRI | PD22844c | 17 | wt | 101.78 | alive |
| UK_NCRI | PD22943a | 40 | wt | 96.56 | alive |
| UK_NCRI | PD22956c | 58 | wt | 5.22 | death |
| UK_NCRI | PD23092a | 35 | wt | 18.43 | death |
| UK_NCRI | PD23176c | 26 | wt | 50.86 | death |
| UK_NCRI | PD23254a | 23 | wt | 73.40 | alive |
| UK_NCRI | PD23301a | 60 | mutated | 63.90 | alive |
| UK_NCRI | PD25026a | 58 | wt | 72.57 | death |
| UK_NCRI | PD25091a | 24 | wt | 74.02 | alive |
| UK_NCRI | PD25105a | 44 | wt | 70.87 | alive |
| UK_NCRI | PD25136c | 32 | wt | 71.43 | alive |
| UK_NCRI | PD25137c | 30 | wt | 4.70 | death |
| UK_NCRI | PD25225c | 43 | wt | 6.01 | alive |
| UK_NCRI | PD25316c | 34 | wt | 38.34 | death |
| UK_NCRI | PD25362a | 67 | wt | 47.97 | alive |
| UK_NCRI | PD25390a | 34 | wt | 58.87 | alive |
| UK_NCRI | PD25461c | 56 | wt | 9.23 | death |
| UK_NCRI | PD25507a | 71 | wt | 35.75 | death |
| UK_NCRI | PD25618c | 84 | wt | 1.31 | death |
| UK_NCRI | PD25620c | 80 | wt | 3.81 | death |
| UK_NCRI | PD14877c | 84 | mutated | 0.79 | death |
| UK_NCRI | PD14902a | 80 | mutated | 5.39 | death |
| UK_NCRI | PD14972c | 59 | mutated | 91.07 | alive |
| UK_NCRI | PD15045c | 34 | mutated | 63.18 | alive |
| UK_NCRI | PD15070a | 63 | wt | 2.53 | death |
| UK_NCRI | PD15213a | 79 | wt | 17.64 | death |
| TCGA | AB-2845-03 | 37 | wt | 10.1 | death |
| TCGA | AB-2955-03 | 56 | mutated | 16.3 | death |
| TCGA | AB-2979 | 30 | wt | 22.4 | alive |
| TCGA | AB-3000 | 25 | wt | 41.6 | alive |
| NTUH-YL | 509369 | 56 | wt | 6.3 | alive |
| NTUH-YL | 955349 | 34 | wt | 15.8 | alive |
| NTUH-YL | 378579 | 54 | wt | 16.2 | alive |
| NTUH-YL | 711763 | 73 | wt | 14.5 | death |
| NTUH-YL | 998093 | 28 | wt | 26.9 | alive |
| NTUH-YL | 431845 | 45 | wt | 29.4 | alive |
| NTUH-YL | 050382 | 41 | wt | 22.3 | alive |
| NTUH-YL | 794168 | 27 | wt | 36.23 | alive |
| NTUH-YL | 812245 | 34.9 | wt | 26.63 | alive |
| NTUH-YL | 044963 | 38.7 | wt | 25.87 | alive |
| NTUH-YL | 909024 | 35.5 | wt | 21.83 | alive |
| NTUH-YL | 938327 | 56.7 | wt | 20.97 | alive |
| NTUH-YL | 950981 | 39.1 | wt | 19.60 | alive |
| NTUH-YL | 200814 | 21.8 | wt | 16.70 | alive |
| NTUH-YL | 545960 | 52.3 | wt | 16.70 | alive |
| NTUH-YL | 150432 | 44.0 | wt | 11.23 | alive |
| NTUH-YL | 103098 | 32.6 | wt | 8.37 | alive |
| NTUH-YL | 124522 | 51.2 | wt | 5.50 | alive |
| NTUH-YL | 052242 | 79 | wt | 7.47 | alive |
| NTUH-YL | 623163 | 89.6 | wt | 3.03 | alive |
| NTUH-YL | 770269 | 35 | wt | 35.8 | alive |
| NTUH-YL | 388401 | 75 | wt | 10.8 | death |
| NTUH-YL | 509369 | 56 | wt | 6.3 | alive |
| NTUH-YL | 955349 | 34 | wt | 15.8 | alive |
| NTUH-YL | 378579 | 54 | wt | 16.2 | alive |
| NTUH-YL | 711763 | 73 | mutated | 14.5 | death |

Abbreviations: OS, overall survival; wt, wild-type

**Supplementary Table 5. Characteristics of 39 *CEBPA*^bZIP-inf^ patients who had experienced relapse**

| **UPN** | **Age** | **Cytogenetics** | **Co-Mutations** | **Response to 2^nd^ line** | **CR1 duration** | **Transplant status** | **Relapsed after HSCT** | **OS from relapse (months)** |
| --- | --- | --- | --- | --- | --- | --- | --- | --- |
| 5 | 60 | NK | *GATA2* | CR2 | 6.0 | - | - | 26.5 (death) |
| 106 | 49 | NK | Nil | CR2 | 16.9 | CR2 | No | 8.3 (death) |
| 111 | 56 | NK | *GATA2, TET2* | CR2 | 15.1 | - | - | 219.6 (alive) |
| 131 | 50 | NK | *GATA2* | Refractory | 16.0 | Refractory | No | 9.2 (death) |
| 133 | 34 | del(9)(q13q32) | *FLT3*-ITD | CR2 | 3.6 | - | - | 14.0 (death) |
| 149 | 55 | NK | *GATA2* | - | 60.4 | - | - | 1.5 (death) |
| 160 | 64 | NK | Nil | CR2 | 10.5 | - | - | 12.9 (death) |
| 192 | 37 | NK | *GATA2* | - | 92.8 | 1^st^ relapse | No | 16.9 (death) |
| 220 | 18 | NK | *FLT3*-ITD*, FLT3*-TKD | CR2 | 17.0 | CR3 | No | 19.9 (death) |
| 221 | 33 | NK | Nil | CR2 | 7.0 | - | - | 15.0 (death) |
| 274 | 58 | NK | *TET2, RAD21* | CR2 | 10.1 | - | - | 21.1 (death) |
| 291 | 67 | NK | Nil | CR2 | 7.8 | - | - | 7.6 (death) |
| 302 | 50 | i(21q) | Nil | - | 5.6 | - | - | 6.3 (death) |
| 352 | 50 | +21 | Nil | CR2 | 138.2 | - | - | 23.4 (death) |
| 369 | 52 | -X,+21 | *WT1* | Refractory | 11.5 | - | - | 2.5 (death) |
| 425 | 57 | del (11)(q14q23) | *RAD21* | - | 6.1 | 1st relapse | No | 124.0 (death) |
| 467 | 36 | -Y | *GATA2* | CR2 | 2.9 | CR2 | No | 126.2 (alive) |
| 503 | 36 | NK | *GATA2, NRAS* | CR2 | 34.2 | CR2 | No | 97.5 (alive) |
| 522 | 21 | NK | *NRAS, KIT* | CR2 | 6.2 | CR2 | No | 156.1 (alive) |
| 570 | 32 | NK | Nil | CR2 | 15.6 | CR2 | No | 90.7 (alive) |
| 627 | 20 | NK | *GATA2, ASXL1* | CR2 | 16.5 | CR1 | Yes | 52.9 (death) |
| 683 | 60 | NK | *WT1* | Refractory | 9.4 | - | - | 1.4 (death) |
| 703 | 36 | NK | *FLT3-TKD, WT1* | Refractory | 9.3 | Refractory | No | 129.5 (alive) |
| 727 | 49 | der(9)del(9)(p13p22)del(9)(q22q33) | *NRAS, U2AF1* | CR2 | 18.0 | CR2 | No | 117.2 (alive) |
| 753 | 39 | NK | Nil | CR2 | 15.7 | CR2 | No | 89.6 (alive) |
| 756 | 56 | NK | *TET2, WT1* | CR2 | 10.6 | CR2 | Yes | 68.2 (death) |
| 820 | 35 | NK | *GATA2, DNMT3A, IDH2, NRAS, KIT* | Refractory | 8.9 | Refractory | Yes | 15.1 (death) |
| 941 | 31 | NK | *TET2* | CR2 | 50.8 | CR2 | No | 4.3 (alive) |
| 974 | 52 | NK | *GAT2, TET2, WT1, NRAS* | CR2 | 15.1 | CR1 | Yes | 13.1 (death) |
| 988 | 46 | NK | Nil | CR2 | 6.7 | CR3 | Yes | 33.0 (death) |
| 991 | 18 | NK | *GATA2* | CR2 | 23.0 | CR2 | No | 77.4 (alive) |
| 1001 | 66 | NK | *NPM1, WT1, DNMT3A* | CR2 | 3.30 | - | - | 2.8 (death) |
| 1038 | 19 | NK | *GATA2, TET2, NRAS* | CR2 | 11.2 | CR2 | Yes | 70.9 (death) |
| 1086 | 32 | inv(9)(p13q22) | Nil | CR2 | 9.3 | CR2 | No | 62.5 (alive) |
| 1121 | 23 | NK | *GATA2, TET2* | CR2 | 14.8 | CR1 | Yes | 17.4 (death) |
| 1368 | 52 | NK | NA | Refractory | 13.7 | - | - | 0.8 (death) |
| 1428 | 49 | NK | NA | CR2 | 15.1 | CR2 | No | 24.2 (death) |
| 1539 | 45 | del(9)(q22q34) | Nil | CR2 | 17.8 | CR1 | Yes | 10.4 (alive) |
| 1547 | 61 | NK | Nil | CR2 | 12.9 | CR2 | No | 11.4 (alive) |

Abbreviations: CR, complete remission; HSCT, hematopoietic stem cell transplantation; NA, not available; NK, normal karyotype; OS, overall survival; UPN, unique patient number

**Supplementary Table 7. Differential expressed genes between *CEBPA*^bZIP-inf^ with short EFS and long EFS.** There were 138 DEGs with |logFC| >0.585 (absolute fold change >1.5 or <0.67) and *P* value <0.05.

| genes | logFC | AveExpr | t | *P*.Value |
| --- | --- | --- | --- | --- |
| CAMP | -1.5900958 | 5.58545989 | -2.1521974 | 0.03790604 |
| AREG | -1.4822249 | 6.30871489 | -2.5146796 | 0.0163418 |
| CYP7B1 | -1.4530095 | 3.42327465 | -4.2963153 | 0.00011897 |
| CCL4L2 | -1.371224 | 2.41030229 | -2.3145507 | 0.02623222 |
| MDGA1 | -1.1868583 | 1.20499508 | -2.7653799 | 0.00878165 |
| RFPL4A | -1.1247343 | 0.3606589 | -2.7376382 | 0.00942014 |
| HCAR3 | -1.1088046 | 1.55198517 | -2.4447273 | 0.0193272 |
| TSPYL2 | -1.097513 | 4.75314146 | -3.0213734 | 0.00452242 |
| SPOCK2 | -1.0538277 | 2.70788243 | -2.8643977 | 0.0068164 |
| NR4A2 | -1.052223 | 4.89665908 | -2.4099568 | 0.02098859 |
| SHD | -1.0517734 | 4.55075571 | -2.4859433 | 0.01751319 |
| NEU4 | -1.0421428 | 3.05189368 | -2.2423247 | 0.0309557 |
| TNFAIP3 | -1.026109 | 5.754498 | -2.1212623 | 0.0405922 |
| CD83 | -1.0042545 | 4.00122242 | -2.1579748 | 0.03742217 |
| AK4 | -0.9849727 | 2.57434598 | -2.5215654 | 0.01607196 |
| CD8A | -0.9716206 | 2.37797346 | -2.6369712 | 0.01211605 |
| TP53INP2 | -0.9487714 | 2.75515147 | -2.7394434 | 0.00937731 |
| SLAIN1 | -0.945584 | 2.77240253 | -2.7037002 | 0.01025976 |
| TRH | -0.9366084 | 7.12657972 | -2.0432683 | 0.04812219 |
| GZMK | -0.9338609 | 1.7079837 | -2.874869 | 0.00663453 |
| GBP5 | -0.9138308 | 4.58877197 | -3.3957155 | 0.00163522 |
| VEGFA | -0.9028534 | 4.50670752 | -2.380599 | 0.02249101 |
| IL7R | -0.8930878 | 3.27004235 | -3.4632404 | 0.00135395 |
| MPO | -0.8918891 | 11.5244513 | -2.1882384 | 0.03497586 |
| C1orf21 | -0.8683116 | 2.41751953 | -2.8876462 | 0.00641876 |
| PLAU | -0.855787 | 4.14279658 | -3.7106786 | 0.00066984 |
| NEGR1 | -0.8462178 | 3.29118287 | -2.1503992 | 0.03805776 |
| CXCR4 | -0.844968 | 7.32310418 | -2.0481077 | 0.04762167 |
| RFPL4AL1 | -0.8362478 | -0.1138867 | -3.0617723 | 0.00406256 |
| OSM | -0.8231047 | 4.47916533 | -2.4373295 | 0.01967033 |
| SORCS1 | -0.8188038 | 1.92734692 | -2.4212021 | 0.02043761 |
| GPR84 | -0.8165522 | 1.1205286 | -2.1432644 | 0.03866508 |
| MAP1LC3A | -0.8160965 | 1.51862502 | -2.7941205 | 0.00816277 |
| CD8B | -0.8087304 | 1.59012401 | -2.6968484 | 0.01043747 |
| STON2 | -0.8062751 | 3.45272516 | -2.182161 | 0.03545541 |
| OAT | -0.7920367 | 5.14140862 | -2.8198443 | 0.00764352 |
| CD3E | -0.7908937 | 3.63608797 | -2.3868068 | 0.02216544 |
| ZBTB21 | -0.787106 | 4.34047036 | -2.1992992 | 0.03411786 |
| PER1 | -0.7856753 | 5.87051821 | -2.2547692 | 0.03009122 |
| NELL2 | -0.7737328 | 0.84704573 | -3.1873668 | 0.00289899 |
| ZNF667 | -0.7736429 | 2.64076053 | -2.7113069 | 0.01006576 |
| MRC2 | -0.7630546 | 3.62089153 | -2.1232036 | 0.04041882 |
| AC136428.1 | -0.7612303 | -0.0082191 | -2.829625 | 0.0074543 |
| ERFE | -0.7461427 | 0.88043862 | -2.2887075 | 0.02784234 |
| APBA1 | -0.7382909 | 2.1845576 | -2.0505287 | 0.04737299 |
| CD6 | -0.7379077 | 2.62560445 | -2.6838487 | 0.01078249 |
| COL6A2 | -0.7341267 | 0.7494233 | -2.8570227 | 0.00694727 |
| LIN7A | -0.7329578 | 0.40179022 | -2.4357575 | 0.01974395 |
| JMY | -0.7274792 | 1.8263087 | -2.8295349 | 0.00745603 |
| ZNF704 | -0.7159702 | 1.36956671 | -2.516141 | 0.01628419 |
| PDE4D | -0.7143973 | 4.49994668 | -2.3786695 | 0.02259308 |
| FAM110B | -0.7121466 | 0.05323991 | -3.8681161 | 0.00042424 |
| KRBA1 | -0.7044439 | 3.32465445 | -2.8385293 | 0.00728585 |
| BCL3 | -0.7039524 | 4.87840608 | -2.2034237 | 0.03380274 |
| DTX3 | -0.6939646 | 0.49085394 | -3.3784245 | 0.00171579 |
| CDH26 | -0.6939088 | 2.91726375 | -2.0429453 | 0.04815577 |
| TRIM46 | -0.6933789 | 1.83775921 | -2.0541546 | 0.04700267 |
| ICOS | -0.6909785 | 0.17432115 | -3.6851814 | 0.00072081 |
| RALGPS2 | -0.6769158 | 3.38513317 | -2.5820397 | 0.01387145 |
| TGFBR3 | -0.6749756 | 0.74206487 | -2.4016783 | 0.02140277 |
| DDIT3 | -0.6676399 | 6.33384969 | -2.1445196 | 0.03855762 |
| CNKSR2 | -0.6623723 | 0.6453708 | -2.3504206 | 0.02413616 |
| LAMA5 | -0.6620077 | 4.35925463 | -2.5982784 | 0.01332961 |
| CDK15 | -0.6575558 | 0.61535149 | -2.8153406 | 0.00773215 |
| MXD1 | -0.6540863 | 5.68979434 | -2.2168513 | 0.03279471 |
| FAAH | -0.6518985 | 4.45777016 | -2.043571 | 0.04809075 |
| ITK | -0.6508204 | 2.24323897 | -2.2509728 | 0.03035263 |
| ICAM1 | -0.6493784 | 3.85647223 | -2.1348167 | 0.03939522 |
| SIMC1 | -0.6451354 | 1.7436309 | -3.5934508 | 0.00093703 |
| SYCP2 | -0.6429004 | 0.37239874 | -2.0419772 | 0.04825651 |
| TPM2 | -0.6423211 | 2.61486446 | -2.7525419 | 0.00907195 |
| MSRB3 | -0.6400506 | 4.64903965 | -2.0689301 | 0.04551968 |
| FBXO44 | -0.6398573 | 2.2122927 | -2.6750103 | 0.01102305 |
| AC020915.4 | -0.6349468 | 1.4783072 | -2.9898296 | 0.00491512 |
| CD3G | -0.6347527 | 1.16349608 | -2.7394732 | 0.00937661 |
| DUSP8 | -0.6310825 | 1.17850856 | -2.7096475 | 0.01010779 |
| NR1D2 | -0.628001 | 3.45672651 | -2.3709578 | 0.02300523 |
| SFMBT1 | -0.6279099 | 2.2491975 | -2.9260401 | 0.00580927 |
| DCBLD2 | -0.6207194 | 0.38356469 | -2.9243748 | 0.00583454 |
| CTLA4 | -0.6184148 | 0.79533246 | -2.459918 | 0.01863962 |
| TIPARP | -0.6166855 | 4.96569718 | -2.4266986 | 0.02017311 |
| PLIN2 | -0.6148187 | 7.73316659 | -2.1291552 | 0.03989135 |
| SIDT1 | -0.6134639 | 4.3391868 | -2.3414566 | 0.02464532 |
| FAM53C | -0.6121486 | 5.47919817 | -2.3919398 | 0.02189946 |
| CAMK4 | -0.5883466 | 0.75329259 | -2.3245485 | 0.02563213 |
| ALDH7A1 | -0.5867595 | 2.30575986 | -2.4526595 | 0.01896534 |
| GFY | 0.5852158 | -0.616711 | 2.03944265 | 0.04852111 |
| PARVB | 0.58722382 | 4.94425045 | 2.97778332 | 0.00507339 |
| OAS2 | 0.59007498 | 4.29802207 | 2.22348791 | 0.03230644 |
| ADCY6 | 0.60567883 | 0.66779821 | 2.36574688 | 0.02328756 |
| CLEC3B | 0.61002777 | 1.82893987 | 2.65974542 | 0.01145024 |
| PAQR8 | 0.61084692 | 1.00159814 | 2.88850094 | 0.00640457 |
| SLC44A1 | 0.61500066 | 4.72942094 | 2.45305723 | 0.01894736 |
| CATSPER1 | 0.61929175 | 2.01409161 | 2.67693075 | 0.01097036 |
| CIBAR1 | 0.62223747 | 2.00277175 | 2.32947289 | 0.02534112 |
| PLCH1 | 0.62350498 | -0.019705 | 3.15296985 | 0.00318159 |
| LCN6 | 0.62893765 | -0.4844916 | 2.49501348 | 0.01713544 |
| KCNA6 | 0.63681001 | 0.2270122 | 2.16719899 | 0.03666096 |
| S100A1 | 0.65134812 | 1.44080391 | 2.52737033 | 0.01584765 |
| STARD8 | 0.65514694 | 0.97156703 | 2.13537218 | 0.03934684 |
| GPRC5C | 0.6597616 | 1.79839396 | 2.98613165 | 0.00496321 |
| SLC2A10 | 0.66416726 | 0.17334507 | 2.46413572 | 0.0184527 |
| TMEM14A | 0.67287489 | 1.29259899 | 2.3635092 | 0.02340975 |
| CLEC18B | 0.67457487 | 0.11910273 | 2.06956372 | 0.04545701 |
| GIMAP8 | 0.68349336 | 0.97772986 | 2.22539457 | 0.03216736 |
| NQO1 | 0.68718974 | 1.78981859 | 3.05585064 | 0.00412709 |
| PRKAR2B | 0.68783941 | 2.3949311 | 2.07051357 | 0.04536321 |
| LIG4 | 0.69001626 | 2.1034135 | 3.0705312 | 0.00396886 |
| MARVELD1 | 0.69775737 | 0.73899324 | 2.07464638 | 0.04495705 |
| TNFSF4 | 0.70562538 | 1.8400592 | 2.66291659 | 0.01136025 |
| PTH2 | 0.72042981 | -0.5745679 | 2.14351805 | 0.03864335 |
| CACNA2D4 | 0.72419496 | 3.10750808 | 2.10485027 | 0.04208437 |
| NACC2 | 0.73528814 | 1.18722771 | 2.75484559 | 0.00901921 |
| TP73 | 0.73782492 | 0.33196311 | 2.77992689 | 0.00846315 |
| LGALS1 | 0.74626093 | 9.33648096 | 2.72774567 | 0.00965805 |
| ASB9 | 0.75629554 | 2.70239143 | 2.58597372 | 0.01373835 |
| TUBB6 | 0.76437397 | 2.85579915 | 2.03206836 | 0.04929823 |
| KIAA1217 | 0.76700257 | 0.0165066 | 2.61645166 | 0.01274633 |
| FHL1 | 0.76989317 | 1.79724572 | 2.47465475 | 0.01799394 |
| EPS8L3 | 0.77461628 | -0.4829187 | 2.16922058 | 0.03649598 |
| RAB27B | 0.78296181 | 1.73609726 | 2.55187947 | 0.01493181 |
| NBL1 | 0.78501905 | 1.69940695 | 2.29992141 | 0.02713309 |
| HPSE | 0.80184693 | 1.10112626 | 2.09073453 | 0.04340618 |
| MAMDC2 | 0.84644664 | 1.09954204 | 2.24151979 | 0.03101237 |
| GIMAP6 | 0.88138707 | 3.65898461 | 2.69573533 | 0.01046661 |
| CCL1 | 0.88546632 | -0.2611978 | 2.20026239 | 0.03404404 |
| AP1S1 | 0.89628142 | 3.65692222 | 3.51754888 | 0.00116201 |
| MZB1 | 0.91327143 | 5.50382545 | 2.21559904 | 0.03288757 |
| GPM6A | 0.94975299 | 0.0248381 | 2.97071791 | 0.00516844 |
| SMPDL3B | 0.98713746 | 2.52769056 | 4.34039622 | 0.00010416 |
| TNNI3 | 1.00764397 | 0.52920709 | 2.39581286 | 0.02170069 |
| EMP1 | 1.02181282 | 3.46960678 | 2.29298605 | 0.02756979 |
| TNNT1 | 1.02540044 | 0.29994183 | 3.00729073 | 0.00469394 |
| CD109 | 1.02974348 | 0.64751873 | 2.81875135 | 0.00766494 |
| SERPINE1 | 1.08843295 | 1.8857037 | 2.93579763 | 0.00566326 |
| ANXA2 | 1.10364886 | 5.79338302 | 2.51264804 | 0.0164222 |
| IGFBP2 | 1.10853575 | 1.55293701 | 2.34217966 | 0.0246039 |
| DNAAF3 | 1.34781922 | 1.45968994 | 2.61112782 | 0.01291472 |

**Supplementary Table 8. Comparison of clinical and laboratory features between patients with *CEBPA*^bZIP-inf^ with high and low expression of *ATP5F1C***

|  | **Low *ATP5F1C***  **(n=16)** | **High *ATP5F1C***  **(n=20)** | ***P* value** |
| --- | --- | --- | --- |
| **Age (year)** | 34.8 (18-57) | 36 (19-66) | 0.479 |
| **Lab data** |  |  |  |
| WBC (k/μL) | 16.6 (4.49-148.3) | 46.0 (4.27-387.4) | 0.117 |
| Hb (g/dL) | 8.75 (5.5-15) | 9.2 (5.7-12.6) | 0.857 |
| Platelet (k/μL) | 31 (8-112) | 43 (5-185) | 0.545 |
| PB Blast (%) | 68 (36-96) | 79 (17-96) | 0.385 |
| LDH (U/L) | 720 (230-1868) | 881 (293-7747) | 0.172 |
| **Normal karyotype** | 14 (87.5) | 18 (90) | >0.999 |
| **Mutations** |  |  |  |
| *GATA2* | 3 (23.1) | 4 (20) | >0.999 |
| *WT1* | 1 (7.7) | 3 (17.6) | 0.613 |
| *NRAS* | 2 (14.3) | 5 (25) | 0.672 |
| *FLT3-*ITD | 1 (6.7) | 1 (5.0) | >0.999 |
| *TET2* | 1 (6.7) | 2 (11.1) | >0.999 |
| *DNMT3A* | 1 (6.3) | 1 (5.3) | >0.999 |
| *ASXL1* | 1 (6.3) | 1 (5.3) | >0.999 |
| *RAD21* | 1 (6.7) | 0 (0) | 0.429 |
| *KIT* | 0 (0) | 1 (5.0) | >0.999 |

Abbreviation: Hb, hemoglobin; LDH, lactate dehydrogenase; PB, peripheral blood; WBC, white blood cell.
