# Supplementary material for: Dysregulated immune and metabolic pathways are associated with poor survival in adult acute myeloid leukemia with CEBPA bZIP in-frame mutations
Source: Blood Cancer J. 2024 Jan 23;14(1):15. doi: 10.1038/s41408-023-00975-8 (PMC10803338; doi:10.1038/s41408-023-00975-8)
Supplement: Supplementary file 2 — Supplementary table 6 [file 41408_2023_975_MOESM2_ESM.docx]

**Supplementary Table 6. Differential expressed genes between *CEBPA*^bZIP-inf^ and *CEBPA*^wt^.** There were 1176 DEGs with |logFC| >1 and *P* value <0.01.

| genes | logFC | AveExpr | t | *P*.Value |
| --- | --- | --- | --- | --- |
| HOXA9 | -5.9147585 | 3.81710976 | -12.751974 | 1.8165E-24 |
| HOXA5 | -4.9748551 | 3.10472217 | -11.449092 | 2.8466E-21 |
| HOXA10 | -4.6267128 | 3.09375634 | -12.572746 | 4.9752E-24 |
| HOXB3 | -4.5532266 | 3.21729857 | -7.8954988 | 1.1923E-12 |
| CPNE8 | -4.3575815 | 2.85881909 | -13.575181 | 1.8248E-26 |
| CLU | -4.2525232 | 5.09974545 | -7.9851973 | 7.3579E-13 |
| RAB34 | -4.2026111 | 4.13146796 | -9.7125964 | 5.2649E-17 |
| MEIS1 | -4.1385276 | 3.05727457 | -9.8006331 | 3.2083E-17 |
| HOXA7 | -4.1245062 | 2.25347951 | -13.223991 | 1.2913E-25 |
| HOXA3 | -4.0795223 | 2.23830213 | -13.131589 | 2.1642E-25 |
| HOXA6 | -3.8828749 | 2.25712187 | -12.273589 | 2.6844E-23 |
| HOXB6 | -3.8477377 | 2.07104904 | -9.5961019 | 1.0131E-16 |
| SELENOP | -3.7306419 | 3.31573088 | -6.2639447 | 5.3767E-09 |
| IFITM3 | -3.4508393 | 5.22093809 | -7.5112419 | 9.2412E-12 |
| BEX1 | -3.3367921 | 3.23317835 | -5.3725172 | 3.5933E-07 |
| HOXB4 | -3.3219939 | 1.8850954 | -10.466769 | 7.4553E-19 |
| BASP1 | -3.2850803 | 3.38538062 | -7.2649182 | 3.3701E-11 |
| JAG1 | -3.2802754 | 3.43561055 | -7.9991326 | 6.8253E-13 |
| SCHIP1 | -3.2601373 | 2.38906913 | -8.1611145 | 2.8417E-13 |
| RTL8C | -3.1960258 | 2.45450606 | -9.7419566 | 4.4635E-17 |
| OBSL1 | -3.1234884 | 2.89438012 | -7.9973219 | 6.8922E-13 |
| HOXB2 | -3.1048969 | 2.20108736 | -8.5739504 | 2.9809E-14 |
| DEFB1 | -3.1031269 | 2.94139839 | -4.4831813 | 1.6273E-05 |
| NPDC1 | -3.0747231 | 2.91142367 | -7.0903896 | 8.346E-11 |
| F13A1 | -3.071357 | 4.66699957 | -5.7839034 | 5.391E-08 |
| TNS3 | -3.0470154 | 2.95597098 | -10.213157 | 3.1301E-18 |
| H1-0 | -3.0271611 | 5.21604875 | -6.9472329 | 1.7446E-10 |
| NAALADL1 | -3.0165156 | 3.09545958 | -8.868669 | 5.8638E-15 |
| CFD | -3.0151907 | 6.2270203 | -7.1271469 | 6.8999E-11 |
| SUCLG2 | -2.9937047 | 2.73994962 | -9.1788654 | 1.0461E-15 |
| ZNF521 | -2.9717684 | 1.49462453 | -8.558317 | 3.2483E-14 |
| HOXA4 | -2.9600028 | 1.79209936 | -11.217697 | 1.0568E-20 |
| RUSC2 | -2.9274134 | 2.00613646 | -13.857581 | 3.8101E-27 |
| NKX2-3 | -2.9101616 | 1.30220011 | -10.930156 | 5.3963E-20 |
| EREG | -2.9062627 | 3.39390093 | -5.2403131 | 6.5004E-07 |
| AZU1 | -2.8976661 | 8.53440893 | -7.321963 | 2.501E-11 |
| MARVELD1 | -2.8081799 | 2.6511564 | -10.519353 | 5.5355E-19 |
| KRT18 | -2.8081524 | 1.99790121 | -7.9042312 | 1.1377E-12 |
| HOXB5 | -2.8071649 | 1.22166241 | -10.494411 | 6.3752E-19 |
| C3orf80 | -2.792141 | 2.27150249 | -7.3718426 | 1.9256E-11 |
| MAP3K20 | -2.7866363 | 2.38325803 | -10.679744 | 2.2313E-19 |
| RNF217 | -2.7828384 | 2.76219059 | -8.9405511 | 3.9369E-15 |
| SEL1L3 | -2.7633778 | 3.58371471 | -10.075729 | 6.8031E-18 |
| SLITRK4 | -2.6995382 | 1.32781577 | -12.475091 | 8.6209E-24 |
| NDFIP1 | -2.6763396 | 3.96361064 | -9.0275411 | 2.4288E-15 |
| RAB13 | -2.6623959 | 4.64158312 | -8.5891616 | 2.7418E-14 |
| PRTN3 | -2.6487888 | 6.38796261 | -5.7195582 | 7.2896E-08 |
| MMP2 | -2.6424732 | 2.45552656 | -5.8614394 | 3.7389E-08 |
| LAMB2 | -2.6410869 | 2.17895477 | -6.6397258 | 8.3216E-10 |
| EPB41L2 | -2.6317743 | 2.67411441 | -7.3585226 | 2.065E-11 |
| RTL8A | -2.6305836 | 2.26444838 | -8.8683809 | 5.8731E-15 |
| GPR162 | -2.6298022 | 2.82131283 | -7.6951099 | 3.4835E-12 |
| SLC22A15 | -2.6217422 | 3.67776284 | -6.8774187 | 2.4939E-10 |
| GABBR1 | -2.6172458 | 3.07084387 | -5.7088819 | 7.6625E-08 |
| ECHDC2 | -2.6076241 | 4.1144261 | -8.0199016 | 6.1017E-13 |
| ARHGAP21 | -2.5950357 | 2.68231322 | -9.6589427 | 7.1183E-17 |
| CYP27A1 | -2.5718298 | 1.26509943 | -8.498649 | 4.507E-14 |
| ATN1 | -2.5677321 | 3.51834702 | -11.440538 | 2.988E-21 |
| CES1 | -2.5599474 | 1.67457859 | -6.4958359 | 1.7102E-09 |
| PHGDH | -2.5571669 | 3.01363328 | -8.2127072 | 2.1474E-13 |
| CTSG | -2.5333837 | 8.30980075 | -6.5458121 | 1.3327E-09 |
| RHPN1 | -2.5269733 | 3.45753269 | -8.1985135 | 2.3195E-13 |
| HNMT | -2.5032498 | 2.67949819 | -5.7906457 | 5.2226E-08 |
| DACH1 | -2.4831283 | 1.16000729 | -14.004535 | 1.6908E-27 |
| ACP6 | -2.4694276 | 2.55021187 | -9.493397 | 1.8026E-16 |
| PCCA | -2.4637795 | 2.53263985 | -13.446261 | 3.7385E-26 |
| KIF16B | -2.4558872 | 1.37113827 | -13.207711 | 1.4142E-25 |
| CA1 | -2.4462592 | 5.98217775 | -4.4230211 | 2.0733E-05 |
| GRAMD1B | -2.4334014 | 1.6644215 | -12.974 | 5.2282E-25 |
| PLPPR3 | -2.4221729 | 2.80785127 | -6.8072595 | 3.5659E-10 |
| DEFA1B | -2.411688 | 9.42232415 | -4.4937886 | 1.5589E-05 |
| IGFBP2 | -2.4021124 | 3.18768314 | -4.8337423 | 3.8075E-06 |
| DSC2 | -2.3772167 | 0.9347684 | -10.622479 | 3.0865E-19 |
| F3 | -2.3767421 | 1.06028251 | -13.044816 | 3.5166E-25 |
| CD9 | -2.3645999 | 3.73941937 | -5.971586 | 2.2135E-08 |
| LIN7A | -2.3602818 | 2.03527396 | -6.5936731 | 1.0487E-09 |
| RNASE3 | -2.3436301 | 6.56296483 | -5.7033596 | 7.8626E-08 |
| ELANE | -2.3059921 | 7.99183672 | -5.6297217 | 1.1075E-07 |
| DEFA3 | -2.2773576 | 8.76279086 | -4.4510448 | 1.8526E-05 |
| SGMS2 | -2.2313718 | 2.15989741 | -6.6710665 | 7.1066E-10 |
| CLC | -2.2306866 | 6.15263903 | -4.1016968 | 7.287E-05 |
| DOCK1 | -2.2253728 | 0.9320821 | -8.4895892 | 4.7365E-14 |
| MTARC1 | -2.2049491 | 3.26269668 | -5.6006157 | 1.2672E-07 |
| DPYSL3 | -2.2011617 | 1.19095578 | -9.0951801 | 1.6673E-15 |
| TRIB1 | -2.1936552 | 5.68486897 | -6.53635 | 1.3973E-09 |
| TLR4 | -2.1923193 | 3.67551064 | -5.3383113 | 4.1924E-07 |
| ANXA5 | -2.1920859 | 4.88095587 | -4.8139733 | 4.14E-06 |
| CRISP3 | -2.1864688 | 1.50569427 | -5.785403 | 5.3531E-08 |
| LTBP4 | -2.1861778 | 3.7085528 | -7.9760462 | 7.7299E-13 |
| FGFRL1 | -2.1858243 | 3.09535319 | -10.147905 | 4.5257E-18 |
| MBOAT2 | -2.1747652 | 3.33315026 | -5.4704232 | 2.3041E-07 |
| SYTL4 | -2.1698822 | 0.98017627 | -9.7341565 | 4.6637E-17 |
| RNASE2 | -2.1693873 | 8.12401688 | -5.8765876 | 3.4798E-08 |
| SLC16A1 | -2.1659817 | 1.48892479 | -11.215824 | 1.0681E-20 |
| TUBB6 | -2.1531324 | 4.32027199 | -6.1821856 | 8.0138E-09 |
| MT1F | -2.1455283 | 2.48249089 | -7.4288771 | 1.4268E-11 |
| SMCO4 | -2.1345825 | 3.24368042 | -6.6174549 | 9.3074E-10 |
| SPATS2L | -2.1312385 | 2.06926633 | -8.9844048 | 3.0865E-15 |
| DEPP1 | -2.130418 | 1.99593279 | -8.050256 | 5.1791E-13 |
| PBX3 | -2.1300185 | 4.69414627 | -6.2346957 | 6.2037E-09 |
| TM4SF1 | -2.1282461 | 0.99206964 | -7.5786285 | 6.4691E-12 |
| METTL7B | -2.1280918 | 1.29356119 | -7.5705178 | 6.7533E-12 |
| ZNF711 | -2.1153419 | 0.91343527 | -7.5815904 | 6.3684E-12 |
| PHETA2 | -2.1075279 | 1.49682489 | -11.080556 | 2.3E-20 |
| CCN3 | -2.0901287 | 0.94646316 | -8.4719757 | 5.2166E-14 |
| HOXB7 | -2.0896907 | 0.77614158 | -9.9071317 | 1.761E-17 |
| CD4 | -2.0726401 | 4.60783059 | -5.4640556 | 2.372E-07 |
| CACNB3 | -2.0705195 | 2.2693494 | -9.9773525 | 1.1852E-17 |
| SARM1 | -2.0696613 | 2.26258836 | -11.116004 | 1.8812E-20 |
| ARHGAP32 | -2.0661805 | 1.07737497 | -12.135863 | 5.8408E-23 |
| ANKRD18A | -2.061957 | 1.70967702 | -6.8774703 | 2.4932E-10 |
| ARHGAP22 | -2.0545301 | 2.86495938 | -7.2351276 | 3.9366E-11 |
| PTGDS | -2.0516313 | 3.01759283 | -5.4681232 | 2.3284E-07 |
| IRX3 | -2.0483675 | 0.70339465 | -8.1394167 | 3.1965E-13 |
| ADGRA2 | -2.0412516 | 1.66584535 | -7.4589842 | 1.2176E-11 |
| ST8SIA6 | -2.0257515 | 1.89801783 | -7.4411905 | 1.3373E-11 |
| PCOLCE2 | -2.0119336 | 1.09210992 | -7.7039408 | 3.3233E-12 |
| ALDH2 | -2.0060909 | 3.67852988 | -5.0284062 | 1.6509E-06 |
| KLF4 | -1.99694 | 4.11822756 | -3.8683438 | 0.00017438 |
| LTBP1 | -1.9932118 | 2.1324552 | -4.9838606 | 2.0023E-06 |
| MACROH2A2 | -1.9930726 | 1.51376776 | -7.1398686 | 6.4595E-11 |
| SHTN1 | -1.990276 | 1.59830952 | -6.1024561 | 1.1797E-08 |
| SFXN3 | -1.9868515 | 4.60629341 | -7.5765757 | 6.5399E-12 |
| IGF2R | -1.9851553 | 3.46329789 | -5.9826226 | 2.0997E-08 |
| TNFAIP2 | -1.9717524 | 6.46038788 | -6.0496192 | 1.5222E-08 |
| CYSTM1 | -1.9687868 | 4.51996891 | -6.0967148 | 1.2129E-08 |
| TCEAL9 | -1.9661654 | 2.49367523 | -6.2289512 | 6.3802E-09 |
| FCHSD1 | -1.9618609 | 4.42804593 | -9.3963833 | 3.1037E-16 |
| TMTC2 | -1.9598719 | 2.24281741 | -7.1072126 | 7.6503E-11 |
| NT5C3B | -1.9561041 | 3.10089743 | -9.7024885 | 5.5728E-17 |
| MMP19 | -1.9522651 | 1.17223563 | -7.9037198 | 1.1408E-12 |
| AL445423.3 | -1.9511036 | 1.5185047 | -7.8487025 | 1.5327E-12 |
| CPED1 | -1.9477603 | 0.87883734 | -8.9113157 | 4.6298E-15 |
| CCL3L3 | -1.9363709 | 4.89217663 | -3.7444926 | 0.00027316 |
| VNN1 | -1.9298804 | 1.9387861 | -5.7520619 | 6.2605E-08 |
| ABI2 | -1.9237787 | 2.72037189 | -9.2673879 | 6.3838E-16 |
| TDRD9 | -1.9236519 | 1.20852936 | -8.2145399 | 2.1261E-13 |
| BCL6 | -1.9094612 | 5.30061052 | -5.6707638 | 9.1526E-08 |
| SORBS3 | -1.8952884 | 3.26417851 | -6.634912 | 8.5256E-10 |
| HOXB9 | -1.8951448 | 0.45387213 | -6.9808049 | 1.4684E-10 |
| MMP17 | -1.8949328 | 2.17150811 | -6.355177 | 3.4341E-09 |
| LDLRAD3 | -1.8880445 | 1.26803008 | -7.421992 | 1.4795E-11 |
| HBM | -1.8853087 | 3.59768149 | -3.2030074 | 0.00172041 |
| TP53INP2 | -1.8797868 | 4.00430529 | -5.4886656 | 2.1199E-07 |
| HOXB8 | -1.8777543 | 0.43348148 | -7.2590507 | 3.4749E-11 |
| ITGAV | -1.8763076 | 1.50671616 | -9.3715221 | 3.567E-16 |
| TTC12 | -1.8666862 | 2.21367838 | -6.9727922 | 1.5301E-10 |
| HK3 | -1.8634768 | 5.37065228 | -4.0378682 | 9.2832E-05 |
| CAPN2 | -1.8543456 | 5.30901997 | -6.9851884 | 1.4356E-10 |
| TSPO | -1.8526598 | 7.45726264 | -8.5689743 | 3.0636E-14 |
| FAM20C | -1.8496932 | 2.36549978 | -5.1199978 | 1.1066E-06 |
| TRIB3 | -1.847031 | 2.28142751 | -9.3017465 | 5.269E-16 |
| VAV2 | -1.845179 | 1.72361512 | -9.3389497 | 4.2797E-16 |
| SLC2A8 | -1.8359473 | 1.50095778 | -9.4148361 | 2.7991E-16 |
| CDKN1A | -1.8355668 | 4.71731658 | -5.0906858 | 1.2583E-06 |
| DLG4 | -1.8320955 | 3.60569799 | -8.1604709 | 2.8516E-13 |
| ANK3 | -1.8308778 | 1.08517124 | -8.2756075 | 1.5251E-13 |
| ZNF503 | -1.8307353 | 0.90049832 | -9.081954 | 1.7947E-15 |
| AP5B1 | -1.8268489 | 4.54052435 | -8.5200927 | 4.0068E-14 |
| MMEL1 | -1.8224834 | 1.18487486 | -6.7514423 | 4.734E-10 |
| ICA1 | -1.8205377 | 3.40725419 | -6.4029729 | 2.712E-09 |
| RHBDF1 | -1.8015799 | 1.04696463 | -9.3573064 | 3.8622E-16 |
| PSRC1 | -1.7987547 | 1.87366052 | -9.0624148 | 2.0008E-15 |
| C16orf74 | -1.796615 | 3.53210867 | -6.943108 | 1.7819E-10 |
| PPDPF | -1.7942478 | 5.59414324 | -10.573268 | 4.0789E-19 |
| TREM1 | -1.7942102 | 5.67994393 | -4.1578693 | 5.8762E-05 |
| VNN2 | -1.7913298 | 4.41829401 | -4.0696216 | 8.2325E-05 |
| ODF3B | -1.7776425 | 4.70238473 | -5.2883982 | 5.2449E-07 |
| SNX24 | -1.7755878 | 1.87093825 | -8.2090487 | 2.1905E-13 |
| BIK | -1.7754837 | 2.02606384 | -7.0147526 | 1.2331E-10 |
| GBP2 | -1.7726516 | 4.86957085 | -7.3636858 | 2.0098E-11 |
| SERPINH1 | -1.7704022 | 2.20046838 | -7.2198653 | 4.2624E-11 |
| CYP1B1 | -1.7699418 | 0.9531324 | -6.3362345 | 3.7701E-09 |
| SPRED2 | -1.7694205 | 1.52640645 | -10.180225 | 3.7703E-18 |
| MTARC2 | -1.7663352 | 1.29238546 | -5.742573 | 6.5452E-08 |
| UGGT2 | -1.7628896 | 1.67509015 | -7.3322976 | 2.3693E-11 |
| CT45A5 | -1.7604557 | 0.46088992 | -6.4616396 | 2.0274E-09 |
| NRBP2 | -1.7581841 | 1.39497908 | -10.101949 | 5.8669E-18 |
| HAL | -1.7580145 | 3.66503632 | -4.9117517 | 2.7306E-06 |
| ROBO3 | -1.7579321 | 2.81296103 | -4.5808327 | 1.0934E-05 |
| KCNQ1 | -1.7561071 | 2.5792223 | -5.5131857 | 1.8949E-07 |
| ENO2 | -1.7550415 | 2.33157059 | -8.1107669 | 3.7333E-13 |
| JAZF1 | -1.7483583 | 2.61971012 | -5.8305634 | 4.3267E-08 |
| KLF9 | -1.7476568 | 2.19933595 | -5.7521758 | 6.2571E-08 |
| PHLDA1 | -1.7471477 | 1.55092732 | -6.7270149 | 5.3573E-10 |
| CLIC2 | -1.7425147 | 1.99062262 | -5.2592713 | 5.9738E-07 |
| P3H3 | -1.7392256 | 0.79734841 | -8.7276346 | 1.2787E-14 |
| LYST | -1.7381978 | 5.32769355 | -6.9989066 | 1.3379E-10 |
| FOXC1 | -1.7343061 | 0.95369643 | -5.3160735 | 4.633E-07 |
| MAMDC2 | -1.7303242 | 2.23535883 | -5.1054609 | 1.1794E-06 |
| S100A10 | -1.7234585 | 7.07414524 | -4.6509917 | 8.1892E-06 |
| FXYD6 | -1.7174702 | 2.32236748 | -4.9682808 | 2.1416E-06 |
| TNFSF13B | -1.716264 | 4.65008665 | -6.1585696 | 8.9887E-09 |
| CACNB1 | -1.7133284 | 1.95242628 | -8.0121579 | 6.3621E-13 |
| THBS1 | -1.7130449 | 2.61747244 | -3.4082499 | 0.00087716 |
| RIN2 | -1.7072169 | 1.92558263 | -6.4960195 | 1.7086E-09 |
| MNDA | -1.7065887 | 6.15461988 | -4.7688877 | 5.0071E-06 |
| COL4A5 | -1.7049827 | 2.20943715 | -3.7099849 | 0.00030899 |
| ADAMTSL4 | -1.7038727 | 4.2213889 | -5.4142157 | 2.9754E-07 |
| PLAUR | -1.701863 | 6.12318867 | -5.4079542 | 3.061E-07 |
| MTA3 | -1.7003293 | 1.87152889 | -8.7507311 | 1.1257E-14 |
| SLC2A5 | -1.6979228 | 3.85735624 | -6.4752231 | 1.895E-09 |
| FHL1 | -1.6946396 | 2.93368178 | -5.5451116 | 1.6366E-07 |
| COPZ2 | -1.6946115 | 0.8791026 | -8.9656041 | 3.426E-15 |
| SETD7 | -1.6928138 | 2.50398516 | -7.5654244 | 6.938E-12 |
| PTMS | -1.6926988 | 4.21723021 | -5.9224736 | 2.7981E-08 |
| SH2B2 | -1.6925986 | 1.98180326 | -8.2535227 | 1.7199E-13 |
| NINJ1 | -1.6917098 | 5.37698084 | -5.6777667 | 8.859E-08 |
| SIRPA | -1.6906782 | 4.31020363 | -5.4228907 | 2.8605E-07 |
| DUSP6 | -1.6877269 | 6.11003612 | -5.8171422 | 4.6097E-08 |
| TMEM176A | -1.6874539 | 2.93703783 | -3.3681274 | 0.00100296 |
| H2AJ | -1.6861306 | 5.12538607 | -6.3050772 | 4.3944E-09 |
| NOXA1 | -1.6830991 | 2.5243103 | -8.9820803 | 3.1266E-15 |
| TYMP | -1.6786393 | 5.84747376 | -4.3716287 | 2.5456E-05 |
| NUDT14 | -1.6764206 | 2.51572501 | -8.9532602 | 3.6689E-15 |
| LONRF3 | -1.667261 | 1.0928068 | -9.1072525 | 1.559E-15 |
| GASK1B | -1.6602359 | 2.54763918 | -4.4309115 | 2.0087E-05 |
| EPS8L1 | -1.6596107 | 1.25738935 | -9.1852186 | 1.0097E-15 |
| IRS2 | -1.6592795 | 3.28145543 | -6.2837103 | 4.8803E-09 |
| SDSL | -1.658689 | 3.08128893 | -7.1418675 | 6.3929E-11 |
| CCSER1 | -1.6583511 | 1.28525554 | -7.415731 | 1.529E-11 |
| IFIT1B | -1.6534813 | 1.50100156 | -3.9545245 | 0.00012686 |
| LRG1 | -1.6529786 | 3.03337789 | -5.3637131 | 3.739E-07 |
| SERINC2 | -1.6519411 | 1.21717449 | -6.6934566 | 6.3475E-10 |
| CT45A3 | -1.6489436 | 0.44890835 | -5.0455165 | 1.5325E-06 |
| FAM95C | -1.6434699 | 0.9750069 | -7.4220634 | 1.4789E-11 |
| PRICKLE1 | -1.641929 | 2.1599779 | -6.4483265 | 2.166E-09 |
| GDPD5 | -1.6383285 | 3.11237966 | -6.7495592 | 4.7794E-10 |
| MFSD2A | -1.6365478 | 1.82024806 | -7.304359 | 2.7424E-11 |
| MELTF | -1.636031 | 1.95555389 | -8.2016889 | 2.2799E-13 |
| CLCF1 | -1.6351778 | 1.83540408 | -8.0557362 | 5.028E-13 |
| SMIM10 | -1.6334788 | 1.76726818 | -6.971759 | 1.5382E-10 |
| ADM | -1.6334485 | 2.39371502 | -4.4765288 | 1.6716E-05 |
| CX3CR1 | -1.6325364 | 2.2338841 | -4.0108037 | 0.00010279 |
| TP53I11 | -1.6319737 | 3.20434469 | -5.1862187 | 8.2643E-07 |
| RGCC | -1.6306865 | 7.37688896 | -5.6437879 | 1.0375E-07 |
| CLIP4 | -1.6290681 | 3.38218733 | -7.4236008 | 1.467E-11 |
| JUN | -1.625891 | 7.53605027 | -4.9866283 | 1.9785E-06 |
| EXT1 | -1.6197491 | 0.81735566 | -8.7708314 | 1.0074E-14 |
| BMPR2 | -1.6184795 | 0.98641112 | -9.4618826 | 2.1508E-16 |
| PRKCZ | -1.6151179 | 1.44000531 | -9.5705576 | 1.1693E-16 |
| DMXL2 | -1.6139576 | 5.32976341 | -5.3044689 | 4.8806E-07 |
| ABCC6 | -1.6134638 | 1.16313445 | -8.9728903 | 3.2902E-15 |
| RBPMS | -1.6127989 | 2.09420194 | -5.4793715 | 2.2119E-07 |
| PLD2 | -1.6083348 | 2.69349518 | -5.9689881 | 2.2412E-08 |
| PLAAT3 | -1.6054196 | 1.91509613 | -5.2476633 | 6.2911E-07 |
| PI4K2A | -1.6022178 | 1.84449913 | -6.7929314 | 3.8353E-10 |
| BEX2 | -1.5963457 | 1.40983158 | -5.7117267 | 7.5613E-08 |
| FRMD3 | -1.5939197 | 1.51276134 | -8.1454448 | 3.0937E-13 |
| HOMER3 | -1.5922525 | 5.80667082 | -5.4974266 | 2.0367E-07 |
| MPP6 | -1.5903259 | 0.9975442 | -8.418607 | 6.987E-14 |
| BEX3 | -1.5879765 | 3.89623339 | -3.8606102 | 0.00017939 |
| MMP8 | -1.586095 | 5.02647954 | -3.3158434 | 0.00119232 |
| CHIC1 | -1.5860252 | 1.10261845 | -11.945491 | 1.7128E-22 |
| TCN2 | -1.5832272 | 2.8453518 | -4.7842074 | 4.6944E-06 |
| CTSL | -1.5830064 | 3.55977642 | -3.5895456 | 0.00047212 |
| H2AW | -1.5769346 | 0.91803074 | -5.7210365 | 7.2394E-08 |
| TUBA4A | -1.5767885 | 4.8296026 | -5.1844818 | 8.3281E-07 |
| DPPA4 | -1.5767208 | 0.80885945 | -5.0947924 | 1.2359E-06 |
| KCTD17 | -1.5734282 | 2.14364158 | -7.35116 | 2.1463E-11 |
| CT45A10 | -1.5716045 | 0.21411218 | -5.4144109 | 2.9727E-07 |
| CTNNA1 | -1.5697702 | 5.43198804 | -8.5269736 | 3.8583E-14 |
| SCRN1 | -1.5669292 | 1.7168896 | -4.3191237 | 3.1343E-05 |
| SERPINB10 | -1.5664485 | 3.34424665 | -4.1003434 | 7.3247E-05 |
| WWC3 | -1.5637839 | 2.77760479 | -7.7930387 | 2.0651E-12 |
| CRYBG3 | -1.5624155 | 1.34198366 | -6.9213359 | 1.9922E-10 |
| MPP7 | -1.5607232 | 2.74683967 | -6.7172231 | 5.6293E-10 |
| HTRA3 | -1.5543368 | 2.11486597 | -5.1847041 | 8.3199E-07 |
| TMEM14A | -1.5538092 | 2.3169541 | -8.4913572 | 4.6908E-14 |
| HSPB1 | -1.5534021 | 5.21760886 | -7.483715 | 1.0687E-11 |
| ID1 | -1.5533515 | 1.5316309 | -6.1897924 | 7.7226E-09 |
| IL4R | -1.5533036 | 4.80207603 | -7.0160084 | 1.2251E-10 |
| SORT1 | -1.5515581 | 3.06611079 | -5.1422421 | 1.0035E-06 |
| ITGA7 | -1.5494044 | 2.23361856 | -5.345902 | 4.0516E-07 |
| AATK | -1.5489948 | 1.53228232 | -8.0777295 | 4.4642E-13 |
| HOXA11 | -1.547819 | 0.25226656 | -7.1483053 | 6.183E-11 |
| SLC36A4 | -1.5453222 | 3.81391192 | -6.7314378 | 5.2387E-10 |
| NR6A1 | -1.543257 | 0.99670552 | -9.8221362 | 2.8425E-17 |
| PTX3 | -1.538627 | 3.04181528 | -4.5660655 | 1.1616E-05 |
| KRT23 | -1.5368626 | 0.78757076 | -5.8084488 | 4.8026E-08 |
| ADGRB1 | -1.5361512 | 0.68487365 | -8.5931416 | 2.6825E-14 |
| GNPDA1 | -1.5355404 | 3.02226145 | -5.2142658 | 7.2984E-07 |
| KBTBD11 | -1.5347735 | 2.38474811 | -7.098491 | 8.0035E-11 |
| CKAP4 | -1.5340403 | 3.77145368 | -4.3899604 | 2.3663E-05 |
| CPVL | -1.5335593 | 4.75532354 | -3.7033266 | 0.0003164 |
| ASAP2 | -1.5319424 | 0.69587768 | -9.0515141 | 2.1258E-15 |
| HCAR2 | -1.5259335 | 2.38253757 | -4.5527699 | 1.2264E-05 |
| CDK18 | -1.5239489 | 1.8597254 | -9.571397 | 1.1638E-16 |
| GRN | -1.521818 | 8.00038297 | -5.9677494 | 2.2545E-08 |
| CYSLTR2 | -1.5207451 | 2.36419947 | -5.4512733 | 2.5142E-07 |
| KLHL17 | -1.5194589 | 2.60337309 | -10.364286 | 1.3316E-18 |
| ADGRE1 | -1.5159254 | 4.04656195 | -5.4655774 | 2.3556E-07 |
| TNFSF12 | -1.5143655 | 2.96727362 | -4.5989523 | 1.015E-05 |
| HES1 | -1.5141962 | 0.74809755 | -6.2533426 | 5.6631E-09 |
| ADCY9 | -1.5119349 | 1.62278585 | -6.0120442 | 1.8235E-08 |
| CCL3 | -1.5109849 | 5.68691386 | -3.2957294 | 0.0012737 |
| LGALS3 | -1.5084714 | 5.73051188 | -4.1312664 | 6.5082E-05 |
| DST | -1.5083788 | 2.02585864 | -5.3515205 | 3.9503E-07 |
| CTTNBP2NL | -1.507114 | 0.63687878 | -10.871575 | 7.5221E-20 |
| KLHDC8B | -1.5041203 | 2.79318105 | -6.7750275 | 4.2003E-10 |
| AC072022.2 | -1.5040722 | 1.56098664 | -6.5234045 | 1.4906E-09 |
| RELL2 | -1.5035564 | 2.00829268 | -7.4569476 | 1.2307E-11 |
| IQSEC2 | -1.5029211 | 2.6619685 | -5.8902867 | 3.2608E-08 |
| PCBP4 | -1.5008808 | 1.675444 | -8.1345236 | 3.2824E-13 |
| VDR | -1.5008671 | 1.58609534 | -6.0212431 | 1.7448E-08 |
| ZNF334 | -1.499376 | 0.60758986 | -9.0547911 | 2.0874E-15 |
| AQP3 | -1.4974461 | 2.67035986 | -5.6065612 | 1.2328E-07 |
| AIF1L | -1.496936 | 1.59897582 | -4.2516437 | 4.0851E-05 |
| SNX10 | -1.4943202 | 4.04541042 | -5.8503982 | 3.9395E-08 |
| CSTA | -1.4935572 | 6.72821283 | -4.3433707 | 2.8477E-05 |
| SHISA4 | -1.4915201 | 0.56432431 | -8.2253617 | 2.0046E-13 |
| SERPINB2 | -1.4908274 | 2.94397109 | -3.1300585 | 0.00217009 |
| MT1E | -1.4901489 | 1.17341077 | -5.0787006 | 1.326E-06 |
| KRT8 | -1.4896958 | 2.14144961 | -5.8139776 | 4.679E-08 |
| VNN3 | -1.4881108 | 2.32972622 | -3.9356571 | 0.00013606 |
| TRIM7 | -1.4880499 | 0.95606648 | -6.6169228 | 9.3323E-10 |
| PRDM16 | -1.4879394 | 0.85983997 | -6.0832128 | 1.2947E-08 |
| SLC8A1 | -1.4837329 | 1.4161506 | -4.8787011 | 3.1448E-06 |
| C2 | -1.4834594 | 2.14615883 | -3.6346022 | 0.00040334 |
| VLDLR | -1.4831725 | 0.83692118 | -8.3402882 | 1.0719E-13 |
| RUBCNL | -1.4808443 | 2.27139472 | -5.8618386 | 3.7318E-08 |
| EPB41L3 | -1.4780148 | 1.32137313 | -4.0613715 | 8.494E-05 |
| S100A9 | -1.4762066 | 11.6515039 | -3.5014848 | 0.00063966 |
| CALCRL | -1.4754404 | 1.09983342 | -7.7956508 | 2.0365E-12 |
| CD52 | -1.4738168 | 7.71632364 | -4.8607106 | 3.3954E-06 |
| ALOX5 | -1.473658 | 5.72437962 | -4.1991238 | 5.011E-05 |
| TMEM273 | -1.4736074 | 2.19677128 | -4.5856024 | 1.0722E-05 |
| MT1X | -1.4730493 | 3.95977795 | -5.9339875 | 2.6487E-08 |
| BAG3 | -1.4711693 | 0.81819331 | -6.8527747 | 2.8281E-10 |
| PTAFR | -1.4694254 | 3.81093959 | -3.635327 | 0.00040232 |
| CLDN10 | -1.4691586 | 0.61752999 | -6.8077422 | 3.5571E-10 |
| MCTP1 | -1.4672879 | 4.36674108 | -6.7637775 | 4.447E-10 |
| PAWR | -1.4663976 | 1.1136094 | -5.6885629 | 8.4245E-08 |
| RXRA | -1.4618753 | 3.56666917 | -5.7505901 | 6.3038E-08 |
| GGT7 | -1.4575391 | 2.37943942 | -7.8663146 | 1.3946E-12 |
| TGM3 | -1.4560662 | 0.14141846 | -7.8097029 | 1.8889E-12 |
| LILRB3 | -1.4556611 | 4.65523918 | -3.8940173 | 0.00015869 |
| ADGRG3 | -1.4555243 | 4.45873314 | -4.2653105 | 3.8725E-05 |
| LMNA | -1.4545702 | 5.74045597 | -4.1948861 | 5.0939E-05 |
| CHD7 | -1.4531874 | 2.46813367 | -6.6750181 | 6.9664E-10 |
| SH3BP4 | -1.4503564 | 0.17087305 | -7.9070485 | 1.1206E-12 |
| ADGRG6 | -1.4494408 | 0.28352078 | -5.636084 | 1.0753E-07 |
| CCDC149 | -1.4486727 | 1.80560761 | -5.4398589 | 2.6482E-07 |
| GAS2L3 | -1.4482234 | 0.85425307 | -8.2020874 | 2.2749E-13 |
| DEFA4 | -1.4451121 | 6.3752044 | -2.7939818 | 0.00601609 |
| TMEM176B | -1.4448523 | 3.77442222 | -2.6889166 | 0.00813359 |
| PF4 | -1.4433825 | 2.46871389 | -3.2132558 | 0.00166468 |
| TRO | -1.4405727 | 0.3199636 | -7.1817399 | 5.1976E-11 |
| COCH | -1.438133 | 2.03427584 | -5.5113189 | 1.9112E-07 |
| CTSH | -1.4374401 | 5.27156561 | -3.4351318 | 0.00080133 |
| LILRB4 | -1.4367937 | 3.17460156 | -2.8054578 | 0.00581823 |
| DTX4 | -1.4363302 | 1.87198945 | -5.003608 | 1.8384E-06 |
| HMGN5 | -1.436249 | 2.37475163 | -6.5873034 | 1.0828E-09 |
| SPINDOC | -1.4357017 | 3.02994721 | -7.4689926 | 1.155E-11 |
| KLF5 | -1.4341844 | 2.35124864 | -4.3366429 | 2.9246E-05 |
| FAM221A | -1.4339685 | 1.44966659 | -8.9199496 | 4.4134E-15 |
| IL1R1 | -1.4338263 | 1.20554022 | -6.3965068 | 2.8001E-09 |
| RHOC | -1.4328412 | 4.35860191 | -5.7608043 | 6.0089E-08 |
| LHFPL6 | -1.4314142 | 0.53423168 | -9.7383226 | 4.5557E-17 |
| NOD2 | -1.4302993 | 2.85379199 | -3.843813 | 0.00019075 |
| SULF2 | -1.4285021 | 3.66691856 | -3.1469675 | 0.00205707 |
| ITPRIPL2 | -1.4234181 | 1.72286541 | -6.70385 | 6.023E-10 |
| DBNDD2 | -1.4232776 | 2.66114856 | -5.3458977 | 4.0517E-07 |
| CDA | -1.4213615 | 4.67588428 | -3.3981999 | 0.0009072 |
| AIFM3 | -1.4200326 | 3.56492106 | -5.4648253 | 2.3636E-07 |
| SPINK2 | -1.4191692 | 5.53006786 | -3.3321415 | 0.00112998 |
| MMP14 | -1.4191671 | 1.07293587 | -5.4766115 | 2.2399E-07 |
| ABR | -1.4188751 | 5.29404111 | -10.069141 | 7.0609E-18 |
| RRAS | -1.4186602 | 3.2100839 | -4.4785054 | 1.6583E-05 |
| DLG5 | -1.4171621 | 1.56284776 | -8.8644423 | 6.0026E-15 |
| SORCS2 | -1.4148646 | 0.43023548 | -8.7315696 | 1.2513E-14 |
| CCDC136 | -1.4140721 | 2.3279815 | -5.5872701 | 1.3477E-07 |
| TRERF1 | -1.4139258 | 3.04242078 | -8.2952839 | 1.37E-13 |
| DNTT | -1.4133341 | 1.26308334 | -3.0796483 | 0.00254209 |
| SAPCD2 | -1.4112204 | 1.4539632 | -9.091474 | 1.7021E-15 |
| ALDH3A2 | -1.4096086 | 3.90597762 | -7.720809 | 3.0375E-12 |
| AQP9 | -1.4083038 | 3.35215876 | -3.1101124 | 0.0023108 |
| STX2 | -1.4064949 | 3.12066398 | -6.5710757 | 1.1745E-09 |
| MAP1LC3A | -1.4058275 | 2.44759346 | -4.4937882 | 1.5589E-05 |
| LRMDA | -1.405738 | 4.45182038 | -5.4325311 | 2.738E-07 |
| CA2 | -1.4057184 | 5.77049281 | -3.3817945 | 0.00095832 |
| CT45A1 | -1.40066 | 0.16901672 | -5.1401422 | 1.0128E-06 |
| ADGRG1 | -1.3989059 | 3.80975329 | -4.5647242 | 1.168E-05 |
| S100A8 | -1.3960972 | 11.4585786 | -3.3747037 | 0.00098124 |
| PRKCA | -1.3947184 | 1.65498479 | -7.1573503 | 5.8995E-11 |
| TFEB | -1.3946341 | 3.42498514 | -4.9115175 | 2.7333E-06 |
| BCL2L11 | -1.3942015 | 3.37022248 | -5.6827097 | 8.6574E-08 |
| SLC2A3 | -1.3941024 | 6.06430712 | -5.6605447 | 9.5981E-08 |
| PVR | -1.3925401 | 2.5806537 | -7.174794 | 5.3886E-11 |
| CEACAM19 | -1.3923789 | 1.46621242 | -9.4535775 | 2.2532E-16 |
| ANKRD37 | -1.3912194 | 1.66076873 | -7.7356753 | 2.8059E-12 |
| RECK | -1.3898294 | 1.87996689 | -8.3018935 | 1.3216E-13 |
| KCNK5 | -1.3885444 | 2.16097975 | -4.9408555 | 2.41E-06 |
| BCL9 | -1.3855212 | 1.09134377 | -8.0194089 | 6.118E-13 |
| FGR | -1.3809419 | 6.78038175 | -4.1288191 | 6.5695E-05 |
| HRH2 | -1.3805353 | 3.51135489 | -4.4040217 | 2.2371E-05 |
| AGAP3 | -1.3798687 | 3.5715476 | -5.857117 | 3.8162E-08 |
| JHY | -1.3794049 | 1.07015281 | -9.2255154 | 8.0647E-16 |
| TMEM8B | -1.3778618 | 1.27511106 | -8.552346 | 3.3566E-14 |
| SMARCD3 | -1.3769553 | 3.83937893 | -6.5228841 | 1.4944E-09 |
| HTR7 | -1.3763147 | 0.99100149 | -5.3233674 | 4.4837E-07 |
| ARHGEF11 | -1.3751776 | 2.86347758 | -4.4052356 | 2.2263E-05 |
| BST1 | -1.3738605 | 5.17039737 | -4.6219734 | 9.2323E-06 |
| ANO7 | -1.3719813 | 0.58065612 | -6.463554 | 2.0082E-09 |
| SLC22A4 | -1.3666696 | 3.35650255 | -5.9316692 | 2.6782E-08 |
| PLEC | -1.364502 | 4.2227117 | -5.5991308 | 1.2759E-07 |
| KLC2 | -1.3620674 | 1.79117832 | -9.1791508 | 1.0445E-15 |
| HCAR3 | -1.3606509 | 2.44442114 | -3.8149093 | 0.0002119 |
| DTNA | -1.360487 | 0.53302926 | -6.4093255 | 2.628E-09 |
| IL13RA1 | -1.3601185 | 3.52727662 | -3.5839931 | 0.00048132 |
| STARD4 | -1.3588689 | 2.91133827 | -5.7939863 | 5.1412E-08 |
| RGPD6 | -1.3573127 | 1.23713043 | -8.4091018 | 7.3598E-14 |
| MSRB1 | -1.3565832 | 5.18116247 | -6.6769766 | 6.898E-10 |
| NLRP12 | -1.355872 | 2.05348971 | -4.0002575 | 0.00010694 |
| GRIP1 | -1.3549921 | 0.99521888 | -6.2992746 | 4.5214E-09 |
| APOBEC3A | -1.3522854 | 4.96143032 | -3.1322679 | 0.002155 |
| NCF1 | -1.3517946 | 5.96603295 | -3.6035804 | 0.00044959 |
| FAM110B | -1.3508858 | 0.93855463 | -5.7841523 | 5.3847E-08 |
| CREB5 | -1.350823 | 2.22806277 | -4.5023981 | 1.5055E-05 |
| TRIP10 | -1.3507464 | 1.74701875 | -7.7580775 | 2.4895E-12 |
| MYOF | -1.3504761 | 1.85049939 | -3.6602848 | 0.0003685 |
| CAST | -1.3491995 | 6.10221365 | -6.9103262 | 2.1077E-10 |
| LMO4 | -1.348636 | 4.0782778 | -5.3923083 | 3.2858E-07 |
| ASGR1 | -1.3474642 | 1.78490855 | -4.5893633 | 1.0558E-05 |
| SPR | -1.3474339 | 0.61223789 | -9.4699783 | 2.0554E-16 |
| UNC13B | -1.3469241 | 0.29284136 | -8.9300503 | 4.173E-15 |
| SERPING1 | -1.346211 | 3.64042317 | -3.758454 | 0.00025982 |
| CACNA2D4 | -1.3458362 | 3.98700207 | -3.9616141 | 0.00012355 |
| CLTCL1 | -1.3456002 | 1.90452657 | -4.5627541 | 1.1774E-05 |
| CCR1 | -1.3435103 | 2.68140774 | -3.1299621 | 0.00217075 |
| TNNT1 | -1.3425254 | 1.16498751 | -4.0158502 | 0.00010086 |
| NRG4 | -1.3416732 | 3.78848347 | -5.6251994 | 1.1309E-07 |
| ACSS2 | -1.3416592 | 4.5754275 | -7.2156679 | 4.3566E-11 |
| NFAM1 | -1.3401474 | 3.38445436 | -3.5174302 | 0.00060567 |
| PPA1 | -1.3395061 | 6.1909807 | -9.7069216 | 5.4356E-17 |
| METRNL | -1.3394819 | 5.08657435 | -4.8935159 | 2.9521E-06 |
| CAMK2D | -1.3387252 | 1.3209155 | -5.2947516 | 5.0978E-07 |
| ADAM8 | -1.3385383 | 5.29547946 | -5.2107393 | 7.4135E-07 |
| TLR5 | -1.3371104 | 1.29569152 | -5.0248714 | 1.6764E-06 |
| AL928654.3 | -1.3356029 | 2.00308296 | -5.3093592 | 4.7747E-07 |
| PPP1R27 | -1.3348249 | 1.9027044 | -4.0733079 | 8.1181E-05 |
| CLEC4E | -1.3342298 | 2.75169396 | -2.9593899 | 0.0036802 |
| SPATA6 | -1.3325823 | 1.67045956 | -6.9105895 | 2.1049E-10 |
| YBX3 | -1.330873 | 7.53923632 | -6.317772 | 4.1286E-09 |
| PRKAR2B | -1.3281121 | 3.26535369 | -4.9777652 | 2.0557E-06 |
| HOXA2 | -1.3278174 | 0.2195413 | -11.161736 | 1.4515E-20 |
| F5 | -1.3265062 | 1.38396429 | -5.3078113 | 4.808E-07 |
| PELI1 | -1.3239461 | 4.44320581 | -4.4824133 | 1.6324E-05 |
| PDZD7 | -1.3234048 | 1.0561132 | -9.204679 | 9.0588E-16 |
| PPARG | -1.3226377 | 0.39869484 | -6.6257759 | 8.9263E-10 |
| SNX33 | -1.3180047 | 1.79248439 | -7.8797188 | 1.2978E-12 |
| SLC26A6 | -1.3174072 | 3.99703699 | -7.4776035 | 1.1037E-11 |
| HMOX1 | -1.3173351 | 3.87329394 | -3.0868717 | 0.00248539 |
| CD24 | -1.3164017 | 5.28312765 | -3.0079848 | 0.00317319 |
| GPAT3 | -1.3157783 | 1.86643397 | -4.9934573 | 1.9209E-06 |
| ALS2CL | -1.3153796 | 1.12327375 | -6.8114898 | 3.49E-10 |
| CYFIP1 | -1.3137878 | 4.00242306 | -7.4709732 | 1.143E-11 |
| BTG1 | -1.3116434 | 7.83497879 | -7.3849248 | 1.7978E-11 |
| OPLAH | -1.3096592 | 0.84314126 | -8.3423634 | 1.0598E-13 |
| CD276 | -1.3091031 | 0.40446945 | -7.1988645 | 4.7548E-11 |
| C8orf88 | -1.308842 | 0.36597002 | -5.8710219 | 3.5729E-08 |
| TMSB15B | -1.3087798 | 1.66760675 | -5.3637288 | 3.7388E-07 |
| CAVIN2 | -1.3018783 | 1.79757237 | -4.5578964 | 1.201E-05 |
| UBTD1 | -1.301826 | 2.20997317 | -6.5393054 | 1.3768E-09 |
| KCNE1 | -1.2992563 | 1.64655518 | -4.6378605 | 8.6463E-06 |
| PDXK | -1.2985526 | 4.89553082 | -7.0543254 | 1.0055E-10 |
| TPM2 | -1.2980346 | 3.47368587 | -5.782693 | 5.4217E-08 |
| HP | -1.297713 | 4.41254209 | -3.2045168 | 0.00171209 |
| GSAP | -1.2976466 | 3.97911498 | -6.2063133 | 7.1253E-09 |
| ZFP57 | -1.2969866 | 0.53251633 | -4.4928083 | 1.5651E-05 |
| ANO8 | -1.29528 | 2.20488797 | -8.1459302 | 3.0856E-13 |
| CD40 | -1.294903 | 1.33963714 | -6.5700599 | 1.1805E-09 |
| SEMA4C | -1.2942529 | 2.70393382 | -5.878676 | 3.4455E-08 |
| SRGN | -1.2931327 | 10.7645975 | -5.4008426 | 3.1613E-07 |
| NACC2 | -1.2921464 | 2.03421451 | -5.38095 | 3.459E-07 |
| P2RY2 | -1.2901952 | 2.66023051 | -4.5809735 | 1.0928E-05 |
| ANXA2 | -1.290042 | 6.64107126 | -3.5927813 | 0.00046683 |
| FCAR | -1.2896662 | 4.66041689 | -3.0057837 | 0.00319468 |
| MAP3K6 | -1.2883876 | 2.8632927 | -6.5497646 | 1.3067E-09 |
| CHST15 | -1.288161 | 2.17395205 | -3.4963748 | 0.00065093 |
| CST3 | -1.2872239 | 7.94112242 | -3.8908459 | 0.00016056 |
| CD1D | -1.2871923 | 2.24912549 | -2.8530408 | 0.00505961 |
| CCDC170 | -1.2870745 | 0.35572729 | -8.0285227 | 5.8242E-13 |
| GAA | -1.2857866 | 5.31195555 | -6.084738 | 1.2852E-08 |
| ADCY6 | -1.2828471 | 1.47973495 | -5.5784143 | 1.404E-07 |
| KIF1C | -1.2826187 | 2.72001534 | -5.7286354 | 6.9866E-08 |
| TMEM9 | -1.281686 | 4.05654966 | -8.2074562 | 2.2095E-13 |
| TRPM4 | -1.2815907 | 1.0984775 | -5.9222661 | 2.8008E-08 |
| SLC2A9 | -1.2793352 | 2.89556944 | -4.5337681 | 1.3253E-05 |
| EHD4 | -1.2787346 | 1.73864863 | -6.2140988 | 6.8599E-09 |
| SLC22A5 | -1.2781691 | 2.37791529 | -8.9420442 | 3.9045E-15 |
| CRIP1 | -1.2780915 | 7.62934799 | -4.0934976 | 7.5183E-05 |
| ANKRD18B | -1.2778486 | 0.81320895 | -5.9577085 | 2.3653E-08 |
| IL2RA | -1.2757327 | 1.04608417 | -4.9128918 | 2.7173E-06 |
| ECM1 | -1.2737199 | 1.56686888 | -6.1216325 | 1.0752E-08 |
| PI3 | -1.2733482 | 0.75098648 | -5.1618603 | 9.2034E-07 |
| GNA12 | -1.2717277 | 3.85988895 | -9.3997425 | 3.0459E-16 |
| CLEC4D | -1.2713669 | 1.39722059 | -4.0715121 | 8.1736E-05 |
| NFKB2 | -1.2705837 | 4.16258197 | -6.1919932 | 7.6402E-09 |
| FOSL2 | -1.2698149 | 6.73524284 | -6.7652674 | 4.4135E-10 |
| ZNF532 | -1.2695357 | 1.33970735 | -5.3826317 | 3.4328E-07 |
| NID1 | -1.2694054 | 0.98085366 | -4.5162156 | 1.4234E-05 |
| PHACTR3 | -1.2689366 | 0.63976794 | -6.0893276 | 1.257E-08 |
| SDC2 | -1.2686717 | 0.25680966 | -6.5545585 | 1.2757E-09 |
| HLX | -1.2676881 | 4.19173883 | -5.0781656 | 1.3291E-06 |
| TPD52 | -1.267217 | 2.15195677 | -5.171354 | 8.8256E-07 |
| SCN9A | -1.2641226 | 0.46360047 | -5.1206809 | 1.1033E-06 |
| SNCAIP | -1.2639242 | 0.15150192 | -5.8147139 | 4.6628E-08 |
| SMPDL3A | -1.2638643 | 0.73677762 | -4.6905553 | 6.949E-06 |
| SPIRE1 | -1.2630235 | 0.58334589 | -8.2987142 | 1.3447E-13 |
| PTK6 | -1.2628078 | 0.44036896 | -8.191957 | 2.4036E-13 |
| ZC2HC1A | -1.2617182 | 0.33140227 | -8.3247087 | 1.167E-13 |
| CRIP3 | -1.2590661 | 0.73868022 | -5.9725062 | 2.2038E-08 |
| TRPS1 | -1.2587779 | 2.40140422 | -4.07399 | 8.0971E-05 |
| ADAP1 | -1.2577184 | 4.4209767 | -5.0374538 | 1.5872E-06 |
| JCHAIN | -1.2571158 | 5.73562098 | -4.2363423 | 4.3364E-05 |
| SLC29A2 | -1.2569146 | 0.73827117 | -8.2141697 | 2.1304E-13 |
| SH3BP5 | -1.2556999 | 3.57602793 | -4.5090005 | 1.4657E-05 |
| BAIAP2 | -1.2527302 | 2.84123448 | -5.7406744 | 6.6037E-08 |
| LILRB2 | -1.2524739 | 5.07949989 | -3.1381179 | 0.00211552 |
| OCLN | -1.2520052 | 1.70357406 | -5.7435175 | 6.5163E-08 |
| NAMPT | -1.2518757 | 6.84937075 | -3.9943288 | 0.00010934 |
| UPP1 | -1.2500222 | 4.77019699 | -3.905044 | 0.00015237 |
| SULT1C4 | -1.249982 | 1.16120562 | -5.2629433 | 5.8768E-07 |
| KRT17 | -1.2487924 | 0.90589596 | -5.414915 | 2.9659E-07 |
| NXF3 | -1.2483755 | 2.3204377 | -3.7001994 | 0.00031994 |
| HLA-F | -1.2483527 | 6.44472835 | -5.0983476 | 1.2168E-06 |
| RTN2 | -1.2449914 | 2.41463904 | -8.5693798 | 3.0568E-14 |
| MPP3 | -1.244171 | 0.36194597 | -6.9513049 | 1.7085E-10 |
| PRXL2A | -1.2424888 | 0.55484162 | -6.6532221 | 7.7751E-10 |
| CALN1 | -1.2420981 | 0.43746385 | -5.6106654 | 1.2096E-07 |
| NLRP1 | -1.2415612 | 4.2468218 | -4.450225 | 1.8587E-05 |
| ITGAM | -1.2412488 | 6.32495626 | -4.2211596 | 4.6003E-05 |
| PPFIBP2 | -1.2400823 | 1.99411201 | -4.9437713 | 2.38E-06 |
| PLCD3 | -1.2396372 | 1.85046588 | -6.9310658 | 1.8953E-10 |
| C11orf45 | -1.2391492 | 0.95922673 | -6.8204121 | 3.3351E-10 |
| CSRP1 | -1.2383354 | 5.06560132 | -9.6606433 | 7.0506E-17 |
| CXCL1 | -1.238202 | 2.39407565 | -3.3347951 | 0.00112012 |
| MYO1C | -1.2340183 | 1.36136584 | -6.2574908 | 5.5493E-09 |
| PPBP | -1.2338872 | 3.07074011 | -2.7117807 | 0.00762234 |
| SAMD11 | -1.2321099 | 0.47151701 | -6.2066745 | 7.1128E-09 |
| TOM1L1 | -1.2320605 | 0.95213865 | -6.1580806 | 9.0101E-09 |
| PDK4 | -1.2301308 | 0.64396178 | -4.4620082 | 1.7725E-05 |
| BMERB1 | -1.2300663 | 0.6495361 | -8.321584 | 1.187E-13 |
| LAMC1 | -1.2297594 | 0.5714144 | -5.4883215 | 2.1232E-07 |
| LBX2 | -1.2286997 | 1.74046404 | -6.5329507 | 1.4212E-09 |
| TMEM150B | -1.2286213 | 2.60503729 | -3.9020969 | 0.00015404 |
| CD300LB | -1.2286174 | 3.13251135 | -2.823024 | 0.00552685 |
| PIEZO2 | -1.2280292 | 1.30207415 | -6.3144444 | 4.1967E-09 |
| PPARGC1A | -1.2259123 | -0.0464774 | -8.3039844 | 1.3066E-13 |
| AFDN | -1.2246715 | 0.90862642 | -6.3541519 | 3.4515E-09 |
| ECE1 | -1.2239839 | 3.13050335 | -4.8066128 | 4.2709E-06 |
| CAPN5 | -1.2229804 | 0.69826286 | -8.871196 | 5.7823E-15 |
| BEND6 | -1.2224282 | 0.65652322 | -7.3828959 | 1.817E-11 |
| PLK3 | -1.2223418 | 5.31608851 | -7.261934 | 3.423E-11 |
| STRIP2 | -1.221422 | 0.85603048 | -7.143973 | 6.3235E-11 |
| LILRA6 | -1.2212635 | 3.13604913 | -2.8422902 | 0.00522263 |
| MDGA1 | -1.2211506 | 1.99901058 | -3.3561116 | 0.0010438 |
| C3AR1 | -1.2204973 | 3.78965816 | -4.047417 | 8.9545E-05 |
| CDH2 | -1.2201476 | -0.0481135 | -5.799667 | 5.0055E-08 |
| CCL1 | -1.2195375 | 0.54243796 | -3.7751572 | 0.00024467 |
| SLC49A3 | -1.2171539 | 2.43024795 | -4.8724736 | 3.2295E-06 |
| DTX3 | -1.215771 | 1.27077985 | -4.8037909 | 4.3221E-06 |
| FOSL1 | -1.2154275 | 2.99111494 | -5.2585613 | 5.9928E-07 |
| ALPL | -1.2146645 | 0.99726754 | -4.5663626 | 1.1602E-05 |
| NRGN | -1.2139966 | 3.6682003 | -3.2140469 | 0.00166045 |
| PSTPIP1 | -1.2107274 | 5.2517046 | -5.6328654 | 1.0914E-07 |
| PHLDA2 | -1.2105466 | 0.54829496 | -5.9473097 | 2.4857E-08 |
| RASSF4 | -1.2103969 | 4.43777189 | -3.3955559 | 0.00091526 |
| PER1 | -1.2092879 | 6.66168928 | -5.8794748 | 3.4325E-08 |
| MYL9 | -1.2075728 | 1.00755233 | -4.687721 | 7.0314E-06 |
| DGAT2 | -1.2074384 | 1.35698673 | -5.0428202 | 1.5506E-06 |
| SEPTIN8 | -1.2058231 | 2.08171211 | -6.8721344 | 2.5621E-10 |
| EMILIN2 | -1.2037306 | 5.17405831 | -6.0951792 | 1.222E-08 |
| PDGFD | -1.2030137 | 1.19877681 | -6.1939949 | 7.5661E-09 |
| CACHD1 | -1.2015284 | 0.02454135 | -9.4093004 | 2.8872E-16 |
| STX1A | -1.2013956 | 0.81268383 | -6.2387117 | 6.0831E-09 |
| RAB11FIP5 | -1.2013217 | 0.5817244 | -6.1647665 | 8.7222E-09 |
| SAGE1 | -1.2010973 | -0.0485551 | -5.5531045 | 1.5776E-07 |
| IRX5 | -1.2007331 | -0.0085445 | -7.0293536 | 1.1437E-10 |
| DDIT4 | -1.2002155 | 5.76596295 | -4.6353336 | 8.737E-06 |
| NOL3 | -1.2001878 | 2.11438225 | -6.8013469 | 3.6747E-10 |
| ZCCHC14 | -1.1998052 | 1.26584805 | -8.8500567 | 6.5002E-15 |
| SH3TC2 | -1.1990958 | 1.00108251 | -6.0987747 | 1.2009E-08 |
| MYO7A | -1.1985506 | 0.7636794 | -4.5161816 | 1.4236E-05 |
| RAP1GAP | -1.1982577 | 1.42480383 | -2.9255516 | 0.00407617 |
| GFI1 | -1.196898 | 4.83129266 | -5.8921567 | 3.232E-08 |
| AL365205.1 | -1.1967504 | 2.11452313 | -7.4320295 | 1.4034E-11 |
| PPFIBP1 | -1.194486 | 3.02008703 | -6.0695573 | 1.3828E-08 |
| HK1 | -1.1943149 | 5.17029088 | -6.2278846 | 6.4135E-09 |
| ARHGEF3 | -1.1937163 | 4.17777888 | -6.9469516 | 1.7471E-10 |
| CFP | -1.1934035 | 6.64918413 | -3.9421204 | 0.00013284 |
| S1PR3 | -1.193361 | 1.0123402 | -5.0051343 | 1.8262E-06 |
| IL6ST | -1.1897565 | 3.32891169 | -5.2555208 | 6.0746E-07 |
| CNTNAP3C | -1.1893335 | 0.2048239 | -5.9550511 | 2.3955E-08 |
| CXCR4 | -1.1887581 | 8.09828951 | -5.1975059 | 7.8615E-07 |
| SLC24A3 | -1.1833819 | 0.42497143 | -6.1302658 | 1.0312E-08 |
| HNRNPLL | -1.1831763 | 3.58450449 | -4.3883307 | 2.3817E-05 |
| FBXO44 | -1.1831394 | 2.99828498 | -6.9269622 | 1.9356E-10 |
| HEXB | -1.182473 | 5.72026245 | -5.763455 | 5.9346E-08 |
| ADCY4 | -1.1823403 | 2.27304703 | -7.1811104 | 5.2146E-11 |
| PHKA1 | -1.1822789 | -0.0566618 | -8.3339618 | 1.1095E-13 |
| MPZL2 | -1.1817931 | 0.74939802 | -5.2117462 | 7.3804E-07 |
| EVI2A | -1.1812677 | 4.89349805 | -5.2327538 | 6.7228E-07 |
| CSTB | -1.180828 | 6.04448767 | -5.5858579 | 1.3566E-07 |
| CDC42EP1 | -1.1803929 | 0.69775551 | -5.0000304 | 1.8671E-06 |
| BACH2 | -1.1792978 | 1.07190099 | -6.2666426 | 5.3061E-09 |
| KLF2 | -1.178786 | 6.29792095 | -4.6671938 | 7.6574E-06 |
| NHSL2 | -1.1765613 | 2.378245 | -4.7785033 | 4.8086E-06 |
| CRY1 | -1.1762885 | 2.58848369 | -7.2548497 | 3.5519E-11 |
| TRIM32 | -1.1759255 | 0.55453095 | -7.976508 | 7.7107E-13 |
| TMEM231 | -1.1754163 | 1.01556826 | -8.7004488 | 1.4856E-14 |
| L3MBTL3 | -1.1740426 | 2.36694084 | -8.2111199 | 2.166E-13 |
| HIP1 | -1.1728882 | 3.62435461 | -5.5739527 | 1.4331E-07 |
| PFKFB3 | -1.1723897 | 5.52349508 | -4.5771091 | 1.1102E-05 |
| TNFRSF1B | -1.1723708 | 5.16741589 | -3.5279737 | 0.00058414 |
| ZFYVE9 | -1.1722981 | 0.14102129 | -7.8954906 | 1.1924E-12 |
| PADI4 | -1.171797 | 5.9851852 | -3.7948453 | 0.00022788 |
| ACSL1 | -1.1714971 | 5.58659527 | -4.6413952 | 8.5209E-06 |
| ALDOC | -1.1710311 | 2.35099969 | -6.476411 | 1.8838E-09 |
| PILRA | -1.1704225 | 5.0091598 | -3.6909244 | 0.00033065 |
| USP44 | -1.1699809 | 0.21286977 | -5.4804216 | 2.2013E-07 |
| IL4I1 | -1.1684621 | 0.72711136 | -5.8998638 | 3.1158E-08 |
| CLCN5 | -1.1675937 | 0.83870889 | -8.3132749 | 1.2421E-13 |
| ZFP3 | -1.1627401 | 1.22704135 | -6.8459073 | 2.9289E-10 |
| ACVR2A | -1.1613468 | 0.39651134 | -8.8527068 | 6.4056E-15 |
| FOXP4 | -1.1610555 | 2.33034881 | -10.109512 | 5.6216E-18 |
| GCAT | -1.1606091 | 2.23287521 | -5.0808007 | 1.3139E-06 |
| TMEM144 | -1.159998 | 1.94458472 | -5.8980818 | 3.1423E-08 |
| EFCC1 | -1.159219 | 0.29655172 | -6.2742268 | 5.1126E-09 |
| JPH4 | -1.158609 | 0.65860492 | -6.6626329 | 7.4152E-10 |
| AC055839.2 | -1.1581933 | 2.136415 | -4.6325569 | 8.8378E-06 |
| CRIP2 | -1.156702 | 2.2287099 | -3.9382872 | 0.00013474 |
| HDGFL3 | -1.1528139 | 1.48031215 | -4.0806945 | 7.8935E-05 |
| SCUBE1 | -1.1501682 | 2.0315467 | -3.8743456 | 0.00017059 |
| AVPI1 | -1.149833 | 1.82060223 | -5.5796366 | 1.3961E-07 |
| SCARA3 | -1.1494144 | -0.0821392 | -7.1930386 | 4.901E-11 |
| SLC43A2 | -1.1493119 | 3.66536283 | -4.1346159 | 6.4252E-05 |
| PKN3 | -1.1489641 | 1.89045755 | -6.10902 | 1.1429E-08 |
| CNIH4 | -1.1485477 | 4.441866 | -6.839034 | 3.0333E-10 |
| ASIC3 | -1.1481928 | 1.00494022 | -7.2450627 | 3.7379E-11 |
| CEBPB | -1.1477874 | 4.56843257 | -4.5116378 | 1.4501E-05 |
| VSIR | -1.1471956 | 7.53481691 | -4.8051114 | 4.2981E-06 |
| AC002996.1 | -1.147004 | 0.68437101 | -4.8853747 | 3.0566E-06 |
| SIRPD | -1.1467262 | 1.06345934 | -4.2831083 | 3.6116E-05 |
| CT45A9 | -1.1449159 | 0.00761218 | -5.0775186 | 1.3329E-06 |
| GSTM3 | -1.1444858 | 1.62845073 | -4.2998835 | 3.3812E-05 |
| RHOU | -1.1430404 | 2.33359707 | -4.1892407 | 5.2064E-05 |
| HES4 | -1.1426571 | 0.71864079 | -5.4683386 | 2.3261E-07 |
| PLPP3 | -1.1415083 | 0.26568269 | -9.570895 | 1.1671E-16 |
| TNFSF9 | -1.1413866 | 1.5070598 | -4.4083286 | 2.1989E-05 |
| ANXA3 | -1.1408655 | 4.59297872 | -2.6838831 | 0.00825023 |
| UST | -1.1400887 | 0.46835849 | -8.0336178 | 5.6662E-13 |
| BHLHE40 | -1.1394856 | 5.6555294 | -5.6706426 | 9.1578E-08 |
| L3MBTL4 | -1.1389786 | 0.443531 | -6.028716 | 1.6832E-08 |
| SNAI1 | -1.1338715 | 1.83345019 | -4.044119 | 9.0668E-05 |
| ATP6V0E2 | -1.133522 | 2.64349482 | -6.0292021 | 1.6793E-08 |
| RIN1 | -1.1334752 | 1.62564624 | -5.0562279 | 1.4626E-06 |
| HBEGF | -1.1328117 | 3.49247677 | -3.3869335 | 0.00094203 |
| NAGA | -1.1322898 | 4.42370813 | -5.0365104 | 1.5937E-06 |
| TM6SF1 | -1.1304474 | 2.25459631 | -4.4652618 | 1.7494E-05 |
| OXCT1 | -1.1278392 | 2.97675766 | -7.7401675 | 2.7394E-12 |
| SPINT1 | -1.1276245 | 2.42729161 | -4.6554152 | 8.0406E-06 |
| LFNG | -1.1276237 | 3.87005539 | -4.3800238 | 2.4619E-05 |
| JAML | -1.1270298 | 5.29241567 | -2.7407608 | 0.00701628 |
| KCNH3 | -1.1264158 | 1.07690932 | -5.755859 | 6.15E-08 |
| PGGHG | -1.1261256 | 4.40425875 | -6.5131658 | 1.5687E-09 |
| TAMALIN | -1.1250803 | 3.71933996 | -3.1666989 | 0.00193211 |
| MS4A3 | -1.1244399 | 5.5796291 | -2.9389603 | 0.0039148 |
| ZC3H12A | -1.1234757 | 4.92729754 | -5.3900698 | 3.3193E-07 |
| ST14 | -1.1227454 | 1.42879556 | -3.5491042 | 0.00054314 |
| IER3 | -1.1218463 | 4.49847396 | -2.7529396 | 0.00677492 |
| KCNC3 | -1.1164858 | -0.0631821 | -7.2452043 | 3.7351E-11 |
| CITED4 | -1.1164237 | 3.45547593 | -5.0877081 | 1.2748E-06 |
| SH3TC1 | -1.1161437 | 4.4802115 | -4.0257508 | 9.7171E-05 |
| MANSC1 | -1.1154272 | 1.31881006 | -5.5722819 | 1.4442E-07 |
| CTSB | -1.1153303 | 7.34315946 | -4.872713 | 3.2262E-06 |
| HOPX | -1.1149377 | 3.76640095 | -2.8848728 | 0.00460383 |
| HVCN1 | -1.1137902 | 3.69385351 | -4.0435094 | 9.0877E-05 |
| PLPPR2 | -1.1137845 | 3.55761402 | -5.7859708 | 5.3388E-08 |
| CARD9 | -1.1137822 | 4.74756905 | -4.4029655 | 2.2466E-05 |
| MUC12 | -1.1121157 | 0.26384123 | -7.1417918 | 6.3954E-11 |
| RASAL2 | -1.1115289 | 0.5695822 | -7.3615304 | 2.0327E-11 |
| PTPRJ | -1.111445 | 3.08180866 | -4.0594334 | 8.5565E-05 |
| PIP4P2 | -1.1107966 | 4.31193644 | -5.6948818 | 8.1799E-08 |
| MAP3K5 | -1.1098519 | 3.30807913 | -7.7346987 | 2.8205E-12 |
| ZNF467 | -1.1098437 | 3.17537274 | -3.8508182 | 0.00018593 |
| PELI3 | -1.1092114 | 1.41643299 | -6.2281354 | 6.4057E-09 |
| TSHZ3 | -1.1081891 | 0.31698142 | -6.6582721 | 7.5799E-10 |
| PLIN5 | -1.1080002 | 1.11608642 | -6.2738955 | 5.1209E-09 |
| SECTM1 | -1.1068168 | 3.45969094 | -2.6889851 | 0.00813201 |
| CEBPE | -1.1057735 | 2.71618418 | -3.7292662 | 0.00028846 |
| TTLL7 | -1.1054003 | 0.2981097 | -7.9393839 | 9.4171E-13 |
| BACE1 | -1.1048527 | 2.97517455 | -6.7624712 | 4.4766E-10 |
| GPR183 | -1.1046984 | 4.77862535 | -3.508348 | 0.00062482 |
| SLC6A9 | -1.103409 | 1.14509152 | -3.3733666 | 0.00098562 |
| CCDC3 | -1.1011542 | 0.00825622 | -5.837644 | 4.1844E-08 |
| CTDSPL | -1.1000304 | 1.43834302 | -4.9122387 | 2.7249E-06 |
| ACOT4 | -1.0999584 | 0.4407131 | -8.7872683 | 9.1993E-15 |
| CES4A | -1.0999213 | 1.04312699 | -4.3489739 | 2.7852E-05 |
| PNOC | -1.0989925 | 0.60678964 | -6.7161096 | 5.6611E-10 |
| IGLL5 | -1.0987912 | 3.99154777 | -3.0900905 | 0.0024605 |
| STAB1 | -1.0958039 | 5.40958124 | -3.0572321 | 0.00272577 |
| RAB44 | -1.0932268 | 4.56414353 | -4.5184611 | 1.4104E-05 |
| KLK1 | -1.0922412 | 0.08649325 | -7.1238622 | 7.0183E-11 |
| SCN8A | -1.0912069 | 0.37212794 | -7.7803622 | 2.21E-12 |
| COL5A1 | -1.0902561 | 0.19225953 | -5.2989176 | 5.0035E-07 |
| PTPRF | -1.0901451 | 0.41101839 | -5.6026237 | 1.2555E-07 |
| VANGL1 | -1.0896136 | 1.72974562 | -4.8418598 | 3.6786E-06 |
| TBC1D9 | -1.0893496 | 1.18489611 | -4.0227179 | 9.8287E-05 |
| OBSCN | -1.0886628 | 2.40638387 | -4.7215716 | 6.1058E-06 |
| SGCB | -1.0878742 | 0.17125122 | -8.3031437 | 1.3126E-13 |
| PDLIM2 | -1.0877625 | 4.96550898 | -8.0939907 | 4.0882E-13 |
| LRRC20 | -1.0874568 | 1.52913372 | -8.9059043 | 4.7708E-15 |
| CHST2 | -1.0868093 | 2.351123 | -4.4577527 | 1.8032E-05 |
| CPD | -1.0866812 | 4.70147533 | -5.8868241 | 3.3149E-08 |
| MGST2 | -1.0864678 | 5.798161 | -7.5708207 | 6.7424E-12 |
| PRR16 | -1.0856713 | -0.0852506 | -7.0880104 | 8.4493E-11 |
| LILRA1 | -1.0852352 | 3.15807707 | -3.004571 | 0.00320658 |
| SRC | -1.0841142 | 2.69672197 | -4.579009 | 1.1016E-05 |
| IGSF10 | -1.0827675 | 2.40340718 | -2.7706985 | 0.00643648 |
| GALNT12 | -1.080714 | 0.92524321 | -8.0503645 | 5.1761E-13 |
| EGR2 | -1.0790056 | 0.74498612 | -5.8640386 | 3.6931E-08 |
| SKIDA1 | -1.0784377 | 0.98651293 | -5.6531251 | 9.9349E-08 |
| RBFOX2 | -1.0765076 | -0.0567012 | -8.2005702 | 2.2938E-13 |
| HOXA1 | -1.076315 | 0.25714718 | -8.5229266 | 3.945E-14 |
| SCO2 | -1.0759187 | 3.98306693 | -3.9527392 | 0.0001277 |
| ARAP3 | -1.0757321 | 1.59622537 | -4.0836285 | 7.806E-05 |
| PPFIA4 | -1.0753896 | 0.83017732 | -4.791947 | 4.5437E-06 |
| BCAM | -1.0745383 | 1.26356001 | -5.016536 | 1.7382E-06 |
| MAPRE3 | -1.0724005 | 1.45968062 | -6.0879152 | 1.2656E-08 |
| ADAMTS1 | -1.0716984 | 0.06464992 | -4.9069589 | 2.7872E-06 |
| BEND4 | -1.0702363 | 0.05266501 | -4.8233906 | 3.9783E-06 |
| SIRPB1 | -1.069522 | 4.19425898 | -2.7555529 | 0.00672412 |
| EGR1 | -1.0689902 | 5.44049171 | -3.6498451 | 0.0003823 |
| COL6A1 | -1.0686374 | 0.27159004 | -5.9964457 | 1.9652E-08 |
| PDZK1IP1 | -1.0685499 | 1.62733875 | -2.7363965 | 0.00710466 |
| SCPEP1 | -1.0636193 | 6.04467104 | -3.1223923 | 0.0022232 |
| CCDC24 | -1.0614813 | 2.33688876 | -6.4039184 | 2.6993E-09 |
| CCDC74A | -1.0600736 | 0.20877267 | -6.7723124 | 4.2586E-10 |
| UNC119 | -1.059383 | 4.92314045 | -6.4047066 | 2.6888E-09 |
| STARD8 | -1.0587029 | 1.65015701 | -4.7591103 | 5.2172E-06 |
| BACE2 | -1.0584126 | 0.57930488 | -5.218488 | 7.1628E-07 |
| SLC35G1 | -1.0578598 | 0.21983404 | -9.2919754 | 5.5646E-16 |
| AP1S3 | -1.0576933 | 1.32858303 | -7.1123673 | 7.4489E-11 |
| MS4A14 | -1.0576179 | 1.92094236 | -2.9849312 | 0.0034051 |
| ABHD5 | -1.0573388 | 4.74552693 | -5.0789353 | 1.3246E-06 |
| ZNF697 | -1.0548759 | 0.51610241 | -8.9014692 | 4.8895E-15 |
| C2orf81 | -1.0537021 | 0.36537615 | -9.1442777 | 1.2685E-15 |
| LYPD6B | -1.0530746 | -0.1407826 | -7.1438466 | 6.3277E-11 |
| FAM83A | -1.0525771 | 0.48482841 | -5.0277584 | 1.6555E-06 |
| BATF3 | -1.0524946 | 0.78334764 | -5.1979778 | 7.8451E-07 |
| DCHS1 | -1.0513546 | 0.22034904 | -7.0616898 | 9.6802E-11 |
| FAR1 | -1.0512467 | 4.51178001 | -5.0133844 | 1.7621E-06 |
| LRP10 | -1.0508432 | 5.05367364 | -6.9581497 | 1.6495E-10 |
| AL162231.1 | -1.050427 | 0.74524199 | -6.7859847 | 3.973E-10 |
| JUNB | -1.0493963 | 7.89413333 | -4.6439314 | 8.432E-06 |
| ACP3 | -1.048923 | 2.9799865 | -4.0412469 | 9.1656E-05 |
| DDR1 | -1.0483442 | 0.58996801 | -7.12226 | 7.0768E-11 |
| GPRIN3 | -1.0480133 | 0.98480066 | -5.4709429 | 2.2986E-07 |
| RUNX1 | -1.0477836 | 6.07138839 | -7.2774986 | 3.1558E-11 |
| GPC4 | -1.0463619 | -0.1387299 | -5.411954 | 3.006E-07 |
| COL18A1 | -1.0452032 | 2.66592567 | -5.771124 | 5.7247E-08 |
| SESTD1 | -1.044717 | 4.30977499 | -4.4640299 | 1.7581E-05 |
| COL9A2 | -1.043892 | 3.9838767 | -4.1583533 | 5.8653E-05 |
| PAQR8 | -1.043594 | 1.65566584 | -5.9477139 | 2.4809E-08 |
| REEP3 | -1.0435606 | 2.3231238 | -5.842723 | 4.0851E-08 |
| ARHGEF5 | -1.0429158 | 0.66541499 | -6.2497652 | 5.7631E-09 |
| PMM1 | -1.0423263 | 3.09742716 | -5.1825546 | 8.3994E-07 |
| CACNB4 | -1.0419068 | 1.11986826 | -6.0124677 | 1.8198E-08 |
| NKX3-1 | -1.0411922 | 0.0441943 | -8.6973019 | 1.5116E-14 |
| GPR157 | -1.0409311 | 0.33477845 | -11.074572 | 2.3794E-20 |
| HDAC4 | -1.0401698 | 3.41209491 | -5.7486238 | 6.3622E-08 |
| HBZ | -1.0395834 | 0.25023854 | -3.6176626 | 0.00042801 |
| CGREF1 | -1.0384648 | 0.39866108 | -6.6893747 | 6.4797E-10 |
| SLC37A3 | -1.0374039 | 2.40739077 | -4.7506009 | 5.4069E-06 |
| RTN1 | -1.0365341 | 0.26222527 | -5.5061886 | 1.9566E-07 |
| CBX6 | -1.0352715 | 3.18432125 | -8.1612461 | 2.8396E-13 |
| MCEMP1 | -1.0348101 | 4.01452239 | -2.8897673 | 0.00453719 |
| ANO9 | -1.0345324 | 2.01665372 | -6.5285737 | 1.4526E-09 |
| QPCT | -1.0339123 | 2.36594363 | -2.8418873 | 0.00522884 |
| PSAT1 | -1.0336819 | 0.46099756 | -5.5705525 | 1.4558E-07 |
| MORN4 | -1.033497 | 0.44411365 | -6.1944686 | 7.5487E-09 |
| CIBAR1 | -1.0334005 | 2.64884834 | -4.4971633 | 1.5378E-05 |
| BCL2A1 | -1.0327548 | 6.20770705 | -2.9419768 | 0.00387932 |
| LILRB1 | -1.0321839 | 3.15965627 | -2.7512846 | 0.00680727 |
| PLEKHH3 | -1.0312902 | 1.48814577 | -4.518845 | 1.4082E-05 |
| SLC26A11 | -1.0306427 | 1.74581515 | -4.2276546 | 4.4856E-05 |
| GLIPR2 | -1.0302272 | 5.72021773 | -4.7379595 | 5.7011E-06 |
| RASGRP3 | -1.029411 | 4.39950952 | -3.910126 | 0.00014954 |
| METRN | -1.0292671 | 2.00213819 | -6.0131407 | 1.814E-08 |
| MECOM | -1.0289509 | 0.6747841 | -4.0630579 | 8.4399E-05 |
| SLC16A9 | -1.0288151 | 0.5053233 | -5.4413523 | 2.6303E-07 |
| SLAMF1 | -1.0282899 | 1.40744382 | -4.9098247 | 2.7532E-06 |
| SGSH | -1.0278009 | 3.30513711 | -2.9003893 | 0.00439558 |
| CDH23 | -1.0276304 | 1.54574017 | -5.0133973 | 1.762E-06 |
| ITGB5 | -1.0272703 | 1.38338741 | -4.5156161 | 1.4268E-05 |
| SLC35F6 | -1.0246975 | 1.86570334 | -7.8747286 | 1.333E-12 |
| TANC2 | -1.0245972 | 2.38818296 | -5.1236028 | 1.0892E-06 |
| HEBP2 | -1.0237505 | 4.72075284 | -10.124746 | 5.1582E-18 |
| CR1L | -1.0230638 | 1.02767553 | -3.2666494 | 0.00140056 |
| FFAR2 | -1.022892 | 2.54399612 | -3.463508 | 0.00072798 |
| BTBD19 | -1.0226441 | 1.55466618 | -5.650375 | 1.0063E-07 |
| TSLP | -1.0217542 | -0.140436 | -6.85461 | 2.8018E-10 |
| FXYD2 | -1.0217195 | 0.85854651 | -5.697968 | 8.0629E-08 |
| CBX7 | -1.0215418 | 2.77201039 | -6.6703842 | 7.1311E-10 |
| CUEDC1 | -1.0211264 | 2.53265476 | -4.3773809 | 2.488E-05 |
| S100A6 | -1.0207496 | 9.55077488 | -4.2991016 | 3.3916E-05 |
| PAPLN | -1.0200099 | 1.13895428 | -7.8386608 | 1.6175E-12 |
| AHNAK | -1.0198371 | 6.64291486 | -4.3108373 | 3.2384E-05 |
| PTPRS | -1.0196547 | 0.28135907 | -6.578641 | 1.1308E-09 |
| FCGRT | -1.0194598 | 5.91794296 | -3.1646484 | 0.00194476 |
| AIFM2 | -1.0192274 | 1.0863062 | -8.8457842 | 6.6558E-15 |
| SLC37A2 | -1.0186917 | 1.59039484 | -3.0882287 | 0.00247487 |
| FBN2 | -1.0185846 | 0.27395987 | -5.455661 | 2.4645E-07 |
| OLFML2A | -1.0185165 | 0.08721249 | -7.4988157 | 9.8682E-12 |
| NAV1 | -1.0180933 | 0.90321125 | -5.7598382 | 6.0362E-08 |
| TMEM170B | -1.0178509 | 3.14076849 | -3.5769249 | 0.00049328 |
| ADRB1 | -1.0175759 | 0.08485136 | -7.3634887 | 2.0119E-11 |
| SPOCK1 | -1.0172964 | 0.16432911 | -6.1006054 | 1.1903E-08 |
| MARCKS | -1.0168994 | 3.56603348 | -2.7848764 | 0.00617742 |
| TNS1 | -1.01625 | 2.69108014 | -3.1507845 | 0.00203233 |
| ZDHHC11B | -1.0155994 | 0.31714288 | -5.558747 | 1.5371E-07 |
| THBS4 | -1.0154485 | 2.19550459 | -3.4346056 | 0.00080275 |
| NLRP3 | -1.0151334 | 4.5048548 | -4.435108 | 1.9751E-05 |
| PEA15 | -1.0142619 | 4.96800864 | -6.0704993 | 1.3766E-08 |
| PLK2 | -1.0138109 | 3.14465001 | -4.6839966 | 7.1412E-06 |
| MT1G | -1.0131762 | 0.24635938 | -4.4602742 | 1.785E-05 |
| IL6R | -1.0124339 | 4.56217953 | -5.6980783 | 8.0588E-08 |
| MTMR11 | -1.0121939 | 1.61724516 | -2.9088409 | 0.00428582 |
| LPL | -1.0115224 | 0.83285442 | -4.0713899 | 8.1774E-05 |
| BMF | -1.0110933 | 3.76895265 | -5.6741271 | 9.0104E-08 |
| PDLIM7 | -1.0107947 | 5.25803505 | -6.7181337 | 5.6034E-10 |
| VSIG2 | -1.0089908 | 0.92782924 | -4.3336334 | 2.9596E-05 |
| PXDN | -1.0077925 | 0.48415352 | -4.2703589 | 3.7967E-05 |
| CHIT1 | -1.0077803 | 1.14489074 | -3.7756999 | 0.00024419 |
| FLOT1 | -1.0077317 | 5.96937942 | -5.9081401 | 2.9956E-08 |
| MDK | -1.0074079 | 3.41649462 | -2.9883098 | 0.00337016 |
| GRINA | -1.0073162 | 6.4313664 | -8.2048132 | 2.2415E-13 |
| EGR3 | -1.0065182 | 1.47289363 | -4.1888073 | 5.2151E-05 |
| DPP4 | -1.0065105 | 0.72520056 | -5.3051295 | 4.8662E-07 |
| USP27X | -1.005984 | 0.46147513 | -9.9661279 | 1.2627E-17 |
| MFAP4 | -1.0058585 | 1.67206273 | -3.6242672 | 0.00041822 |
| ETV5 | -1.004721 | 1.27736945 | -5.4997963 | 2.0147E-07 |
| PHLPP2 | -1.0037533 | 1.16807352 | -5.5356468 | 1.7094E-07 |
| ANKS6 | -1.0033965 | 1.85077942 | -6.2752807 | 5.0862E-09 |
| FCGR2B | -1.0032432 | 2.4302797 | -2.9097956 | 0.00427358 |
| THEM5 | -1.001132 | 0.14821128 | -4.4101941 | 2.1826E-05 |
| KRT1 | -1.0002582 | 0.11660458 | -3.3210093 | 0.00117222 |
| TESC | 1.00395862 | 5.95471411 | 4.70372106 | 6.5781E-06 |
| PTGS1 | 1.00679169 | 4.30725072 | 5.75881357 | 6.0653E-08 |
| CD48 | 1.00743723 | 6.00940124 | 3.79359505 | 0.00022891 |
| CMPK2 | 1.00761917 | 3.95878994 | 5.87957546 | 3.4309E-08 |
| ZG16B | 1.0077559 | 3.33408671 | 4.89984441 | 2.8733E-06 |
| ADGRA3 | 1.00790781 | 3.01356436 | 6.15210496 | 9.2753E-09 |
| TAF4B | 1.00864245 | 2.42279349 | 7.5018365 | 9.712E-12 |
| PLPP1 | 1.00978075 | 1.4349073 | 5.14178559 | 1.0055E-06 |
| TEX30 | 1.01096769 | 4.56455956 | 5.84152539 | 4.1083E-08 |
| C2CD2 | 1.01225049 | 3.36704253 | 7.71976901 | 3.0544E-12 |
| LAX1 | 1.01576031 | 2.31513206 | 5.83887402 | 4.1601E-08 |
| PIP5K1B | 1.01964851 | 3.1090365 | 5.55656407 | 1.5526E-07 |
| OSCP1 | 1.02163405 | 2.23165203 | 6.7457378 | 4.8728E-10 |
| CENPV | 1.02200093 | 4.64077892 | 7.04545651 | 1.0526E-10 |
| CRLF2 | 1.02508109 | 0.58159123 | 4.8370796 | 3.754E-06 |
| DFFB | 1.02545585 | 3.48444991 | 12.0027102 | 1.2394E-22 |
| GDPD1 | 1.02687574 | 2.1330847 | 7.52493353 | 8.596E-12 |
| CCDC57 | 1.02737458 | 6.33844645 | 9.45527943 | 2.2318E-16 |
| SLC39A3 | 1.03059757 | 6.07248137 | 7.14376263 | 6.3304E-11 |
| PCDH10 | 1.03109228 | -0.2752789 | 3.55322597 | 0.00053546 |
| SLFN13 | 1.03242595 | 4.35693495 | 6.20478699 | 7.1786E-09 |
| ANGPTL6 | 1.03438895 | 2.23096728 | 5.34615734 | 4.0469E-07 |
| ATP13A4 | 1.0351015 | 1.05355378 | 7.41904083 | 1.5026E-11 |
| ADH6 | 1.03860636 | 0.71932141 | 7.44974472 | 1.2783E-11 |
| LPAR4 | 1.03862192 | 1.60775701 | 5.85485404 | 3.8573E-08 |
| MCTP2 | 1.03902581 | 5.11129795 | 5.7032519 | 7.8666E-08 |
| ANKRD33B | 1.03963079 | 1.68236579 | 6.43210009 | 2.3476E-09 |
| HLA-DRB5 | 1.04087142 | 6.53095553 | 2.76349527 | 0.00657185 |
| SMIM1 | 1.04363174 | 3.0408776 | 4.75052042 | 5.4087E-06 |
| DNAAF4 | 1.04646065 | 1.84159314 | 8.06187385 | 4.8639E-13 |
| SLC40A1 | 1.04769633 | 5.4122484 | 4.2995762 | 3.3853E-05 |
| RPS6KL1 | 1.04996999 | 1.54566278 | 5.91467048 | 2.904E-08 |
| H2BC9 | 1.05037014 | 1.2842633 | 4.31896235 | 3.1363E-05 |
| CDKN1C | 1.05275725 | 2.91866087 | 5.63029112 | 1.1045E-07 |
| HMGN3 | 1.0531806 | 6.82958405 | 10.08601 | 6.4195E-18 |
| STMN1 | 1.05343692 | 7.83427035 | 6.41169076 | 2.5974E-09 |
| LDB1 | 1.05350869 | 6.78971723 | 9.61802411 | 8.9578E-17 |
| ADD2 | 1.05378271 | 3.40727604 | 3.40483242 | 0.00088727 |
| HACD1 | 1.0543377 | 4.34395213 | 6.05182539 | 1.5062E-08 |
| DNMT3A | 1.05531533 | 5.24965206 | 8.26378166 | 1.6265E-13 |
| MARCKSL1 | 1.05561513 | 5.65337691 | 4.49671326 | 1.5406E-05 |
| APOE | 1.05691267 | 2.16936006 | 4.03652165 | 9.3305E-05 |
| B3GALNT1 | 1.05891065 | 3.10015552 | 6.20146534 | 7.2957E-09 |
| B3GNT2 | 1.06047186 | 5.07030004 | 8.85741323 | 6.2409E-15 |
| AC099489.1 | 1.06069105 | 2.61853545 | 3.77010936 | 0.00024915 |
| AMIGO3 | 1.06342601 | 1.32544181 | 6.20697446 | 7.1024E-09 |
| PROSER2 | 1.06544002 | 2.08854961 | 5.61324565 | 1.1953E-07 |
| CERCAM | 1.06939549 | 3.42343703 | 6.18033404 | 8.0864E-09 |
| MPL | 1.07001323 | 2.02162436 | 4.43775993 | 1.9542E-05 |
| HLA-DPA1 | 1.07039942 | 8.35339839 | 3.04123617 | 0.00286427 |
| AFF3 | 1.07110538 | 3.64952647 | 4.94394102 | 2.3783E-06 |
| PIR | 1.0717833 | 1.31751511 | 4.21086109 | 4.788E-05 |
| HLA-DOA | 1.07330373 | 3.76376442 | 4.26405092 | 3.8917E-05 |
| RGS1 | 1.07356942 | 5.553515 | 3.05366762 | 0.00275609 |
| ALS2 | 1.07721478 | 4.24463846 | 7.83311872 | 1.6663E-12 |
| SPACA9 | 1.0788563 | 1.93896815 | 7.56822517 | 6.8358E-12 |
| CDK6 | 1.08282433 | 5.63622587 | 6.95747278 | 1.6553E-10 |
| TOX | 1.08315015 | 2.3657452 | 5.70938717 | 7.6444E-08 |
| CEP41 | 1.0878986 | 3.46958205 | 9.28652429 | 5.7367E-16 |
| PRKCQ | 1.08897012 | 3.24220603 | 6.67344486 | 7.0219E-10 |
| MLLT11 | 1.08897509 | 2.20485429 | 5.65236565 | 9.97E-08 |
| C12orf75 | 1.09044645 | 3.82509992 | 5.80021834 | 4.9925E-08 |
| LXN | 1.09313262 | 4.69452944 | 6.07382954 | 1.3546E-08 |
| HHIP | 1.09340734 | 0.37076946 | 3.93716745 | 0.0001353 |
| TPSB2 | 1.09584013 | 3.31848416 | 3.55617351 | 0.00053004 |
| LEF1 | 1.0962513 | 3.46716298 | 3.5245029 | 0.00059115 |
| NEXN | 1.09953181 | 1.43178423 | 6.33636698 | 3.7676E-09 |
| MPZL1 | 1.10014938 | 5.21758135 | 8.7814541 | 9.4996E-15 |
| CIITA | 1.10054754 | 6.11467172 | 4.69932456 | 6.6998E-06 |
| BANK1 | 1.1029729 | 3.15674344 | 6.34694914 | 3.5763E-09 |
| HES6 | 1.10385639 | 2.87143896 | 4.77662138 | 4.8468E-06 |
| CABLES1 | 1.10425524 | 2.18142934 | 4.53772027 | 1.3041E-05 |
| AC073111.4 | 1.10845787 | 3.71706386 | 8.02943476 | 5.7956E-13 |
| MBOAT1 | 1.1112742 | 3.71439496 | 11.3889448 | 4.0029E-21 |
| KIF17 | 1.11333782 | 0.85029824 | 5.313902 | 4.6784E-07 |
| RHOH | 1.11484034 | 5.03237423 | 5.02476776 | 1.6772E-06 |
| AGR2 | 1.11646491 | 0.25817503 | 3.67812978 | 0.00034598 |
| TSEN54 | 1.11733112 | 5.23865605 | 7.65473106 | 4.3188E-12 |
| CD84 | 1.12097025 | 5.89981942 | 10.6852519 | 2.1627E-19 |
| HLA-DPB1 | 1.12150489 | 7.55670328 | 3.40063614 | 0.00089983 |
| ADAMTS14 | 1.1309996 | 0.80743289 | 5.53171946 | 1.7405E-07 |
| PTPRCAP | 1.13296782 | 6.28310434 | 5.59834439 | 1.2805E-07 |
| LRRC34 | 1.13783622 | 2.41180408 | 7.84038235 | 1.6027E-12 |
| GTF3C5 | 1.13866901 | 6.45744553 | 10.4759144 | 7.0791E-19 |
| LPAR6 | 1.14120713 | 3.95911503 | 3.95456672 | 0.00012684 |
| C21orf62 | 1.14484281 | 0.12306944 | 8.53491837 | 3.6937E-14 |
| PLD4 | 1.14724972 | 4.57921186 | 4.660436 | 7.875E-06 |
| S100Z | 1.14807951 | 4.11868192 | 4.92982879 | 2.5269E-06 |
| ST7 | 1.15085408 | 3.82216514 | 5.83747222 | 4.1878E-08 |
| PREX2 | 1.15234896 | 0.04355934 | 3.72643701 | 0.00029139 |
| AGPAT5 | 1.15725177 | 4.5069556 | 9.10726608 | 1.5589E-15 |
| TFF3 | 1.15809359 | 1.19430127 | 3.01502883 | 0.00310529 |
| ANKRD27 | 1.15894957 | 4.71152364 | 10.1978923 | 3.4121E-18 |
| PRKAR1B | 1.1609967 | 3.28910199 | 9.23246225 | 7.758E-16 |
| GRAMD4 | 1.16264541 | 5.49357069 | 8.36770924 | 9.2283E-14 |
| SERPINF1 | 1.16653198 | 4.33672838 | 6.72395455 | 5.4409E-10 |
| LRP12 | 1.16800939 | 2.30028032 | 6.68594941 | 6.5927E-10 |
| ZFY | 1.17077188 | 1.08960997 | 2.83148555 | 0.0053913 |
| BAHCC1 | 1.17210546 | 6.06735459 | 5.33672477 | 4.2224E-07 |
| NT5M | 1.17642446 | 4.05852391 | 6.39775967 | 2.7828E-09 |
| CHST12 | 1.18209801 | 4.4787957 | 6.69687566 | 6.2389E-10 |
| CDKN2A | 1.18307638 | 3.26593855 | 4.81561182 | 4.1114E-06 |
| KIFAP3 | 1.19230826 | 2.96745712 | 11.790096 | 4.1258E-22 |
| ILDR2 | 1.19601645 | 1.12666993 | 6.89240986 | 2.31E-10 |
| PIK3R6 | 1.19619812 | 4.19836931 | 6.47378649 | 1.9086E-09 |
| APOC4-APOC2 | 1.19848223 | 1.01812237 | 5.34040891 | 4.153E-07 |
| CPXM1 | 1.20321484 | 5.83057555 | 4.86852413 | 3.2843E-06 |
| C21orf91 | 1.20352086 | 4.13599136 | 8.56227708 | 3.1784E-14 |
| P2RY1 | 1.20674701 | 2.67226507 | 8.38185984 | 8.5418E-14 |
| HIP1R | 1.20999614 | 3.19588976 | 6.63043566 | 8.7196E-10 |
| ITGA4 | 1.21372627 | 7.68195896 | 9.81863628 | 2.8991E-17 |
| TNNT3 | 1.21469059 | 3.75679211 | 5.09704112 | 1.2238E-06 |
| PKLR | 1.215217 | 0.81645638 | 3.92362512 | 0.00014227 |
| ZBED3 | 1.21860098 | 2.63829801 | 7.33578003 | 2.3264E-11 |
| CYP2E1 | 1.21936365 | 1.28640349 | 6.54220064 | 1.357E-09 |
| KLF1 | 1.22003204 | 3.22199335 | 3.50194339 | 0.00063866 |
| ACCS | 1.2224984 | 6.5339659 | 7.50237939 | 9.6842E-12 |
| MATK | 1.22331607 | 4.64901866 | 6.82276074 | 3.2954E-10 |
| MGLL | 1.22432429 | 4.38275255 | 5.34745863 | 4.0233E-07 |
| MYC | 1.22520667 | 5.7384102 | 6.52493552 | 1.4792E-09 |
| CFI | 1.22617404 | 0.33358815 | 4.58558883 | 1.0723E-05 |
| DEPDC7 | 1.2272451 | 2.36980907 | 7.58940433 | 6.1099E-12 |
| TTC9 | 1.22791778 | 1.25451362 | 7.12467675 | 6.9888E-11 |
| ZBED2 | 1.23186226 | 0.15639359 | 7.57035058 | 6.7593E-12 |
| RAB7B | 1.24773606 | 4.12511916 | 4.34162652 | 2.8675E-05 |
| CYP4F2 | 1.24799095 | 1.91950724 | 5.75739237 | 6.1059E-08 |
| TIE1 | 1.25035915 | 3.14331821 | 6.23847272 | 6.0902E-09 |
| LY9 | 1.25523073 | 2.90843437 | 6.49765704 | 1.6948E-09 |
| CASP3 | 1.26152118 | 5.1901013 | 9.25464914 | 6.8544E-16 |
| SPAG16 | 1.26154627 | 1.71527241 | 7.25494528 | 3.5501E-11 |
| ZMAT3 | 1.26933675 | 3.24387923 | 10.5316442 | 5.1633E-19 |
| GNG7 | 1.2743978 | 2.75915768 | 8.39999922 | 7.7355E-14 |
| DDO | 1.27572145 | 2.00683112 | 7.16140726 | 5.7765E-11 |
| SP140 | 1.27628271 | 3.37381644 | 7.67774929 | 3.8209E-12 |
| NIPSNAP3B | 1.27717849 | 2.82407635 | 10.9158872 | 5.851E-20 |
| VSTM1 | 1.27837786 | 4.80858673 | 3.90147049 | 0.00015439 |
| MAN1A1 | 1.28150901 | 4.6314302 | 6.62016357 | 9.1816E-10 |
| CLIP2 | 1.284751 | 4.71667036 | 8.55937626 | 3.2295E-14 |
| GATA2 | 1.28543349 | 5.58849171 | 4.524252 | 1.3776E-05 |
| GFI1B | 1.29160858 | 3.89341257 | 4.86477481 | 3.3371E-06 |
| TPSAB1 | 1.29462132 | 2.77033511 | 3.49908022 | 0.00064494 |
| MICU3 | 1.29497448 | 1.3061464 | 6.07820661 | 1.3263E-08 |
| CDH26 | 1.29644825 | 1.90699193 | 7.08060563 | 8.779E-11 |
| SPATC1L | 1.29810452 | 3.41783419 | 7.16358612 | 5.7116E-11 |
| ITM2C | 1.29823512 | 6.56435846 | 5.33840114 | 4.1907E-07 |
| KIT | 1.29922852 | 4.73322191 | 5.26141483 | 5.917E-07 |
| SAMD13 | 1.30430557 | 1.92881763 | 9.39370776 | 3.1505E-16 |
| ZNF721 | 1.30806991 | 3.71876793 | 6.25790978 | 5.5379E-09 |
| ASB9 | 1.30917219 | 1.68384049 | 5.91199255 | 2.9412E-08 |
| LRRC75B | 1.31969644 | 2.18963705 | 8.09455389 | 4.0758E-13 |
| ZNF185 | 1.32367716 | 4.52745855 | 4.2893906 | 3.5236E-05 |
| IL2RG | 1.32565173 | 7.37701396 | 7.55416173 | 7.3644E-12 |
| DDIT4L | 1.33216227 | 0.5074037 | 3.76670795 | 0.00025222 |
| CD1C | 1.33767512 | 1.41383405 | 3.98481607 | 0.0001133 |
| FZD6 | 1.3415299 | 1.1967721 | 5.62438116 | 1.1352E-07 |
| FHIT | 1.34732255 | 2.71195059 | 8.65747605 | 1.8826E-14 |
| PLTP | 1.34792169 | 4.03298793 | 8.25916006 | 1.6679E-13 |
| COL14A1 | 1.35391254 | 0.46034445 | 5.51959468 | 1.84E-07 |
| CD69 | 1.35831983 | 7.2098083 | 4.73498794 | 5.7725E-06 |
| CASP6 | 1.36109114 | 5.42142226 | 9.12021963 | 1.4504E-15 |
| GMPR | 1.36150712 | 3.77124539 | 5.08960075 | 1.2643E-06 |
| HOMER2 | 1.3630679 | 2.79990587 | 9.62100663 | 8.809E-17 |
| DIPK1B | 1.3673243 | 3.81482742 | 6.16679876 | 8.6365E-09 |
| PM20D2 | 1.37360375 | 3.79094613 | 9.61105021 | 9.3156E-17 |
| DNAJC12 | 1.38443328 | 1.73714373 | 5.54188586 | 1.6611E-07 |
| LGALS3BP | 1.38611135 | 5.58267302 | 3.93251039 | 0.00013766 |
| GDF11 | 1.39453079 | 2.94523471 | 7.43193994 | 1.404E-11 |
| PALD1 | 1.39580113 | 0.95641438 | 7.68477303 | 3.6807E-12 |
| RAB33A | 1.40076387 | 2.76817143 | 6.36057721 | 3.3439E-09 |
| CD244 | 1.40586081 | 5.5404982 | 8.964315 | 3.4506E-15 |
| MEX3B | 1.40818189 | 3.12090726 | 6.22829686 | 6.4006E-09 |
| GYPC | 1.41243858 | 7.18523568 | 4.62487736 | 9.1224E-06 |
| NKD2 | 1.41569818 | 0.71231845 | 4.86838209 | 3.2862E-06 |
| SOX4 | 1.41867251 | 5.71242473 | 5.4956821 | 2.053E-07 |
| MYCN | 1.44162669 | 4.19473082 | 4.80430932 | 4.3127E-06 |
| UTY | 1.44403678 | 1.32123312 | 3.17928892 | 0.00185611 |
| PDLIM1 | 1.44710858 | 5.6300385 | 4.94742509 | 2.3429E-06 |
| SORL1 | 1.45309707 | 6.36838119 | 8.92365032 | 4.3238E-15 |
| SLA2 | 1.45368902 | 3.76580095 | 6.9394092 | 1.816E-10 |
| RPS6KA2 | 1.46304673 | 2.29232365 | 9.04483995 | 2.2062E-15 |
| SMAD7 | 1.46651319 | 2.62624249 | 6.32777761 | 3.9303E-09 |
| COL23A1 | 1.47756868 | 2.65912911 | 5.2617624 | 5.9078E-07 |
| RHOBTB3 | 1.48852415 | 3.70032721 | 8.41689037 | 7.0529E-14 |
| ST8SIA1 | 1.4984725 | 1.83071812 | 9.79370401 | 3.3359E-17 |
| UGT2A3 | 1.5031355 | 0.01665499 | 7.8718061 | 1.3541E-12 |
| APBA2 | 1.51130752 | 1.88164483 | 6.71945032 | 5.5663E-10 |
| B4GALT6 | 1.51227735 | 3.59519525 | 8.22138224 | 2.0485E-13 |
| LPO | 1.51353199 | 3.30459137 | 5.45257498 | 2.4993E-07 |
| NTRK1 | 1.52093779 | 1.70225951 | 7.40002018 | 1.6607E-11 |
| ACY3 | 1.53516364 | 2.59879486 | 5.80456302 | 4.8914E-08 |
| CSF2RB | 1.53563568 | 4.97378124 | 6.95491334 | 1.6772E-10 |
| HPGD | 1.53930736 | 2.19257748 | 4.95525733 | 2.2652E-06 |
| ZFPM1 | 1.54043778 | 2.80871049 | 7.75013234 | 2.5975E-12 |
| CYYR1 | 1.54522358 | 0.57051766 | 4.08508064 | 7.763E-05 |
| REXO5 | 1.54903917 | 4.26761446 | 9.62174534 | 8.7726E-17 |
| LIME1 | 1.55349177 | 3.7392636 | 7.10182954 | 7.8664E-11 |
| PRKY | 1.55855418 | 1.82751511 | 3.52337088 | 0.00059345 |
| GPR174 | 1.55946034 | 3.55329429 | 7.61141267 | 5.4365E-12 |
| PRR5L | 1.5599433 | 4.4362899 | 6.21043685 | 6.9835E-09 |
| FAM178B | 1.56089939 | 2.00341531 | 4.6195535 | 9.3249E-06 |
| CSF1 | 1.56342568 | 2.9622264 | 5.66091437 | 9.5817E-08 |
| IGFL4 | 1.56758913 | 3.09732351 | 5.65575245 | 9.8143E-08 |
| STAM | 1.57191335 | 4.96364511 | 10.6803028 | 2.2242E-19 |
| RBPMS2 | 1.58292987 | 0.76274812 | 6.05931588 | 1.4528E-08 |
| MAP3K8 | 1.58713895 | 7.01880352 | 7.23493259 | 3.9406E-11 |
| STK32B | 1.59743072 | 1.30365145 | 7.77470721 | 2.2778E-12 |
| ABCB1 | 1.59776315 | 1.93469133 | 6.23401267 | 6.2244E-09 |
| AL121845.2 | 1.59808737 | 4.09463138 | 8.11994856 | 3.5522E-13 |
| EIF1AY | 1.60285066 | 2.02559801 | 2.75533278 | 0.00672838 |
| EMID1 | 1.60492823 | 5.505279 | 5.78243267 | 5.4284E-08 |
| ZBTB16 | 1.61104475 | 4.04408476 | 9.27124183 | 6.2479E-16 |
| EGFL7 | 1.61299045 | 5.70354499 | 5.59679361 | 1.2898E-07 |
| PALM2AKAP2 | 1.61524857 | 4.31781114 | 7.74529153 | 2.6655E-12 |
| CEBPA | 1.63839745 | 6.25044409 | 8.72826367 | 1.2743E-14 |
| CSRP2 | 1.6414752 | 1.12273534 | 7.15535176 | 5.961E-11 |
| GP1BB | 1.64284601 | 2.47381592 | 6.37496946 | 3.1146E-09 |
| SIDT1 | 1.64618926 | 3.1081861 | 9.25590108 | 6.8066E-16 |
| ZNF608 | 1.6489896 | 1.69942711 | 7.84994216 | 1.5226E-12 |
| TFR2 | 1.64930856 | 3.78188615 | 6.47268053 | 1.9191E-09 |
| SYCP2L | 1.65191571 | 1.1370321 | 5.00822726 | 1.8019E-06 |
| SUCNR1 | 1.6695225 | 3.97709655 | 7.09620258 | 8.0988E-11 |
| MPO | 1.67904145 | 10.2581621 | 4.07529009 | 8.0573E-05 |
| TESPA1 | 1.68118396 | 5.14758675 | 7.29749559 | 2.8426E-11 |
| TSPAN13 | 1.68250019 | 3.02420909 | 7.77596228 | 2.2626E-12 |
| SLC27A2 | 1.6859376 | 3.3967154 | 9.77229766 | 3.7631E-17 |
| IL9R | 1.69771577 | 1.97895394 | 7.96991486 | 7.9895E-13 |
| LAMA5 | 1.70376981 | 3.07831799 | 8.25299419 | 1.7249E-13 |
| KCNH2 | 1.70795106 | 2.85355796 | 4.95927533 | 2.2264E-06 |
| LGSN | 1.71507869 | 0.16734349 | 8.91596605 | 4.512E-15 |
| TRIB2 | 1.71802125 | 2.75185734 | 7.35036693 | 2.1552E-11 |
| UGT2B28 | 1.7184084 | 0.71186434 | 7.40733077 | 1.5981E-11 |
| MGAT3 | 1.72010823 | 2.05182412 | 6.82053982 | 3.3329E-10 |
| GSTM2 | 1.72187258 | 5.86620016 | 10.2291213 | 2.86E-18 |
| TP53INP1 | 1.72513003 | 4.91276966 | 10.5905321 | 3.6989E-19 |
| ADAMTS3 | 1.74064316 | 0.36445024 | 7.80422461 | 1.9451E-12 |
| TRAT1 | 1.7545436 | 2.14960556 | 7.75781166 | 2.4931E-12 |
| MSS51 | 1.77539784 | 1.96455085 | 9.66110461 | 7.0323E-17 |
| GALC | 1.7876107 | 4.74573845 | 11.9681679 | 1.5067E-22 |
| APP | 1.80267387 | 4.49823165 | 4.1074629 | 7.1285E-05 |
| STAP1 | 1.81798376 | 3.11306502 | 11.2310885 | 9.7957E-21 |
| CTSE | 1.82533682 | 1.41121582 | 6.46287296 | 2.015E-09 |
| CD200 | 1.85337325 | 3.12809477 | 6.37102561 | 3.1758E-09 |
| MYEF2 | 1.85370617 | 2.72517989 | 7.65756219 | 4.2542E-12 |
| ITGA9 | 1.85512464 | 2.97508139 | 8.70444844 | 1.4532E-14 |
| HBG2 | 1.85981507 | 7.68123553 | 2.85231948 | 0.0050704 |
| SMIM24 | 1.86682097 | 6.92268183 | 5.63773282 | 1.0671E-07 |
| SORCS1 | 1.86800766 | 0.46490613 | 8.04709436 | 5.2683E-13 |
| VPREB1 | 1.87850543 | 1.27569589 | 5.51254791 | 1.9004E-07 |
| NECTIN1 | 1.88357946 | 4.04876487 | 10.2809383 | 2.1337E-18 |
| EHD3 | 1.9041599 | 2.29689175 | 12.8045981 | 1.3518E-24 |
| HENMT1 | 1.91483736 | 4.78760116 | 11.0777257 | 2.3372E-20 |
| WASF1 | 1.91684021 | 3.53024787 | 8.14805874 | 3.0502E-13 |
| CD38 | 1.9329264 | 5.59132736 | 10.1914994 | 3.5376E-18 |
| LDLRAD4 | 1.93768757 | 4.11484588 | 13.1448572 | 2.0094E-25 |
| LTK | 1.94698882 | 3.48987152 | 7.9136771 | 1.0813E-12 |
| SPRY1 | 1.94764856 | 3.01278235 | 6.66726178 | 7.2442E-10 |
| CBX2 | 1.94959764 | 3.16848894 | 7.51980058 | 8.8325E-12 |
| CDCA7 | 1.95993461 | 4.50594464 | 8.74129573 | 1.1859E-14 |
| JUP | 1.96718546 | 4.91939766 | 7.85797794 | 1.4584E-12 |
| B3GNT7 | 1.97586506 | 1.9230562 | 6.42696191 | 2.4082E-09 |
| BAALC | 1.98048164 | 4.02058192 | 5.71367396 | 7.4928E-08 |
| HDC | 2.00096584 | 3.70609668 | 5.40812955 | 3.0586E-07 |
| SLC14A1 | 2.01199441 | 3.40839476 | 7.26396443 | 3.3869E-11 |
| DEPTOR | 2.03632612 | 4.21024433 | 12.3988134 | 1.3249E-23 |
| KEL | 2.03671631 | 3.2407196 | 6.25676396 | 5.5691E-09 |
| HGF | 2.04445978 | 4.79218426 | 6.59815779 | 1.0254E-09 |
| KLHL13 | 2.04860868 | 1.49424214 | 11.2395516 | 9.3368E-21 |
| PYHIN1 | 2.06568112 | 2.72502652 | 10.6481042 | 2.6694E-19 |
| GATA1 | 2.07008345 | 3.83835673 | 6.46879368 | 1.9566E-09 |
| ZNF385C | 2.08586634 | 2.47014636 | 7.97254266 | 7.8772E-13 |
| LRRC28 | 2.09551384 | 4.87957544 | 11.7874725 | 4.1876E-22 |
| ABO | 2.10031626 | 3.32411232 | 5.82116639 | 4.523E-08 |
| POU4F1 | 2.10561737 | 0.79489109 | 4.59683026 | 1.0239E-05 |
| MKRN3 | 2.11886778 | 1.55143261 | 6.58242855 | 1.1096E-09 |
| CTSW | 2.14046603 | 7.03588341 | 8.26866965 | 1.5838E-13 |
| KDM5D | 2.15528035 | 2.42121182 | 3.62764483 | 0.0004133 |
| ST18 | 2.15677821 | 0.89701552 | 9.78970983 | 3.4118E-17 |
| MFSD2B | 2.17927947 | 2.59224417 | 8.31916767 | 1.2028E-13 |
| APBA1 | 2.20373303 | 0.47279704 | 9.18994755 | 9.8344E-16 |
| MYO7B | 2.21740309 | 2.80341216 | 8.12314136 | 3.4912E-13 |
| C1QTNF4 | 2.22158843 | 3.28211035 | 7.76136775 | 2.4462E-12 |
| CYP7B1 | 2.25472201 | 1.73310926 | 10.0137598 | 9.6517E-18 |
| APOC2 | 2.28293391 | 4.01944684 | 5.62816713 | 1.1155E-07 |
| GGT5 | 2.29871477 | 3.06048476 | 8.72640958 | 1.2874E-14 |
| CRACD | 2.30172098 | 2.03478034 | 9.41289442 | 2.8297E-16 |
| NPR3 | 2.33181776 | 4.21450945 | 7.51242456 | 9.1836E-12 |
| CLECL1 | 2.34637958 | 3.81992146 | 7.405617 | 1.6125E-11 |
| DDX3Y | 2.37649871 | 3.02635568 | 3.45375532 | 0.00075245 |
| SPTBN2 | 2.3826867 | 1.46843668 | 10.3207023 | 1.7039E-18 |
| APOC1 | 2.40794475 | 2.98808937 | 6.80419935 | 3.6218E-10 |
| CRYGD | 2.41859767 | 1.61616091 | 8.67309868 | 1.7274E-14 |
| LHFPL2 | 2.43178546 | 3.37323654 | 13.0659391 | 3.1245E-25 |
| GPA33 | 2.43839356 | 3.09237191 | 10.5479685 | 4.7073E-19 |
| KLHL33 | 2.47773961 | 0.80782238 | 11.3732061 | 4.3765E-21 |
| MUC19 | 2.48612928 | 3.09692353 | 7.95047307 | 8.8714E-13 |
| CETP | 2.54117543 | 1.83464587 | 10.9588696 | 4.5855E-20 |
| ZNF385D | 2.62197248 | 1.74595974 | 8.26488184 | 1.6168E-13 |
| CD7 | 2.63566973 | 4.44921644 | 9.3124768 | 4.9623E-16 |
| P2RX5 | 2.64031317 | 5.14488539 | 11.3435211 | 5.1785E-21 |
| PTX4 | 2.67559242 | 1.15666761 | 10.7955281 | 1.1576E-19 |
| PTH2R | 2.68328882 | 3.25111253 | 10.5024382 | 6.0919E-19 |
| LAMP5 | 2.77527302 | 1.48828946 | 6.45172211 | 2.1298E-09 |
| CD34 | 2.79072528 | 5.01355837 | 5.61162421 | 1.2043E-07 |
| PROM1 | 2.84477734 | 4.54771098 | 7.36692314 | 1.9759E-11 |
| RPS4Y1 | 2.85408389 | 3.59423294 | 3.74273974 | 0.00027489 |
| IKZF2 | 2.89313732 | 3.15847211 | 11.3324871 | 5.5128E-21 |
| MEST | 2.89607384 | 5.14254978 | 10.8911849 | 6.7306E-20 |
| PNMT | 2.90253165 | 0.94839662 | 11.4265888 | 3.2338E-21 |
| SVOPL | 2.97479812 | 2.38384251 | 12.3657143 | 1.5966E-23 |
| TMIGD2 | 3.01030777 | 2.97646962 | 10.9751398 | 4.1814E-20 |
| CNRIP1 | 3.0520749 | 2.88892942 | 12.1792291 | 4.5722E-23 |
| S100B | 3.06907621 | 3.38463552 | 11.6195039 | 1.0839E-21 |
| CD96 | 3.15854422 | 5.35150198 | 9.1451891 | 1.2621E-15 |
| ITM2A | 3.1972447 | 6.532172 | 10.3481004 | 1.4593E-18 |
| DHRS3 | 3.28633526 | 2.77289244 | 14.1717071 | 6.7258E-28 |
| CYTL1 | 3.39886392 | 4.87726263 | 9.72314262 | 4.9618E-17 |
| DLC1 | 3.40236298 | 3.11204796 | 15.2357725 | 2.0271E-30 |
| SHD | 3.45045076 | 1.9799137 | 10.6194858 | 3.1393E-19 |
| UMODL1 | 3.53553118 | 2.70193803 | 10.9775881 | 4.1238E-20 |
| RAMP1 | 3.58653761 | 2.69640261 | 9.98167012 | 1.1567E-17 |
| IGLL1 | 3.60027866 | 6.31611273 | 7.59425994 | 5.9546E-12 |
| MYO18B | 3.64491949 | 1.01119705 | 10.1554028 | 4.338E-18 |
| TSPAN7 | 3.82437241 | 1.9798657 | 12.1049969 | 6.9532E-23 |
| ARPP21 | 4.38713364 | 1.4101408 | 14.0259915 | 1.502E-27 |
| HPGDS | 4.58281334 | 3.70042499 | 13.8031119 | 5.1513E-27 |
| UGT2B11 | 4.98970392 | 2.58907463 | 13.7953665 | 5.3772E-27 |
| TRH | 5.3690105 | 3.22033593 | 13.8846739 | 3.2796E-27 |
